# Supplementary material for: Adherence to antiretroviral therapy and its associated factors among children living with HIV in Eastern and Southern Africa: A systematic review and meta-analysis
Source: PLoS One. 2025 Jan 6;20(1):e0312529. doi: 10.1371/journal.pone.0312529 (PMC11703094; doi:10.1371/journal.pone.0312529)
Supplement: S2 Table — (DOCX) [file pone.0312529.s002.docx]

**S2 Table : Search strings used for a comprehensive search in databases**

| Publication Years | Data bases Search results and search strings |
| --- | --- |
|  | **PubMed = 760** |
| **Filters applied: from 1/1/ 2015- 26/2/2024.** | \| (((((((((((((((((((((((((((((((((("anti-retroviral agents"[MeSH Terms]) OR ("antiretroviral therapy, highly active"[MeSH Terms])) ) OR ("adherence to antiretroviral therapy"[Title/Abstract])) AND (HIV[MeSH Terms])) OR ("HIV Infections"[MeSH Terms])) OR ("acquired immunodeficiency syndrome"[Title/Abstract])) AND (Botswana)) OR (Burundi)) OR (Comoros)) OR (Djibouti)) OR (Eritrea)) OR (Eswatini)) OR (Ethiopia)) OR (Kenya)) OR (Lesotho)) OR (Madagascar)) OR (Malawi)) OR (Mauritius)) OR (Mozambique)) OR (Namibia)) OR (Rwanda)) OR (Seychelles)) OR (Somalia)) OR ("South Africa")) OR ("South Sudan")) OR (Swaziland)) OR (Tanzania)) OR (Uganda)) OR (Zambia)) OR (Zimbabwe)) AND (Child*[MeSH Terms])) AND ("Risk Factors"[MeSH Terms])) NOT (Adult[MeSH Terms])) NOT (Adolescents[MeSH Terms]) \| \| --- \| |
| **1/1/2015 - 26/2/2024** | **EMBASE: 148** |
|  | ((adherence OR 'highly active antiretroviral therapy' OR art OR hiv OR 'antiretroviral therapy') AND botswana OR burundi OR comoros OR djibouti OR eritrea OR eswatini OR ethiopia OR kenya OR lesotho OR madagascar OR malawi OR mauritius OR mozambique OR namibia OR rwanda OR seychelles OR somalia OR 'south africa' OR 'south sudan' OR swaziland OR tanzania OR uganda OR zambia OR zimbabwe) AND children AND 'associated factors' AND [english]/lim AND [2015-2024]/py AND 'human immunodeficiency virus infection'/dm |
| **1/1/2015 - 26/2/2024** | **SCOPUS: 141** |
|  | "adherence to antiretroviral therapy" OR "adherence to highly active antiretroviral therapy" OR "adherence to HAART" OR "adherence to ART" AND "children living with HIV" OR "infant living with HIV" AND determinants OR predictors OR "Risk factor" OR "Associated factors" AND Botswana OR Burundi OR Comoros OR Djibouti OR Eritrea OR Eswatini OR Ethiopia OR Kenya OR Lesotho OR Madagascar OR Malawi OR Mauritius OR Mozambique OR Namibia OR Rwanda OR Seychelles OR Somalia OR "South Africa" OR "South Sudan" OR Swaziland OR Tanzania OR Uganda OR Zambia OR Zimbabwe AND PUBYEAR > 2014 AND PUBYEAR < 2025 AND ( LIMIT-TO ( EXACTKEYWORD,"Human Immunodeficiency Virus Infection" ) ) AND ( LIMIT-TO ( DOCTYPE,"ar" ) ) AND ( LIMIT-TO ( LANGUAGE,"English" ) ) |
| **1/1/2015-26/2/2024** | **HINARI: 48** |
|  | ((SubjectTerms:(adherence)) OR (SubjectTerms:("adherence to ART")) OR (SubjectTerms:("antiretroviral therapy"))) AND ((SubjectTerms:(children)) OR (infants)) AND (("africa, eastern") OR ("africa, southern")) |
| **1/1/2015-26/2/2024** | **Google Scholar: 617** |
|  | "adherence to anti-retroviral therapy" Botswana OR Burundi OR Comoros OR Djibouti OR Eritrea OR Eswatini OR Ethiopia OR Kenya OR Lesotho OR Madagascar OR Malawi OR Mauritius OR Mozambique OR Namibia OR Rwanda OR Seychelles OR Somalia OR "South Africa" OR "South Sudan" OR Swaziland OR Tanzania OR Uganda OR Zambia OR Zimbabwe "Children living with HIV"  **We have use these terms in Google Scolar advanced search and we have got the above result**  **with all of the words:** “adherence to anti-retroviral therapy”  **with the exact phrase:** “Children living with HIV”  **with at least one of the words:** Botswana Burundi Comoros Djibouti Eritrea Eswatini Ethiopia Kenya Lesotho Madagascar Malawi Mauritius Mozambique Namibia Rwanda Seychelles Somalia “South Africa” “South Sudan” Swaziland Tanzania Uganda Zambia Zimbabwe  **where my words occur**: anywhere in the article  **Return articles dated between:** 2015-2024 |
