# Supplementary material for: Adherence to antiretroviral therapy and its associated factors among children living with HIV in Eastern and Southern Africa: A systematic review and meta-analysis
Source: PLoS One. 2025 Jan 6;20(1):e0312529. doi: 10.1371/journal.pone.0312529 (PMC11703094; doi:10.1371/journal.pone.0312529)
Supplement: S3 Table — (DOCX) [file pone.0312529.s003.docx]

**S3 Table: quality appraisal results for included cross-sectional, cohort and experimental studies**

| Author, year | Study design | Q1 | Q2 | Q3 | Q4 | Q5 | Q6 | Q7 | Q8 | Q9 | Q10 | Q11 | Risk of bias (RoB) |
| --- | --- | --- | --- | --- | --- | --- | --- | --- | --- | --- | --- | --- | --- |
| Alemayehu, 2023 | Cross-sectional | Yes | Yes | Yes | Yes | Yes | Yes | Yes | Yes |  |  |  | Low RoB |
| Biru, 2017 | Cohort | unclear | Yes | Yes | Yes | Yes | Yes | Yes | No | No | No | No | Moderate RoB |
| Ebrahim, 2017 |  | Yes | Yes | Yes | Yes | No | No | Yes | No |  |  |  | Moderate RoB |
| Echiru, 2017 | Cross-sectional | Yes | Yes | Yes | Yes | No | No | Yes | No |  |  |  | Moderate RoB |
| Feyera, 2016 | Cross-sectional | Yes | Yes | No | Yes | Yes | Yes | Yes | Yes |  |  |  | Low RoB |
| Feyissa, 2017 | Cross-sectional | Yes | Yes | Yes | Yes | No | No | Yes | Yes |  |  |  | Moderate RoB |
| GebreEyesus, 2021 | Cohort | unclear | Yes | Yes | Yes | Yes | Yes | Yes | Yes | No | Yes | No | Moderate RoB |
| Gemechu, 2023 | Cross-sectional | Yes | Yes | Yes | Yes | Yes | Yes | Yes | Yes |  |  |  | Low risk of bias |
| Gultie, 2015 | Cross-sectional | Yes | Yes | Yes | Yes | Yes | Yes | Yes | Yes |  |  |  | Low RoB |
| Gutema, 2019 | Cross-sectional | Yes | Yes | Yes | Yes | No | No | Yes | No |  |  |  | Moderate RoB |
| Guyo, 2023 | Cohort | unclear | Yes | Yes | Yes | Yes | No | Yes | No | No | No | No | Moderate RoB |
| Mukami, 2022 | Cross- sectional | Yes | Yes | Yes | Yes | No | No | Yes | No |  |  |  | Moderate RoB |
| MUGAMBI, 2015 | Cross- sectional | Yes | Yes | Yes | Yes | Yes | Yes | Yes | Yes |  |  |  | Low RoB |
| Mugusi, 2019 | Cross- sectional | Yes | Yes | Yes | Yes | Yes | Yes | No | Yes |  |  |  | Low RoB |
| MUSOVYA, 2020 | Cross- sectional | Yes | Yes | Yes | Yes | Yes | No | No | Yes |  |  |  | Moderate RoB |
| Mussa, 2022 | Cross- sectional | Yes | Yes | Yes | Yes | Yes | Yes | Yes | Yes |  |  |  | Low RoB |
| Mwiti, 2023 | Cross-sectional | Yes | Yes | Yes | No | Yes | Yes | Yes | Yes |  |  |  | Low RoB |
| Opiyo, 2022 | Expermental | unclear | Yes | Yes | Yes | No | Yes | Yes | No |  |  |  | Moderate RoB |
| Smith,2016 | Cohort | unclear | Yes | Yes | Yes | Yes | Yes | Yes | Yes | No | No | Yes | Moderate RoB |
| Ssanyu,2020 | Cross-sectional | Yes | Yes | Yes | Yes | Yes | Yes | Yes | No |  |  |  | Low RoB |
| Talam,2015 | Cross-sectional | Yes | Yes | Yes | Yes | No | No | Yes | No |  |  |  | Moderate RoB |
| Tesfahunegn et.al, 2023 | Cross-sectional | Yes | Yes | Yes | Yes | Yes | Yes | Yes | Yes |  |  |  | Low RoB |
| Tong, 2020 | Cross-sectional | Yes | Yes | Yes | Yes | No | No | Yes | Yes |  |  |  | Low RoB |
| Urassa, 2018 | Cross-sectional | Yes | Yes | Yes | No | Yes | Yes | Yes | Yes |  |  |  | Low RoB |
| Van Elsland, 2018 | Cross-sectional | Yes | Yes | No | No | Yes | Yes | Yes | Yes |  |  |  | Moderate RoB |
| Vreeman et.al, 2015 | Cohort | unclear | Yes | Yes | No | No | Yes | Yes | Yes | Yes | Yes | No | Moderate RoB |
| Wadunde, 2018 | Cross-sectional | Yes | No | Yes | Yes | Yes | Yes | Yes | Yes |  |  |  | Low RoB |
| Zegeye, 2015 | Cross-sectional | Yes | Yes | Yes | Yes | Yes | Yes | Yes | Yes |  |  |  | Low RoB |

Interpretation Q1-11 represents the items used for the quality assessment

Cross-sectional (total 8 items); 7-8 LoB, 4-6 moderate RoB; 0-3 high RoB

Cohort (total 11 items) ); 9-11 LoB, 5-8 moderate RoB; 0-4 high RoB

Experimental (total items: 8); 7-8, low RoB; 4-6, uncear RoB; 0-3, high RoB.
