# Supplementary material for: Adherence to antiretroviral therapy and its associated factors among children living with HIV in Eastern and Southern Africa: A systematic review and meta-analysis
Source: PLoS One. 2025 Jan 6;20(1):e0312529. doi: 10.1371/journal.pone.0312529 (PMC11703094; doi:10.1371/journal.pone.0312529)
Supplement: S4 Table — (DOCX) [file pone.0312529.s004.docx]

S4 Table: Reasons for excluded primary studies

| N^o^ | Author, year | Title | Reference URL | Main reason for  exclusion |
| --- | --- | --- | --- | --- |
| 1. | Abera, 2015 | Factors Influencing Adherence to Antiretroviral Therapy among People Living With HIV/AIDS at ART Clinic in Jimma University Teaching Hospital, Southwest Ethiopia | <https://www.researchgate.net/publication/374337954> | Adult population |
| 2 | Abadiga, 2020 | Adherence to antiretroviral therapy and associated factors among Human immunodeficiency virus positive patients accessing treatment at Nekemte referral hospital, west Ethiopia | <https://doi.org/10.1371/journal.pone.0232703> | All ART patients on-treatment follow up  i.e. Not only children<15 years |
| 3 | Anthony, 2017 | Role of social workers in antiretroviral Therapy (Art) Adherence To Children’s Living With Hiv/Aids: A Case Of Temeke Municipal (Pasada) | [https://www.semanticscholar.org/paper/Role-of-Social-Workers-in-Antiretroviral-Therapy-to Anthony/8b6142528ba4d56f4473774c1671119120d84d8](https://www.semanticscholar.org/paper/Role-of-Social-Workers-in-Antiretroviral-Therapy-to%20Anthony/8b6142528ba4d56f4473774c1671119120d84d8)f | Qualitative study |
| 4 | Apomdi et.al, 2021 | Barriers to ART adherence among school students living with HIV in Kenya | <https://doi.org/10.2989/16085906.2021.1979606> | Qualitative study  Age group 13-17 years |
| 5. | BAKEIHA, 2015 | Organisation factors and adherence to anti-retroviral therapy Among hiv/aids patients at kisenyi health centre, kampala capital city authority | <https://umispace.umi.ac.ug/handle/20.500.12305/623?show=full> | Adult population |
| 6. | Beshir, 2017 | Factors affecting adherence to antiretroviral treatment among patients living with HIV/AIDS, in Bale zone, south eastern Ethiopia | <http://www.academicjournals.org/JAHR> | Adult population |
| 7. | Bukenya, 2019 | What causes non-adherence among some individuals on long term antiretroviral therapy? Experiences of individuals with poor viral suppression in Uganda | <https://doi.org/10.1186/s12981-018-0214-y> | Qualitative study |
| 8. | Buma, 2015 | The Influence of HIV-Status Disclosure on Adherence, Immunological and Virological Outcomes among HIV-Infected Patients Started on Antiretroviral Therapy in Dar-esSalaam, Tanzania | http://dx.doi. org/10.16966/2380-5536.111 | Adult population |
| 9. | Coetzee, 2015 | Barriers and facilitators to paediatric adherence to antiretroviral therapy in rural South Africa: a multi-stakeholder perspective | <http://dx.doi.org/10.1080/09540121.2014.967658> | Qualitative study |
| 10. | Demas, 2022 | Adherence to Antiretroviral Treatment and Associated Factors among Seropositive People Received Treatment in Jimma Town Public Health Facilities, Ethiopia | <https://www.ncbi.nlm.nih.gov/pmc/articles/PMC9393663/> | Adult population |
| 11. | Desta et.al, 2020 | Level of Adherence and Associated Factors Among HIV-Infected Patients on Antiretroviral Therapy in Northern Ethiopia: Retrospective Analysis | <https://doi.org/10.2147/PPA.S268395> | Adolescent and adult population |
|  | Dwoki, 2016 | Factors Affecting Adherence to Anti-retroviral Therapy at Kampala International University Teaching Hospital, Bushenyi District, Uganda | <https://pubs.sciepub.com/ajmsm/4/1/4/index.html> | Done on the entire population |
|  | Ejigu, 2020 | Adherence to Combined Antiretroviral Therapy and Associated Factors Among People Living with HIV Attending Nekemte Specialized Hospital, Oromia, Ethiopia | <https://doi.org/10.2147/HIV.S239995> | Adult population |
|  | Eyassu,2016 | Adherence to antiretroviral therapy among HIV and AIDS patients at the Kwa-Thema clinic in Gauteng Province, South Africa | <https://pubmed.ncbi.nlm.nih.gov/27380858> | Adult population |
|  | Filimão, 2019 | Individual factors associated with time to nonadherence to ART pick-up within HIV care and treatment services in three health facilities of Zambe´zia Province, Mozambique | <https://doi.org/10.1371/journal.pone.0213804> | Adult population |
|  | Gachara | Evaluating Adherence to Antiretroviral Therapy Using Pharmacy Refill Records in a Rural Treatment Site in South Africa | <https://doi.org/10.1155/2017/5456219> | Adult population |
|  | Gichane et.al, 2018 | Caregiver Role in HIV Medication Adherence Among HIVInfected Orphans in Tanzania | <https://www.ncbi.nlm.nih.gov/pmc/articles/PMC5895501/pdf/nihms919725.pdf> | Target population were 13-24 years and qualitative study |
|  | Gonah, 2016 | Determinants of Optimal Adherence to Antiretroviral Therapy among People Living With HIV/AIDS Registered for Antiretroviral Therapy in Zimbabwe | <https://www.ajol.info/index.php/mjz/article/view/152144> | Adult population |
|  | Haile, 2023 | Level of Antiretroviral Therapy Adherence and Associated Factors Among People Living with HIV in the Context of Early Antiretroviral Therapy Initiation in Wolaita Sodo Town, Ethiopia | <https://www.liebertpub.com/doi/10.1089/AID.2022.0100> | Adult population |
|  | Haile Giorgis, 2015 | Adherence to Antiretroviral Therapy and Associated Factors among Patients Living with HIV/AIDS at Dil Chora Referral Hospital in Dire Dawa, Eastern Ethiopia | <http://hdl.handle.net/123456789/7016> | Adult population |
|  | Himaubi, 2017 | Motivating factors to antiretroviral treatment adherence by people living with hiv in ndola district | <http://dspace.unza.zm/handle/123456789/5709> | Adult population |
|  | Kabogo, 2018 | Evidence of reduced treatment adherence among HIV infected paediatric and adolescent populations in Nairobi at the onset of the UNAIDS Universal Test and Treat Program | <https://doi.org/10.1186/s13104-018-3205-0> | Target population  children and adolescents (1-18 years) |
|  | Kahema, 2018 | Factors influencing adherence to antiretroviral therapy among HIV infected patients in Nyamagana-Mwanza, Northern Tanzania | <https://www.clinmedjournals.org/articles/iamm/international-archives-of-medical-microbiology-iamm-1-002.php?jid=iamm> | Adult population |
|  | Kairania, 2022 | HIV status disclosure and antiretroviral therapy adherence among children in Masaka region, Uganda | <https://pubmed.ncbi.nlm.nih.gov/36111384/> | Target population children aged 12-17 years |
|  | Ketema, 2015 | Assessment of adherence to highly active antiretroviral therapy and associated factors among people living with HIV at Debrebrihan Referral Hospital and Health Center, Northeast Ethiopia | <https://doi.org/10.2147/HIV.S79328> | Adult population |
|  | Kilapilo, 2022 | Adherence to Antiretroviral Therapy and Associated Factors Among People Living With HIV Following the Introduction of Dolutegravir Based Regimens in Dar es Salaam, Tanzania | <https://pubmed.ncbi.nlm.nih.gov/35243923/> | Adult population |
|  | Lain, 2023 | Caregivers’ psychosocial assessment for identifying HIVinfected infants at risk of poor treatment adherence: an exploratory study in southern Mozambique | <https://www.ncbi.nlm.nih.gov/pmc/articles/PMC10071299/pdf/nihms-1883136.pdf> | Qualitative study |
|  | Lithole, 2019 | Factors contributing to antiretroviral treatment non-adherence among caregivers of children under five years of age, vhembe district, South Africa | <https://univendspace.univen.ac.za/bitstream/handle/11602/1654/Dissetation%20-%20Lithole%2C%20t.%20j.-.pdf?sequence=1&isAllowed=n> | Qualitative study |
|  | Manenzhe, 2019 | Adherence to antiretroviral therapy and mental health of HIV diagnosed patients in vhembe district, limpopo province,South Africa | <http://hdl.handle.net/11602/1501> | Adult population |
|  | Martelli, 2019 | Adherence to antiretroviral treatment among children and adolescents in Tanzania: Comparison between pill count and viral load outcomes in a rural context of Mwanza region | <https://doi.org/10.1371/journal.pone.0214014> | Child and adolescents (0-18 years) |
|  | Mengesha, 2022 | Antiretroviral therapy non-adherence among children living with HIV in Dire Dawa, Eastern Ethiopia | <https://doi.org/10.1186/s12887-022-03697-1> | Target population Children aged 6-17 years |
|  | Mitiku 2016, | Factors Associated With Adherence To Antiretroviral Therapy of HIV/AIDS Patients, In North Shoa Zone Hospitals and Health Centers, Ethiopia | <https://www.researchgate.net/publication/328531557> | Adult population |
|  | Momanyi, 2017 | Factors associated with the prevalence of non-adherence to antiretroviral therapy among HIV positive patients in kibra slums, Nairobi, Kenya | <http://ir.jkuat.ac.ke/bitstream/handle/123456789/3070/Momanyi%2C%2C%20%20Zipporah%20Kemunto.%20MSc.%20%20Epidemiology%2C%202017.pdf?sequence=1&isAllowed=y> | Adult population |
|  | MOSHA, 2019 | Factors influencing Adherence to Antiretroviral Therapy among People Living with HIV in an Urban and Rural Setting, Tanzania | <file:///C:/Users/Gebrye/Downloads/ajol-filejournals_54_articles_186039_submission_proof_186039-637-473021-1-10-20190429.pdf> | Adult population |
|  | Mubekapi, 2015 | A formative and outcome evaluation of a community based antiretroviral therapy adherence programme: the case of sizophila, cape town, south Africa | <https://www.semanticscholar.org/paper/A-formative-and-outcome-evaluation-of-a-community-%3A> Mubekapi/9a97730a9e4b0944333badbdcb38dbe38778a824 | Target population all newly diagnosed with HIV |
|  | Mugoh, 2016 | Patient factors influencing adherence to ART treatment among HIV/AIDS patients in embu teaching and referral hospital comprehensive care clinic | <http://www.sciencepublishinggroup.com/j/sjph> | Adult population |
|  | MUGOH, 2017 | Factors Influencing Adherence To Antiretroviral Therapy In Embu Teaching And Referral Hospital, Embu County, Kenya | <http://irlibrary.ku.ac.ke/handle/123456789/17965> | Adult population |
|  | Mugoh,2016 | Therapy and Health System-Related Factors Influencing Adherence to ART Treatment among HIV/AIDS Patients in Embu Teaching and Referral Hospital Comprehensive Care Clinic | <http://www.sciencepublishinggroup.com/j/ajns> | Target population unclear and poor quality |
|  | Mukui et.al, 2016 | Rates and Predictors of Non-Adherence to Antiretroviral Therapy among HIV-Positive Individuals in Kenya | <https://journals.plos.org/plosone/article/file?id=10.1371/journal.pone.0167465&type=printable> | Adult population |
|  | Mutagonda,2022 | Adherence, Effectiveness and Safety of Dolutegravir Based Antiretroviral Regimens among HIV Infected Children and Adolescents in Tanzania | <https://www.ncbi.nlm.nih.gov/pmc/articles/PMC9257168/pdf/10.1177_23259582221109613.pdf> | HIV-infected children and adolescents up to 19 years were enrolled. |
|  | Nadio, 2020 | Factors Associated with Non-adherence to AntiRetroviral Therapy among Clients in Lodwar County and Referral Hospital, Turkana County, Kenya | <http://www.sdiarticle4.com/review-history/61272> | Done on all HIV infected clients |
|  | Negesa, 2017 | Adherence to antiretroviral therapy and factors affecting among people living with HIV/AIDS and taking antiretroviral therapy, Dire Dawa Town, Eastern Ethiopia | <http://infectious-diseases-and-treatment.imedpub.com/archive.php> | Adult population |
|  | Nyaboke, 2023 | Factors associated with adherence and viral suppression among patients on secondline antiretroviral therapy in an urban HIV program in Kenya | <https://www.ncbi.nlm.nih.gov/pmc/articles/PMC10052608/pdf/10.1177_20503121231162354.pdf> | Adult population |
|  | Nyogea, 2015 | Determinants of antiretroviral adherence among HIV positive children and teenagers in rural Tanzania | <https://pubmed.ncbi.nlm.nih.gov/25637106/> | Children and teenagers aged 2-19 years |
|  | Ochieng, 2021 | Factors Associated with Adherence to Antiretroviral Drugs among HIV Positive Patients Attending Selected Comprehensive Care Centers in SemiUrban, Kenya | <https://doi.org/10.52403/ijhsr.20211219> | Adult population |
|  | Olds, 2015 | Assessment of HIV antiretroviral therapy adherence by measuring drug concentrations in hair among children in rural Uganda | <https://www.ncbi.nlm.nih.gov/pmc/articles/PMC4305465/pdf/nihms644221.pdf> | Outcome of interest not available and poor quality |
|  | Olds, 2015 | Explaining Antiretroviral Therapy Adherence Success Among HIV-Infected Children in Rural Uganda | <https://www.ncbi.nlm.nih.gov/pmc/articles/PMC4393764/pdf/nihms636127.pdf> | Qualitative study |
|  | Petse, 2018 | Antiretroviral treatment adherence among patients in selected health facilities in East London, South Africa | <https://www.researchgate.net/publication/327362976> | Adult population |
|  | Sangeda, 2018 | Predictors of non-adherence to antiretroviral therapy at an urban HIV care and treatment center in Tanzania | <https://doi.org/10.2147/DHPS.S143178> | Adult population |
|  | Tegegne, 2022 | Poor adherence to highly active antiretroviral therapy and associated factors among people living with HIV in Eastern Ethiopia | <https://www.ncbi.nlm.nih.gov/pmc/articles/PMC9234842/pdf/10.1177_20503121221104429.pdf> | Adult population |
|  | Tegegne, 2016 | Adherence and Contributing Factors among HIV Infected Children in Adama Hospital Medical College, Ethiopia | <https://www.researchgate.net/publication/301315836> | HIV-infected children aged 6 months to 18 years |
|  | Teshome, 2015 | Who takes the medicine? Adherence to antiretroviral therapy in southern Ethiopia | <https://doi.org/10.2147/PPA.S90816> | Adult population |
|  | Thoren, 2015 | High adherence to antiretroviral treatment despite frequent adverse effects among people living with HIV in Dodoma, Tanzania | <https://gupea.ub.gu.se/bitstream/handle/2077/39189/gupea_2077_39189_1.pdf?sequence=1> | Adult population |
|  | Vreeman, 2018 | Adherence to antiretroviral therapy in a clinical cohort of HIV-infected children in East Africa | https://doi.org/10.1371/ journal.pone.0191848 | Outcome of interest not available |
|  | Yadeta, 2015 | Predictors of Art Adherence among People Living with Human  Immune Virus Attending Treatment at Hospitals in West Shewa Zone, Oromia Region, Ethiopia, | <https://www.iiste.org/Journals/index.php/JHMN/article/view/32667/0> | The source population is all people living with HIV (PLHIV) |
|  | Sangeda, 2015 | Predictors of non-adherence to antiretroviral therapy at an urban HIV care and treatment center in Tanzania | <https://www.tandfonline.com/doi/full/10.2147/DHPS.S143178> | Adult population |

S4 Table: Reasons for excluded primary studies

| N^o^ | Author, year | Title | Reference DOI | Main reason for exclusion |
| --- | --- | --- | --- | --- |
| 57 | MAL-ED Network Investigators,2017 | Childhood stunting in relation to the pre- and postnatal environment during the first 2 years of life: The MAL-ED longitudinal birth cohort study | 10.1371/journal.pmed.1002408 | The tittle was no similar to ours tittle |
| 58 | Aamodt, 2015 | Genetic relatedness and risk factor analysis of ampicillin-resistant and high-level gentamicin-resistant enterococci causing bloodstream infections in Tanzanian children | 10.1186/s12879-015-0845-8 |  |
| 59 | Abade, 2018 | Use of TaqMan Array Cards to Screen Outbreak Specimens for Causes of Febrile Illness in Tanzania | 10.4269/ajtmh.18-0071 | Excluded by the title |
| 60 | Abate, 2019 | Chronic Malnutrition Among Under Five Children of Ethiopia May Not Be Economic. A Systematic Review and Meta-Analysis | 10.4314/ejhs.v29i2.14 | Excluded by the title |
| 61 | Abaye, 2019 | Prevalence and risk factors of pneumococcal nasopharyngeal carriage in healthy children attending kindergarten, in district of Arsi Zone, South East, Ethiopia | 10.1186/s13104-019-4283-3 | Excluded by the title |
| 62 | Abbawa, 2015 | Fertility desire and associated factors among clients on highly active antiretroviral treatment at finoteselam hospital Northwest Ethiopia: A cross sectional study | 10.1186/s12978-015-0063-2 | Excluded by the title |
| 63 | Abdella, 2022 | Tuberculous Meningitis in Children: Treatment Outcomes at Discharge and Its Associated Factors in Eastern Ethiopia: A Five Years Retrospective Study | 10.2147/IDR.S36575 | Excluded by the title |
| 64 | Karim,2015 | HIV-positive status disclosure in patients in care in rural South Africa: implications for scaling up treatment and prevention interventions |  | Excluded by the title |
| 65 | ABDULRAHMAN, 2015 | IMPACT OF REMINDER MODULE ON ADHERENCE AND TREATMENT OUTCOMES AMONG HIV-POSITIVE PATIENTS ON ANTIRETROVIRAL THERAPY IN HOSPITAL SUNGAI BULOH, MALAYSIA |  |  |
| 66 | Abdulrahman, 2019 | HIV Treatment Adherence-A Shared Burden for Patients, Health-Care Providers, and Other Stakeholders | 10.24875/AIDSRev.19000037. | Excluded by the abstract |
| 67 | Abdulrahman, 2019 | Conceptual Framework for Investigating and Influencing Adherence Behavior among HIV-Positive Populations: An Applied Social Cognition Model | [10.24875/AIDSRev.19000069](http://dx.doi.org/10.24875/AIDSRev.19000069) | Excluded by the abstract |
| 68 | Abebaw, 2020 | Prevalence of intestinal parasites and associated factors among children from child centres in Bahir Dar city, northwest Ethiopia | 10.1177/0049475520920161 | Excluded by the title |
| 69 | ABEBE, 2019 | Household food security and associated factors among Adult people living with HIV/AIDS attending ART clinic in Hospitals of Hawassa Town, Southern Ethiopia 2017 |  | Excluded by the title |
| 70 | Abera, 2017 | Prevalence of malnutrition and associated factors in children aged 6-59 months among rural dwellers of damot gale district, south Ethiopia: community based cross sectional study | 10.1186/s12939-017-0608-9 | Excluded by the title |
| 71 | Abera, 2018 | Magnitude of stunting and its determinants in children aged 6-59 months among rural residents of Damot Gale district; southern Ethiopia | 10.1186/s13104-018-3666-1 | Excluded by the title |
| 72 | Abessa, 2017 | Developmental performance of hospitalized severely acutely malnourished under-six children in low- income setting | 10.1186/s12887-017-0950-5 | Excluded by the title |
| 73 | Abiodun,  2022 | Socioeconomic, clinical, and behavioral characteristics of adolescents living with HIV in Southwest Nigeria: implication for preparedness for transition to adult care | 10.1080/09540121.2021.1906402 | Excluded by the title |
| 74 | Abiola, 2015 | Baseline adherence, socio-demographic, clinical, immunological, virological and anthropometric characteristics of 242 HIV positive patients on ART in Malaysia |  | Excluded by the title |
| 75 | Abtew, 2016 | Knowledge of pregnant women on mother-to-child transmission of HIV, its prevention, and associated factors in Assosa town, northwest Ethiopia | 10.2147/HIV.S100301 | Excluded by the title |
| 76 | Abuga, 2022 | Neurological impairment and disability in children in rural Kenya | 10.1111/dmcn.15059 | Excluded by the title |
| 77 | Abuga, 2019 | Premature mortality in children aged 6-9 years with neurological impairments in rural Kenya: a cohort study | 10.1016/s2214-109x(19)30425-5 | Excluded by the title |
| 78 | Abuga, 2020 | How Severe Anaemia Might Influence the Risk of Invasive Bacterial Infections in African Children | 10.3390/ijms21186976 | Excluded by the title |
| 79 | Acácio, 2019 | Risk factors for death among children 0-59 months of age with moderate-to-severe diarrhea in Manhiça district, southern Mozambique | 10.1186/s12879-019-3948-9 | Excluded by the title |
| 80 | Acácio, 2021 | Rotavirus disease burden pre-vaccine introduction in young children in Rural Southern Mozambique, an area of high HIV prevalence | 10.1371/journal.pone.0249714 | Excluded by the title |
| 81 | Acácio, 2015 | Under treatment of pneumonia among children under 5 years of age in a malaria-endemic area: population-based surveillance study conducted in Manhica district- rural, Mozambique | 10.1016/j.ijid.2015.05.010 | Excluded by the title |
| 82 | Acharya, 2021 | Retrospective cohort analysis of survival of children living with HIV/AIDS in Mumbai, India | 10.1136/bmjopen-2021-050534 | Excluded by the title |
| 83 | ADAGI, 2023 | Factors Influencing Virological Suppression Among Hiv Infected Sex Workers and General Populations in Homa Bay County, Kenya |  | Excluded by the title |
| 84 | Adane, 2023 | Risk Factors of Cooking-related Burn Injury Among Under-Four Children in Northwest Ethiopia: A Community-Based Cross-Sectional Study |  | Excluded by the title |
| 85 | Adane, 2023 | Prevalence and risk factors of cooking-related burn injury among under-five-years old children in a resource-limited setting: a community-based cross-sectional study in Northwest Ethiopia | 10.1080/17457300.2022.2125534 | Excluded by the title |
| 86 | Adane, 2020 | Prevalence and risk factors of acute lower respiratory infection among children living in biomass fuel using households: a community-based cross-sectional study in Northwest Ethiopia | 10.1186/s12889-020-08515-w | Excluded by the title |
| 87 | Addo, 2022 | Living with tuberculosis: a qualitative study of patients’ experiences with disease and treatment |  | Excluded by the title |
| 88 | Adejumo, 2015 | Contemporary issues on the epidemiology and antiretroviral adherence of HIV-infected adolescents in sub-Saharan Africa: A narrative review | 10.7448/IAS.18.1.20049 | Excluded by the title |
| 89 | Adelekan, 2019 | Social barriers in accessing care by clients who returned to HIV care after transient loss to follow-up |  | Excluded by the title |
| 90 | Adella, 2023 | Human immunodeficiency virus positive status disclosure among children in northwest Ethiopia: a cross-sectional study | 10.4314/ahs.v23i1.20 | Excluded by the title |
| 91 | Adeniran, 2021 | Determinants of Adherence among Patients on Highly Active Anti-Retroviral Therapy in Lagos State, Nigeria |  | Excluded by the title |
| 92 | Adenomon, 2019 | Factors That Enhanced Prevention of Mother-to-Child Transmission of HIV in Nasarawa State of Nigeria Using Logistic, Poisson and Negative Binomial Regression Models |  | Excluded by the title |
| 93 | Adeoti, 2019 | Survey of antiretroviral therapy adherence and predictors of poor adherence among HIV patients in a tertiary institution in Nigeria |  | Excluded by the title |
| 94 | Adetifa, 2018 | Coverage and timeliness of vaccination and the validity of routine estimates: Insights from a vaccine registry in Kenya | 10.1016/j.vaccine.2018.11.005 | Excluded by the title |
| 95 | Adeyemi, 2019 | Joint spatial mapping of childhood anemia and malnutrition in sub-Saharan Africa: a cross-sectional study of small-scale geographical disparities | 10.4314/ahs.v19i3.45 | Excluded by the title |
| 96 | Adimasu, 2020 | Recovery time from severe acute malnutrition and associated factors among under-5 children in Yekatit 12 Hospital, Addis Ababa, Ethiopia: a retrospective cohort study | 10.4178/epih.e2020003 | Excluded by the title |
| 97 | Adinan, 2020 | Factors Associated with Testing and Prompt Use of Recommended Antimalarials following Malaria Diagnosis: A Secondary Analysis of 2011-12 Tanzania HIV and Malaria Indicator Survey Data |  | Excluded by the title |
| 98 | Aduloju, 2020 | Medication adherence in HIV-positive pregnant women on antiretroviral therapy attending antenatal clinics in Ado metropolis, south-west Nigeria: A multicentre study |  | Excluded by the title |
| 99 | Agbor | Preferred Audience of HIV Status Disclosure and Reasons among HIV Positive Individuals in Cross River State |  | Excluded by the title |
| 100 | Agot, 2021 | Risk factors for major external structural birth defects among children in Kiambu County, Kenya: a case-control study | 10.12688/f1000research.50738.2 | Excluded by the title |
| 101 | Aheto, 2020 | Geostatistical analysis and mapping: social and environmental determinants of under-five child mortality, evidence from the 2014 Ghana demographic and health survey | 10.1186/s12889-020-09534-3 | Excluded by the title |
| 102 | Ahinkorah, 2023 | Physical violence during pregnancy in sub-Saharan Africa: why it matters and who are most susceptible? | 10.1136/bmjopen-2021-059236 | Excluded by the title |
| 103 | Alinaghi, 2016 | Adherence to antiretroviral therapy and tuberculosis treatment in a prison of Tehran, Iran |  | Excluded by the title |
| 104 | Ahmadi, 2018 | Determinants of child anthropometric indicators in Ethiopia | 10.1186/s12889-018-5541-3 | Excluded by the title |
| 105 | Ahmed, 2021 | Mapping Geographical Differences and Examining the Determinants of Childhood Stunting in Ethiopia: A Bayesian Geostatistical Analysis | 10.3390/nu13062104 | Excluded by the title |
| 106 | Ahmed, 2023 | Population-Modifiable Risk Factors Associated With Childhood Stunting in Sub-Saharan Africa | 10.1001/jamanetworkopen.2023.38321 | Excluded by the title |
| 107 | Ahmed, 2020 | Associations between infant and young child feeding practices and acute respiratory infection and diarrhoea in Ethiopia: A propensity score matching approach | 10.1371/journal.pone.0230978 | Excluded by the title |
| 108 | Ahmed, 2023 | Mapping Local Variations and the Determinants of Childhood Stunting in Nigeria | 10.3390/ijerph20043250 | Excluded by the title |
| 109 | Ahmed, 2020 | Protecting children in low-income and middle-income countries from COVID-19 | 10.1136/bmjgh-2020-002844 | Excluded by the title |
| 110 | Akalu, 2024 | Risk factors for diagnosis and treatment delay among patients with multidrug-resistant tuberculosis in Hunan Province, China | 10.1186/s12879-024-09036-2 | Excluded by the title |
| 111 | Akech, 2023 | Tackling post-discharge mortality in children living in LMICs to reduce child deaths | 10.1016/s2352-4642(22)00375-3 | Excluded by the title |
| 112 | Akilimali, 2017 | Disclosure of HIV status and its impact on the loss in the follow-up of HIV-infected patients on potent anti-retroviral therapy programs in a (post-) conflict setting: A retrospective cohort study from Goma, Democratic Republic of Congo |  | Excluded by the title |
| 113 | Akindele, 2015 | The effect of socio-economic status on adherence to anti-retroviral therapy |  | Excluded by the title |
| 114 | Akinloye, 2022 | Validation and Clinical Application of a Liquid Chromatography-Ultraviolet Detection Method to Quantify Dolutegravir in Dried Blood Spots |  | Excluded by the title |
| 115 | ALABI, 2017 | FACTORS INFLUENCING ADHERENCE TO ANTIRETROVIRAL THERAPY AMONG PEOPLE LIVING WITH HIV/AIDS RECEIVING CARE AT ADEOYO MATERNITY TEACHING HOSPITAL, YEMETU, IBADAN, NIGERIA |  | Excluded by the title |
| 116 | Alamirew, 2022 | Prevalence and associated factors of neonatal mortality in Ethiopia | 10.1038/s41598-022-16461-3 | Excluded by the title |
| 117 | Alamneh, 2023 | Determinants of anemia severity levels among children aged 6-59 months in Ethiopia: Multilevel Bayesian statistical approach | 10.1038/s41598-022-20381-7 | Excluded by the title |
| 118 | Alamneh, 2020 | Magnitude and Predictors of Pneumonia among Under-Five Children in Ethiopia: A Systematic Review and Meta-Analysis | 10.1155/2020/1606783 | Excluded by the title |
| 119 | Alaro, | Healthcare Providers in Africa Face HIV and AIDS Challenges in Providing Services to a Key Population: Infant and Young Child Feeding |  | Excluded by the title |
| 120 | Alavian, 2023 | Tenofovir alafenamide plus dolutegravir as a switch strategy in HIV-infected patients: a pilot randomized controlled trial |  | Excluded by the title |
| 121 | Alemayehu, 2020 | Individual and community-level risk factors in under-five children diarrhea among agro-ecological zones in southwestern Ethiopia | 10.1016/j.ijheh.2019.113447 | Excluded by the title |
| 122 | Alemayehu, 2020 | Exploring the association between childhood diarrhea and meteorological factors in Southwestern Ethiopia | 10.1016/j.scitotenv.2020.140189 | Excluded by the title |
| 123 | Alemayehu, 2015 | Prevalence of Active Trachoma and Its Associated Factors among Rural and Urban Children in Dera Woreda, Northwest Ethiopia: A Comparative Cross-Sectional Study | 10.1155/2015/570898 | Excluded by the title |
| 124 | Alemayehu, 2015 | Undernutrition status and associated factors in under-5 children, in Tigray, Northern Ethiopia | 10.1016/j.nut.2015.01.013 | Excluded by the title |
| 125 | Alemayehu, 2019 | Risk factors of acute respiratory infections among under five children attending public hospitals in southern Tigray, Ethiopia, 2016/2017 | 10.1186/s12887-019-1767-1 | Excluded by the title |
| 126 | Alemu, 2016 | chistosoma mansoni and soil-transmitted helminths among preschool-aged children in Chuahit, Dembia district, Northwest Ethiopia: prevalence, intensity of infection and associated risk factors | 10.1186/s12889-016-2864-9 | Excluded by the title |
| 127 | Alemu, 2022 | Survival time and predictors of death among HIV infected under five children after initiation of anti-retroviral therapy in West Amhara Referral Hospitals, Northwest Ethiopia |  | Excluded by the title |
| 128 | Alhassan, 2023 | Predictors of HIV status disclosure among PLHIV in the Volta region of Ghana: the disclosure conundrum and its policy implication |  | Excluded by the title |
| 129 | Alhassan, 2023 | Predictors of HIV status disclosure among people living with HIV (PLHIV) in Ghana: the disclosure conundrum and its policy implications in resource limited settings |  | Excluded by the title |
| 130 | Ali, 2023 | Prevalence and risk factors of childhood diarrhea among wastewater irrigating urban farming households in Addis Ababa | 10.1371/journal.pone.0288425 | Excluded by the title |
| 131 | Ali, 2018 | Adherence to antiretroviral therapy in HIV-positive, male intravenous drug users in Pakistan |  | Excluded by the title |
| 132 | Aliyo, 2022 | Assessment of anemia and associated risk factors among children under-five years old in the West Guji Zone, southern Ethiopia: Hospital-based cross-sectional study | 10.1371/journal.pone.0270853 | Excluded by the title |
| 133 | Ally, 2023 | Determinants of viral load suppression among orphaned and vulnerable children living with HIV on ART in Tanzania | 10.3389/fpubh.2023.1076614 | Excluded by the title |
| 134 | Altamirano, 2023 | Understanding ART adherence among adolescent girls and young women in western Kenya: a cross-sectional study of barriers and facilitators |  | Excluded by the title |
| 135 | Altamirano, 2023 | Understanding ART Adherence among Adolescent Girls and Young Women in Western Kenya: A Cross-Sectional Study of Barriers and Facilitators | 10.3390/ijerph20206922 | Excluded by the title |
| 136 | Altare, 2016 | Child Wasting in Emergency Pockets: A Meta-Analysis of Small-Scale Surveys from Ethiopia | 10.3390/ijerph13020178 | Excluded by the title |
| 137 | Altaseb, 2024 | Prevalence of trachomatous inflammation-follicular and associated factors among children aged 1-9 years in northeastern Ethiopia | 10.1186/s12887-024-04587-4 | Excluded by the title |
| 138 | Altherr, 2019 | Associations between Water, Sanitation and Hygiene (WASH) and trachoma clustering at aggregate spatial scales, Amhara, Ethiopia | 10.1186/s13071-019-3790-3 | Excluded by the title |
| 139 | Alvi, 2019 | World Health Organization dimensions of adherence to antiretroviral therapy: A study at antiretroviral therapy centre, Aligarh |  | Excluded by the title |
| 140 | Amankwah-Poku, 2021 | Disclosure and health-related outcomes among children living with HIV and their caregivers | 10.1186/s12981-021-00337-z | Excluded by the title |
| 141 | Amare, 2019 | Determinants of nutritional status among children under age 5 in Ethiopia: further analysis of the 2016 Ethiopia demographic and health survey | 10.1186/s12992-019-0505-7 | Excluded by the title |
| 142 | Ambarwati,  2021 | The effect of iron deficiency anemia on the child death related to HIV/AIDS infection: meta-analysis |  | Excluded by the title |
| 143 | Andegiorgish, 2017 | Examining the Effect of Geographic Region of Residence on Childhood Malnutrition in Uganda | 10.1186/s12903-017-0465-3 | Excluded by the title |
| 144 | Anjajo, 2023 | Determinants of hypertension among diabetic patients in southern Ethiopia: a case-control study | 10.1186/s12872-023-03245-4 | Excluded by the title |
| 145 | Ansari, | Prevalence and risk factors of Severe Acute Respiratory Syndrome Coronavirus 2 infection in women and children in peri-urban communities in Pakistan: A prospective cohort study | 10.7189/jogh.12.95955 | Excluded by the title |
| 146 | Antelman, 2021 | Balancing HIV testing efficiency with HIV caseidentification among children and adolescents (2-19 years) using an HIV risk screening approach in Tanzania | 10.1371/journal.pone.0251247 | Excluded by the title |
| 147 | Anteneh, 2017 | Prevalence and determinants of acute diarrhea among children younger than five years old in Jabithennan District, Northwest Ethiopia, 2014 | 10.1186/s12889-017-4021-5 | Excluded by the title |
| 148 | Anteneh, 2023 | Validation of risk prediction for outcomes of severe community-acquired pneumonia among under-five children in Amhara region, Northwest Ethiopia | 10.1371/journal.pone.0281209 | Excluded by the title |
| 149 | Aoun, 2015 | Geographical accessibility to healthcare and malnutrition in Rwanda | 10.1016/j.socscimed.2015.02.004 | Excluded by the title |
| 150 | Apondi, | Barriers to ART adherence among school students living with HIV in Kenya |  | Excluded by the title |
| 151 | Aramburo, 2018 | Lactate clearance as a prognostic marker of mortality in severely ill febrile children in East Africa | 10.1186/s12916-018-1014-x | Excluded by the title |
| 152 | Araya, 2018 | The role of counseling on modern contraceptive utilization among HIV positive women: The case of Northwest Ethiopia | 10.1186/s12905-018-0603-3 | Excluded by the title |
| 153 | Ardura-Garcia, 2015 | Implementation and Operational Research: Early Tracing of Children Lost to Follow-Up From Antiretroviral Treatment: True Outcomes and Future Risks | 10.1097/qai.0000000000000772 | Excluded by the title |
| 154 | Argaw, 2019 | Drivers of Under-Five Stunting Trend in 14 Low- and Middle-Income Countries since the Turn of the Millennium: A Multilevel Pooled Analysis of 50 Demographic and Health Surveys | 10.3390/nu11102485 | Excluded by the title |
| 155 | Argaw, 2020 | Sero-prevalence of hepatitis B virus markers and associated factors among children in Hawassa City, southern Ethiopia | 10.1186/s12879-020-05229-7 | Excluded by the title |
| 156 | Arikawa, 2023 | Prevalence and risk factors associated with malaria infection in children under two years of age in southern Togo prior to perennial malaria chemoprevention implementation | 10.1186/s12936-023-04793-y | Excluded by the title |
| 157 | Arisudhana, 2022 | Pengaruh peer leader support terhadap kepatuhan minum obat orang dengan hiv aids |  | Excluded by the title |
| 158 | Arkew, 2024 | Thrombocytopenia and associated factors among pregnant women attending antenatal care at Hiwot Fana Comprehensive Specialized Hospital, Eastern Ethiopia: A cross-sectional study | 10.1097/md.0000000000036866 | Excluded by the title |
| 159 | Arnold, 2015 | Surgical manifestations of gastrointestinal cytomegalovirus infection in children: Clinical audit and literature review | 10.1016/j.jpedsurg.2015.06.018 | Excluded by the title |
| 160 | Asefa, 2020 | Level of good adherence on option b+ pmtctand associated factors among hiv positive pregnant and lactating mothers in public health facilities of ilu abba bor and buno bedele zones, oromia regional state, Southwestern Ethiopia, 2018 | 10.2147/HIV.S283184 | Excluded by the title |
| 161 | Asfaw, 2020 | Determinants of soil-transmitted helminth infections among pre-school-aged children in Gamo Gofa zone, Southern Ethiopia: A case-control study | 10.1371/journal.pone.0243836 | Excluded by the title |
| 162 | Asgedom, 2024 | Levels of stunting associated factors among under-five children in Ethiopia: A multi-level ordinal logistic regression analysis | 10.1371/journal.pone.0296451 | Excluded by the title |
| 163 | Ashaba, 2019 | Development and validation of a 20-item screening scale to detect major depressive disorder among adolescents with HIV in rural Uganda: A mixed-methods study |  | Excluded by the title |
| 164 | Asiki, 2018 | Sociodemographic and behavioural factors associated with body mass index among men and women in Nairobi slums: AWI-Gen Project | 10.1080/16549716.2018.1470738 | Excluded by the title |
| 165 | Asiki, 2016 | The impact of maternal factors on mortality rates among children under the age of five years in a rural Ugandan population between 2002 and 2012 | 10.1111/apa.13252 | Excluded by the title |
| 166 | Asmare, 2022 | Disparities in full immunization coverage among urban and rural children aged 12-23 months in southwest Ethiopia: A comparative cross-sectional study | 10.1080/21645515.2022.2101316 | Excluded by the title |
| 167 | Asrie, 2020 | Magnitude of anemia and associated factors among human immunodeficiency virus infected children on highly active antiretroviral therapy at university of gondar comprehensive and specialized referral hospital northwest Ethiopia | 10.7754/Clin.Lab.2019.190835 | Excluded by the title |
| 168 | Assefa, 2017 | FACTORS ASSOCIATED WITH TREATMENT OUTCOME OF PEDIATRIC CANCERPATIENTS ADMITTED WITH FEBRILE NEUTROPENIA IN TIKURANBESSA SPECIALIZED TEACHING HOSPITAL, ADDIS ABABA, ETHIOPIA |  | Excluded by the title |
| 169 | Astawesegn, 2021 | Prenatal hiv test uptake and its associated factors for prevention of mother to child transmission of hiv in East Africa | 10.3390/ijerph18105289 | Excluded by the title |
| 170 | Atalell, 2018 | Survival and predictors of mortality among children co-infected with tuberculosis and human immunodeficiency virus at University of Gondar Comprehensive Specialized Hospital, Northwest Ethiopia. A retrospective follow-up study |  | Excluded by the title |
| 171 | Atanuriba, 2022 | “Some believe those who say they can cure it” perceived barriers to antiretroviral therapy for children living with HIV/AIDS: Qualitative exploration of caregivers experiences in tamale metropolis | 10.1371/journal.pone.0275529 | Excluded by the title |
| 172 | Atieno | Comarative level of knowledge on adherence to ART among HIV patients attending Jinja regional referral Hospital |  | Excluded by the title |
| 173 | Atieno, 2023 | NEWPORT INTERNATIONAL JOURNAL OF SCIENTIFIC AND EXPERIMENTAL SCIENCES (NIJSES) |  | Excluded by the title |
| 174 | Atim, 2018 | Agony resulting from cultural practices of canine bud extraction among children under five years in selected slums of Makindye: a cross sectional study | 10.1186/s12903-018-0599-y | Excluded by the title |
| 175 | Atkilt, 2017 | Clinical Characteristics of Diabetic Ketoacidosis in Children with Newly Diagnosed Type 1 Diabetes in Addis Ababa, Ethiopia: A Cross-Sectional Study | 10.1371/journal.pone.0169666 | Excluded by the title |
| 176 | Atukunda, 2019 | Factors Associated with Pregnancy Intentions Amongst Postpartum Women Living with HIV in Rural Southwestern Uganda | 10.1007/s10461-018-2317-9 | Excluded by the title |
| 177 | Audrey, 2020 | Assessment of Factors Associated with Suboptimal Adherence of HIV Antiretroviral Therapy in Asia: A Systematic Review |  | Excluded by the title |
| 178 | Augustina, 2019 | Factors associated with non-adherence of HIV/AIDS patients to HAART regimen in a healthcare facility in Ikot Ekpene, Akwa Ibom State, Nigeria |  | Excluded by the title |
| 179 | Aung, 2022 | Stigma determines antiretroviral adherence in adults with HIV in Myanmar |  | Excluded by the title |
| 180 | Avong, 2018 | Integrating community pharmacy into community based anti-retroviral therapy program: A pilot implementation in Abuja, Nigeria |  | Excluded by the title |
| 181 | Awori, 2021 | The Etiology of Pneumonia in HIV-uninfected Children in Kilifi, Kenya: Findings From the Pneumonia Etiology Research for Child Health (PERCH) Study | 10.1097/inf.0000000000002653 | Excluded by the title |
| 182 | Axelsson, 2015 | Antiretroviral therapy adherence strategies used by patients of a large HIV clinic in Lesotho |  | Excluded by the abstract |
| 183 | Ayalew, 2015 | Assessment of Diarrhea and Its Associated Factors in Under-Five Children among Open Defecation and Open Defecation-Free Rural Settings of Dangla District, Northwest Ethiopia | 10.1155/2018/4271915 | Excluded by the title |
| 184 | Ayalew, 2016 | First-line antiretroviral treatment failure and associated factors in HIV patients at the University of Gondar Teaching Hospital, Gondar, Northwest Ethiopia |  | Excluded by the title |
| 185 | Ayele, 2016 | Indirect child mortality estimation technique to identify trends of under-five mortality in Ethiopia | 10.4314/ahs.v16i1.3 | Excluded by the title |
| 186 | Ayele, 2015 | Structured additive regression models with spatial correlation to estimate under-five mortality risk factors in Ethiopia | 10.1186/s12889-015-1602-z | Excluded by the title |
| 187 | Azamar-Alonso, 2022 | HIV in Mexico: A 10-Year Population-Based Analysis to Evaluate Policy Changes in Diagnosis, Treatment, and Early Mortality on PLWH 2008-2017 |  | Excluded by the title |
| 188 | Azamar-Alonso, 2022 | Virologic failure in people living with HIV in 1st line ART: A 10-year Mexican population-based study |  | Excluded by the title |
| 189 | Azia, 2016 | Barriers to adherence to antiretroviral treatment in a regional hospital in Vredenburg, Western Cape, South Africa |  | Excluded by the abstract |
| 190 | Azmach, 2019 | Socioeconomic and demographic statuses as determinants of adherence to antiretroviral treatment in HIV infected patients: a systematic review of the literature |  | Excluded by the title |
| 191 | Babb, 2018 | Evaluation of the effectiveness of a latrine intervention in the reduction of childhood diarrhoeal health in Nyando District, Kisumu County, Kenya | 10.1017/s0950268818000924 | Excluded by the title |
| 192 | Bado, 2016 | Trends and risk factors for childhood diarrhea in sub-Saharan countries (1990-2013): assessing the neighborhood inequalities | 10.3402/gha.v9.30166 | Excluded by the title |
| 193 | Bagoloire, 2023 | Prevalence and Factors Associated with Renal Disease Among HIV Infected Children on Antiretroviral Therapy Attending Joint Clinical Research Center, Lubowa, Kampala | 10.1101/2023.02.09.23285685 | Excluded by the title |
| 194 | BAKEIHA, 2015 | ORGANISATION FACTORS AND ADHERENCE TO ANTI-RETROVIRAL THERAPY AMONG HIV/AIDS PATIENTS AT KISENYI HEALTH CENTRE, KAMPALA CAPITAL CITY AUTHORITY |  | Excluded by the title |
| 195 | Baker, 2016 | Sanitation and Hygiene-Specific Risk Factors for Moderate-to-Severe Diarrhea in Young Children in the Global Enteric Multicenter Study, 2007-2011: Case-Control Study | 10.1371/journal.pmed.1002010 | Excluded by the title |
| 196 | Balasubramani, 2022 | Spatial epidemiology of acute respiratory infections in children under 5 years and associated risk factors in India: District-level analysis of health, household, and environmental datasets | 10.3389/fpubh.2022.906248 | Excluded by the title |
| 197 | Ballot, 2016 | Retrospective cross-sectional review of survival rates in critically ill children admitted to a combined paediatric/neonatal intensive care unit in Johannesburg, South Africa, 2013-2015 | 10.1136/bmjopen-2015-010850 | Excluded by the title |
| 198 | Bam, 2015 | Strengthening adherence to Anti Retroviral Therapy (ART) monitoring and support: operation research to identify barriers and facilitators in Nepal |  | Excluded by the title |
| 199 | Yathiraj, 2016 | Factors influencing adherence to antiretroviral therapy among people living with HIV in Coastal South India |  | Excluded by the title |
| 200 | Banati, 2021 | Addressing the Mental and Emotional Health Impacts of COVID-19 on Children and Adolescents: Lessons From HIV/AIDS | 10.3389/fpsyt.2021.589827 | Excluded by the title |
| 201 | Barbosa, 2016 | Relationship between physical activity, physical fitness and multiple metabolic risk in youths from Muzambinho's study | 10.1080/17461391.2015.1088576 | Excluded by the title |
|  | Barchi, 2019 | Adherence to screening appointments in a cervical cancer clinic serving HIV-positive women in Botswana |  | Excluded by the abstract |
| 202 | Barffour, 2017 | High Iron Stores in the Low Malaria Season Increase Malaria Risk in the High Transmission Season in a Prospective Cohort of Rural Zambian Children | 10.3945/jn.117.250381 | Excluded by the title |
| 203 | Barkish, 2019 | Experiences of patients with primary HIV diagnosis in Kermanshah-Iran regarding the nature of HIV/AIDS: A qualitative study |  | Excluded by the title |
| 204 | Barnett, 2018 | Maltreatment in childhood and intimate partner violence: A latent class growth analysis in a South African pregnancy cohort | 10.1016/j.chiabu.2018.08.020 | Excluded by the title |
| 205 | Barrington, 2020 | Strengthening the “Reach-Test-Treat-Retain” cascade for men who have sex with men in Guatemala: A pilot intervention. EIR-HIV KPIS |  | Excluded by the title |
| 206 | Basha, 2020 | Prevalence and risk factors of active trachoma among primary school children of Amhara Region, Northwest Ethiopia | 10.4103/ijo.IJO_143_19 | Excluded by the title |
| 207 | Basikoro, 2019 | Pathologies of patriarchy: Death, suffering, care, and coping in the gendered gaps of HIV/AIDS interventions in Nigeria |  | Excluded by the title |
| 208 | Bassett, 2017 | Barriers to care and 1-year mortality among newly diagnosed HIV-infected people in Durban, South Africa |  | Excluded by the title |
| 209 | Bateganya, 2015 | Impact of support groups for people living with HIV on clinical outcomes: a systematic review of the literature |  | Excluded by the title |
| 210 | Bateman, 2015 | The risky lives of South Africa's children: Why so many die or are traumatized | 10.7196/samj.9462 | Excluded by the title |
| 211 | Batte, 2022 | Acute kidney injury in hospitalized children with sickle cell anemia | 10.1186/s12882-022-02731-9 | Excluded by the title |
| 212 | Bauhofer, 2021 | Intestinal protozoa in hospitalized under-five children with diarrhoea in Nampula - a cross-sectional analysis in a low-income setting in northern Mozambique | 10.1186/s12879-021-05881-7 | Excluded by the title |
| 213 | Bauhofer, 2023 | Examining comorbidities in children with diarrhea across four provinces of Mozambique: A cross-sectional study (2015 to 2019) | 10.1371/journal.pone.0292093 | Excluded by the title |
| 214 | Bauleth, 2020 | Epidemiology and factors associated with diarrhoea among children under five years of age in the Engela District in the Ohangwena Region, Namibia | 10.4102/phcfm.v12i1.2361 | Excluded by the title |
| 215 | Bauserman, | Maternal mortality in six low and lower-middle income countries from 2010 to 2018: risk factors and trends | 10.1186/s12978-020-00990-z | Excluded by the title |
| 216 | Bawn, 2022 | eHealth for family planning in Botswana: acceptability and feasibility |  | Excluded by the title |
| 217 | Baye, 2020 | Evaluation of Linear Growth at Higher Altitudes | 10.1001/jamapediatrics.2020.2386 | Excluded by the title |
| 218 | Baye, 2020 | Socio-Economic Inequalities in Child Stunting Reduction in Sub-Saharan Africa | 10.3390/nu12010253 | Excluded by the title |
| 219 | Bayleyegn, | Magnitude and associated factors of peripheral cytopenia among HIV-infected children attending at University of Gondar Specialized Referral Hospital, Northwest Ethiopia | 10.1371/journal.pone.0247878 | Excluded by the title |
| 220 | Bayleyegn, 2021 | Prevalence of intestinal parasitic infection and associated factors among HAART initiated children attending at university of gondar comprehensive specialized hospital, northwest Ethiopia | 10.2147/HIV.S287659 | Excluded by the title |
| 221 | Becker, 2020 | Individual, household, and community level barriers to ART adherence among women in rural Eswatini |  | Excluded by the title |
| 222 | Becker, 2021 | A quantitative analysis of food insecurity and other barriers associated with ART nonadherence among women in rural communities of Eswatini |  | Excluded by the title |
| 223 | Beckmann, 2021 | Prevalence of Stunting and Relationship between Stunting and Associated Risk Factors with Academic Achievement and Cognitive Function: A Cross-Sectional Study with South African Primary School Children | 10.3390/ijerph18084218 | Excluded by the title |
| 224 | Bedecha, 2023 | Dual contraception method utilization and associated factors among women on antiretroviral therapy in public facilities of Bishoftu town, Oromia, Ethiopia | 10.1371/journal.pone.0280447 | Excluded by the title |
| 225 | Beichler, 2023 | Integrated Care as a Model for Interprofessional Disease Management and the Benefits for People Living with HIV/AIDS |  | Excluded by the title |
| 226 | Bekele, 2021 | Modeling non-Gaussian data analysis on determinants of underweight among under five children in rural Ethiopia: Ethiopian demographic and health survey 2016 evidences | 10.1371/journal.pone.0251239 | Excluded by the title |
| 227 | Belachew, 2020 | Under-five anemia and its associated factors with dietary diversity, food security, stunted, and deworming in Ethiopia: systematic review and meta-analysis | 10.1186/s13643-020-01289-7 | Excluded by the title |
| 228 | Belachew, 2020 | Prevalence of vertical HIV infection and its risk factors among HIV exposed infants in East Africa: a systematic review and meta-analysis | 10.1186/s41182-020-00273-0 | Excluded by the title |
| 229 | Belay, 2023 | Incidence and pattern of childhood cancer in Addis Ababa, Ethiopia (2012-2017) | 10.1186/s12885-023-11765-7 | Excluded by the title |
| 230 | Belay, 2023 | Spatial distribution and associated factors of severe malnutrition among under-five children in Ethiopia: further analysis of 2019 mini EDHS | 10.1186/s12889-023-15639-2 | Excluded by the title |
| 231 | Beletew, 2020 | Determinants of Anemia among HIV-Positive Children on Highly Active Antiretroviral Therapy Attending Hospitals of North Wollo Zone, Amhara Region, Ethiopia, 2019: A Case-Control Study | 10.1155/2020/3720572 | Excluded by the title |
| 232 | Bénet, 2017 | Severity of Pneumonia in Under 5-Year-Old Children from Developing Countries: A Multicenter, Prospective, Observational Study | 10.4269/ajtmh.16-0733 | Excluded by the title |
| 233 | Benning, 2020 | Examining adherence barriers among women with HIV to tailor outreach for long-acting injectable antiretroviral therapy |  | Excluded by the title |
| 234 | Benoni, 2023 | SARS-CoV-2 seroprevalence and associated factors, based on HIV serostatus, in young people in Sofala province, Mozambique | 10.1186/s12879-023-08808-6 | Excluded by the title |
| 235 | Bergkvist, 2021 | Nutritional status and outcome of surgery: A prospective observational cohort study of children at a tertiary surgical hospital in Harare, Zimbabwe | 10.1016/j.jpedsurg.2020.09.020 | Excluded by the title |
| 236 | Berhe, 2019 | Prevalence and associated factors of zinc deficiency among pregnant women and children in Ethiopia: a systematic review and meta-analysis | 10.1186/s12889-019-7979-3 | Excluded by the title |
| 238 | Berhe, 2019 | Risk factors of stunting (chronic undernutrition) of children aged 6 to 24 months in Mekelle City, Tigray Region, North Ethiopia: An unmatched case-control study | 10.1371/journal.pone.0217736 | Excluded by the title |
| 239 | Bernard, 201 | Prevalence and factors associated with depression in people living with HIV in sub-Saharan Africa: a systematic review and meta-analysis |  | Excluded by the title |
| 240 | Bero, 2016 | Prevalence of and Risk Factors for Trachoma in Oromia Regional State of Ethiopia: Results of 79 Population-Based Prevalence Surveys Conducted with the Global Trachoma Mapping Project | 10.1080/09286586.2016.1243717 | Excluded by the title |
| 241 | Besa, 2015 | Prevalence of pyschiatric disorders in HIV positive patients at Chilenje Clinic in Lusaka Zambia |  | Excluded by the title |
| 242 | Beshir, 2019 | Incidence and predictors of tuberculosis among HIV-positive children at Adama Referral Hospital and Medical College, Oromia, Ethiopia: a retrospective follow-up study | 10.4178/epih.e2019028 | Excluded by the title |
| 243 | Bessong, 2021 | Potential challenges to sustained viral load suppression in the HIV treatment programme in South Africa: a narrative overview |  | Excluded by the title |
| 244 | Blessy, 2016 | Effect of micro and macro nutritient supplementation on disease outcome in adolescents with HIV on HAART: A Randomised Double-blinded clinical trial |  | Excluded by the title |
| 245 | Boivin, 2016 | Malaria illness mediated by anaemia lessens cognitive development in younger Ugandan children | 10.1186/s12936-016-1266-x | Excluded by the title |
| 246 | Bonawitz, 2016 | Identifying Gaps in Prevention of Mother to Child Transmission of HIV: A Case Series of HIV-positive Infants in Zambia | 10.1097/inf.0000000000001155 | Excluded by the title |
| 247 | Buys, 2016 | Klebsiella pneumoniae bloodstream infections at a South African children's hospital 2006-2011, a cross-sectional study | 10.1186/s12879-016-1919-y | Excluded by the title |
| 248 | Bwogi, 2016 | The epidemiology of rotavirus disease in under-five-year-old children hospitalized with acute diarrhea in central Uganda, 2012-2013 | 10.1007/s00705-015-2742-2 | Excluded by the title |
| 249 | Caddick, 2016 | vesting in mental health in low-income countries |  | Excluded by the title |
| 250 | Chelidze, 2016 | Predictors of Mortality Among Pediatric Burn Patients in East Africa | 10.1097/bcr.0000000000000286 | Excluded by the title |
| 251 | Chirwa, 2016 | Factors Affecting Antiretroviral Drug Adherence among HIV Adult Patients attending HIV Clinin at the University Teaching Hospital in Lusaka |  | Excluded by the abstract |
| 252 | Cluver, 2016 | Achieving equity in HIV-treatment outcomes: can social protection improve adolescent ART-adherence in South Africa? | 10.1080/09540121.2016.1179008 | Excluded by the title |
| 253 | Conroy, 2016 | Methemoglobin and nitric oxide therapy in Ugandan children hospitalized for febrile illness: results from a prospective cohort study and randomized double-blind placebo-controlled trial | 10.1186/s12887-016-0719-2 | Excluded by the title |
| 254 | Costenaro, 2016 | Implementation of the WHO 2011 Recommendations for Isoniazid Preventive Therapy (IPT) in Children Living with HIV/AIDS: A Ugandan Experience | 10.1097/QAI.0000000000000806 | Excluded by the title |
| 255 | Crook, 2016 | Tuberculosis incidence is high in HIV-infected African children but is reduced by co-trimoxazole and time on antiretroviral therapy | 10.1186/s12916-016-0593-7 | Excluded by the title |
| 256 | Das, 2016 | Abundance of psychiatric morbidity in perinatally HIV infected children and adolescents with comparison to their HIV negative sibling |  | Excluded by the title |
| 257 | Dedefo, 2016 | Small area clustering of under-five children's mortality and associated factors using geo-additive Bayesian discrete-time survival model in Kersa HDSS, Ethiopia |  | Excluded by the title |
| 258 | Deresse, 2016 | Tuberculosis among Ethiopian-born Georgia residents: An ethnographic approach to understand the sociocultural aspects of tuberculosis |  | Excluded by the title |
| 259 | Hudelson, 2015 | Factors associated with adherence to antiretroviral therapy among adolescents living with HIV/AIDS in low-and middle-income countries: a systematic review |  | Excluded by the title |
| 260 | Deribew, 2016 | Trends, causes, and risk factors of mortality among children under 5 in Ethiopia, 1990-2013: findings from the Global Burden of Disease Study 2013 |  | Excluded by the title |
| 261 | Di Risio, 2016 | Living Optimally with HIV: Youth Experience in a Metropolitan Canadian City |  | Excluded by the title |
| 262 | Doyal, 2016 | Living with HIV and dying with AIDS: Diversity, inequality and human rights in the global pandemic |  | Excluded by the title |
| 263 | Dube, 2016 | Exploring pre-and post-partum barriers to anti-retroviral therapy adherence for HIV-positive women initiated onto Option B Plus in Harare, Zimbabwe |  | Excluded by the title |
| 264 | Dwoki, 2016 | Factors Affecting Adherence to Anti-retroviral Therapy at Kampala International University Teaching Hospital, Bushenyi District, Uganda |  | Excluded by the abstract |
| 265 | Dworkin, 2016 | Adherence to highly active antiretroviral therapy in Hyderabad, India: barriers, facilitators and identification of target groups |  | Excluded by the title |
| 266 | Elul, 2016 | Untangling the relationship between antiretroviral therapy use and incident pregnancy: A marginal structural model analysis using data from 47,313 HIV-positive women in East Africa | 10.1097/QAI.0000000000000963 | Excluded by the title |
| 267 | Ervin, 2016 | Surveillance and Azithromycin Treatment for Newcomers and Travelers Evaluation (ASANTE) Trial: Design and Baseline Characteristics | 10.1080/09286586.2016.1238947 | Excluded by the title |
| 268 | ESTHER, 2016 | SOCIAL SUPPORT AND ADHERENCE TO ANTI-RETROVIRAL THERAPY AMONG HIV PATIENTS IN UNIVERSITY OF PORT-HARCOURT TEACHING HOSPITAL, PORT-HARCOURT, NIGERIA |  | Excluded by the title |
| 269 | Gaida, 2016 | Incidence of neuropsychiatric side effects of efavirenz in HIV-positive treatment-naïve patients in public-sector clinics in the Eastern Cape |  | Excluded by the title |
| 270 | Garcia-Prats, 2016 | Outcome of culture-confirmed isoniazid-resistant rifampicin-susceptible tuberculosis in children | 10.5588/ijtld.16.0293 | Excluded by the title |
| 271 | Gebreegziabiher, 2016 | ASSESSMENT OF LEVEL OF ADHERENCE TO ANTI RETROVIRAL THERAPY AMONG ADULT HIV INFECTED PATIENTS IN MEKELLE HOSPITAL, NORTHERN ETHIOPIA |  | Excluded by the title |
| 272 | Govender, 2016 | Delayed diagnosis of anorectal malformations (ARM): causes and consequences in a resource-constrained environment |  | Excluded by the title |
| 273 | Haile, 2016 | Exploring spatial variations and factors associated with childhood stunting in Ethiopia: spatial and multilevel analysis |  | Excluded by the title |
| 274 | Hampanda, 2016 | Intimate partner violence and HIV-positive women's non-adherence to antiretroviral medication for the purpose of prevention of mother-to-child transmission in Lusaka, Zambia |  | Excluded by the title |
| 275 | Hargreaves, 2016 | HIV-related stigma and universal testing and treatment for HIV prevention and care: design of an implementation science evaluation nested in the HPTN 071 (PopART) cluster-randomized trial in Zambia and South Africa |  | Excluded by the title |
| 276 | Heestermans, 2016 | Determinants of adherence to antiretroviral therapy among HIV-positive adults in sub-Saharan Africa: a systematic review |  | Excluded by the title |
| 277 | Heise, 2016 | Greentree II: Violence against women and girls, and HIV |  | Excluded by the title |
| 278 | Ibinda, 2016 | Sodium Disturbances in Children Admitted to a Kenyan Hospital: Magnitude, Outcome and Associated Factors |  | Excluded by the title |
| 279 | Innes, 2016 | High Prevalence of Dyslipidemia and Insulin Resistance in HIV-infected Prepubertal African Children on Antiretroviral Therapy |  | Excluded by the title |
| 280 | Javalkar, 2016 | An estimation of mortality risks among people living with hiv in karnataka state, india: Learnings from an intensive hiv/aids care and support programme |  | Excluded by the title |
| 281 | John, 2016 | Religious Beliefs and Depression: Psychosocial Factors Affecting HIV Treatment Outcomes in South Africa |  | Excluded by the title |
| 282 | Khalil, 2016 | Burden of Diarrhea in the Eastern Mediterranean Region, 1990-2013: Findings from the Global Burden of Disease Study 2013 |  | Excluded by the title |
| 283 | Khan, 2016 | Risk factors for Mycobacterium tuberculosis infection in 2-4 year olds in a rural HIV-prevalent setting |  | Excluded by the title |
| 284 | Kiarie, 2016 | Influence Of Self-Reported Highly Active Anti Retroviral Therapy Side Effects On Adherence Among Persons With HIV Attending Tigoni District Hospital, Kenya |  | Excluded by the title |
| 285 | Kinyoki, 2016 | Environmental predictors of stunting among children under-five in Somalia: cross-sectional studies from 2007 to 2010 | 10.1186/s12889-016-3320-6 | Excluded by the title |
| 286 | Kinyoki, 2016 | Space-time mapping of wasting among children under the age of five years in Somalia from 2007 to 2010 |  | Excluded by the title |
| 287 | Kityo, 2016 | HIV Drug Resistance Among Children Initiating First-Line Antiretroviral Treatment in Uganda |  | Excluded by the title |
| 288 | Lazzerini, 2016 | Mortality and its risk factors in Malawian children admitted to hospital with clinical pneumonia, 2001-12: a retrospective observational study | 10.1016/s2214-109x(15)00215-6 | Excluded by the title |
| 289 | Lippman, 2016 | Evaluation of short message service and peer navigation to improve engagement in HIV care in South Africa: study protocol for a three-arm cluster randomized controlled trial |  | Excluded by the title |
| 290 | Lopez-Patton, 2016 | Childhood trauma and METH abuse among men who have sex with men: Implications for intervention |  | Excluded by the title |
| 290 | Lu, 2016 | Risk of poor development in young children in low-income and middle-income countries: an estimation and analysis at the global, regional, and country level |  | Excluded by the title |
| 292 | Madrid, 2016 | Hypoglycemia and Risk Factors for Death in 13 Years of Pediatric Admissions in Mozambique | 10.4269/ajtmh.15-0475 | Excluded by the title |
| 293 | Matey, 2016 | Lower prevalence of Entamoeba species in children with vertically transmitted HIV infection in Western Kenya | 10.1097/qad.0000000000001002 | Excluded by the title |
| 294 | Mathanga, 2016 | Patterns and determinants of malaria risk in urban and peri-urban areas of Blantyre, Malawi | 10.1186/s12936-016-1623-9 | Excluded by the title |
| 295 | Maulsby, 2016 | The global engagement in care convening: Recommended actions to improve health outcomes for people living with HIV |  | Excluded by the title |
| 296 | May, 2016 | Maternal nutritional status as a contributing factor for the risk of fetal alcohol spectrum disorders | 10.1016/j.reprotox.2015.11.006 | Excluded by the title |
| 297 | Mehta, 2016 | Adherence to antiretroviral therapy among children living with HIV in South India |  | Excluded by the title |
| 298 | Mohammadi Firouzeh, 2016 | Demographic, clinical and laboratory profiles of HIV infected patients admitted into Imam Khomeini Hospital of Tehran, Iran |  | Excluded by the title |
| 299 | Moramarco, 2016 | Community-Based Management of Child Malnutrition in Zambia: HIV/AIDS Infection and Other Risk Factors on Child Survival | 10.3390/ijerph13070666 | Excluded by the title |
| 300 | Moyo, 2016 | Urinary schistosomiasis among preschool children in Malengachanzi, Nkhotakota District, Malawi: Prevalence and risk factors | 10.4314/mmj.v28i1.3 | Excluded by the title |
| 301 | Mulelu, 2016 | Knowledge, Attitudes and Experiences of PeopleLiving with HIV who are on Antiretroviral Treatment at a Public Health Clinic in Limpopo, South Africa |  | Excluded by the title |
| 302 | Muluneh, 2016 | Rural children active trachoma risk factors and their interactions | 10.11604/pamj.2016.24.128.8790 | Excluded by the title |
| 303 | Muthuri, 2016 | Relationships between Parental Education and Overweight with Childhood Overweight and Physical Activity in 9-11 Year Old Children: Results from a 12-Country Study |  | Excluded by the title |
| 304 | Mutumba, 2016 | Examining the relationship between psychological distress and adherence to anti-retroviral therapy among Ugandan adolescents living with HIV |  | Excluded by the title |
| 305 | Namuyonga, 2016 | Cardiac dysfunction among Ugandan HIV-infected children on antiretroviral therapy | 10.1097/INF.0000000000000997 | Excluded by the title |
| 306 | Njolomole, 2016 | Determinants of HIV related stigma and discrimination among healthcare professionals at a health facility in Malawi |  | Excluded by the title |
| 307 | Nsagha, 2016 | A randomized controlled trial on the usefulness of mobile text phone messages to improve the quality of care of HIV and AIDS patients in Cameroon | 10.2174/1874613601610010093 | Excluded by the title |
| 308 | Nwankwo-Igomu, 2016 | Examining the Relationship of Family Support with Pediatric Adherence to HIV Antiretroviral Treatment in PEPFAR Care and Treatment Programs in Nigeria |  | Excluded by the title |
| 309 | Odetola, 2016 | Effects of a nursing intervention using a mobile phone application on Uptake of antenatal care, tetanus toxoids and malaria prevention among pregnant women in Nigeria |  | Excluded by the title |
| 310 | Odetola, 2016 | Effects of a nursing intervention using a mobile phone application on Uptake of antenatal care, tetanus toxoids and malaria prevention among pregnant women in Nigeria |  | Excluded by the title |
| 311 | Odetola, 2016 | Effects of mHealth Nursing Intervention on Uptake of Antenatal Care and Pregnancy Drugs Among Pregnant Women Attendees of PHC in Oyo State |  | Excluded by the title |
| 312 | Olofin, 2016 | Active Tuberculosis in HIV-Exposed Tanzanian Children up to 2 years of Age: Early-Life Nutrition, Multivitamin Supplementation and Other Potential Risk Factors | 10.1093/tropej/fmv073 | Excluded by the title |
| 313 | Olp, 2016 | Determinants of HIV related stigma and discrimination among healthcare professionals at a health facility in Malawi |  | Excluded by the title |
| 314 | Nsagha, 2016 | A randomized controlled trial on the usefulness of mobile text phone messages to improve the quality of care of HIV and AIDS patients in Cameroon | 10.2174/1874613601610010093 | Excluded by the title |
| 315 | Nwankwo-Igomu, 2016 | Examining the Relationship of Family Support with Pediatric Adherence to HIV Antiretroviral Treatment in PEPFAR Care and Treatment Programs in Nigeria |  | Excluded by the title |
| 316 | Odetola, 2016 | Effects of a nursing intervention using a mobile phone application on Uptake of antenatal care, tetanus toxoids and malaria prevention among pregnant women in Nigeria |  | Excluded by the title |
| 317 | Odetola, 2016 | Effects of mHealth Nursing Intervention on Uptake of Antenatal Care and Pregnancy Drugs Among Pregnant Women Attendees of PHC in Oyo State |  | Excluded by the title |
| 318 | Olofin, 2016 | Active Tuberculosis in HIV-Exposed Tanzanian Children up to 2 years of Age: Early-Life Nutrition, Multivitamin Supplementation and Other Potential Risk Factors |  | Excluded by the title |
| 319 | Olp, 2016 | Longitudinal analysis of the humoral response to Kaposi's sarcoma-associated herpesvirus after primary infection in children |  | Excluded by the title |
| 320 | Osingada, 2016 | Prevalence, barriers and factors associated with parental disclosure of their HIV positive status to children: a cross-sectional study in an urban clinic in Kampala, Uganda |  | Excluded by the title |
| 321 | PrayGod, 2016 | Indoor Air Pollution and Delayed Measles Vaccination Increase the Risk of Severe Pneumonia in Children: Results from a Case-Control Study in Mwanza, Tanzania | 10.1371/journal.pone.0160804 | Excluded by the title |
| 322 | Puga, 2016 | Still Far From 90-90-90: Virologic Outcomes of Children on Antiretroviral Therapy in Nurse-led Clinics in Rural Lesotho | 10.1097/INF.0000000000000929 | Excluded by the title |
| 323 | Qamar, 2016 | Aeromonas-Associated Diarrhea in Children Under 5 Years: The GEMS Experience | 10.4269/ajtmh.16-0321 | Excluded by the title |
| 324 | Randremanana, 2016 | Etiologies, Risk Factors and Impact of Severe Diarrhea in the Under-Fives in Moramanga and Antananarivo, Madagascar | 10.1371/journal.pone.0158862 | Excluded by the title |
| 325 | Randremanana, 2016 | Assessment of Diarrheal Disease Prevalence and Associated Risk Factors in Children of 6-59 Months Old at Adama District Rural Kebeles, Eastern Ethiopia, January/2015 | 10.1371/journal.pone.0158862 | Excluded by the title |
| 326 | Regassa, 2016 | Assessment of Diarrheal Disease Prevalence and Associated Risk Factors in Children of 6-59 Months Old at Adama District Rural Kebeles, Eastern Ethiopia, January/2015 | 10.4314/ejhs.v26i6.12 | Excluded by the title |
| 327 | Ricci, 2016 | Adherence to antiretroviral therapy of Brazilian HIV-infected children and their caregivers |  | Excluded by the title |
| 328 | Roberts, 2016 | Risk factors of malaria in children under the age of five years old in Uganda | 10.1186/s12936-016-1290-x | Excluded by the title |
| 329 | Rodriguez-Barraquer, 2016 | Quantifying Heterogeneous Malaria Exposure and Clinical Protection in a Cohort of Ugandan Children |  | Excluded by the title |
| 330 | Rohner, 2016 | Kaposi Sarcoma Risk in HIV-Infected Children and Adolescents on Combination Antiretroviral Therapy From Sub-Saharan Africa, Europe, and Asia | 10.1093/cid/ciw519 | Excluded by the title |
| 331 | Saile, 2016 | Children of the postwar years: A two-generational multilevel risk assessment of child psychopathology in northern Uganda |  | Excluded by the title |
| 332 | Semba, 2016 | Child Stunting is Associated with Low Circulating Essential Amino Acids |  | Excluded by the title |
| 333 | Semba, 2016 | The association of serum choline with linear growth failure in young children from rural Malawi |  | Excluded by the title |
| 334 | Seyoum, 2016 | Identification of different malaria patterns due to Plasmodium falciparum and Plasmodium vivax in Ethiopian children: a prospective cohort study |  | Excluded by the title |
| 335 | Shilaih, 2016 | Dually active HIV/HBV antiretrovirals as protection against incident hepatitis B infections: potential for prophylaxis |  | Excluded by the title |
| 336 | Shukla, 2016 | Nonadherence to antiretroviral therapy among people living with HIV/AIDS attending two tertiary care hospitals in district of northern India |  | Excluded by the title |
| 336 | {Altare, 2016 #7952} | Factors Associated with Stunting among Pre-school Children in Southern Highlands of Tanzania | 10.1093/tropej/fmw024 | Excluded by the title |
| 337 | Sileo, 2016 | The role of alcohol use in antiretroviral adherence among individuals living with HIV in South Africa: event-level findings from a daily diary study |  | Excluded by the title |
| 338 | Siregar, 2016 | HIV patients drop out in Indonesia: associated factors and potential productivity loss |  | Excluded by the title |
| 339 | Skeen, 2016 | Exposure to violence and psychological well-being over time in children affected by HIV/AIDS in South Africa and Malawi | 10.1080/09540121.2016.1146219 | Excluded by the title |
| 340 | Smith Fawzi, 2016 | Mental health and antiretroviral adherence among youth living with HIV in Rwanda |  | Excluded by the title |
| 341 | Strehlau, 2016 | HIV-associated neurodevelopmental delay: prevalence, predictors and persistence in relation to antiretroviral therapy initiation and viral suppression | 10.1111/cch.12399 | Excluded by the title |
| 342 | Sutcliffe, 2016 | A clinical guidance tool to improve the care of children hospitalized with severe pneumonia in Lusaka, Zambia |  | Excluded by the title |
| 343 | Tanna, 2016 | Analytical chemistry for assessing medication adherence |  | Excluded by the title |
| 344 | Tariku, 2016 | Vitamin-A deficiency and its determinants among preschool children: a community based cross-sectional study in Ethiopia |  | Excluded by the title |
| 345 | Taylor, 2016 | An exploration of the mechanism by which community health workers bring health gain to service users in England |  | Excluded by the title |
| 346 | Thandar, 2016 | Antiretroviral treatment adherence and associated factors among people living with HIV in developing country, Myanmar |  | Excluded by the title |
| 347 | Tilahun, 2016 | Treatment outcomes of childhood tuberculosis in Addis Ababa: a five-year retrospective analysis | 10.1186/s12889-016-3193-8 | Excluded by the title |
| 348 | Tomlinson, 2016 | Improving early childhood care and development, HIV-testing, treatment and support, and nutrition in Mokhotlong, Lesotho: study protocol for a cluster randomized controlled trial | 10.1186/s13063-016-1658-9 | Excluded by the title |
| 349 | Treffry-Goatley, 2016 | Understanding specific contexts of antiretroviral therapy adherence in rural South Africa: A thematic analysis of digital stories from a community with high HIV prevalence |  | Excluded by the title |
| 350 | Tusting, 2016 | Why is malaria associated with poverty? Findings from a cohort study in rural Uganda | 10.1186/s40249-016-0164-3 | Excluded by the title |
| 351 | Valerie, 2016 | GENETIC STRUCTURE AND GEOGRAPHICAL RELATIONSHIP OF SELECTED COLOCASIA ESCULENTA [L. SCHOTT] GERMPLASM USING SSRS |  | Excluded by the title |
| 352 | Waiswa, 2016 | Community and District Empowerment for Scale-up (CODES): a complex district-level management intervention to improve child survival in Uganda: study protocol for a randomized controlled trial | 10.1371/journal.pmed.1002408 | Excluded by the title |
| 353 | Abera, 2017 | Prevalence of malnutrition and associated factors in children aged 6-59 months among rural dwellers of damot gale district, south Ethiopia: community based cross sectional study | 10.1186/s12939-017-0608-9 | Excluded by the title |
| 354 | {Uvetie, 2020 #7051} | Factors Associated with Non-Adherence to Antiretroviral Therapy among HIV Infected Women Utilizing Reproductive and Child Health Services at Bugando Hospital Mwanza Tanzania |  | Excluded by the abstract |
| 355 | Abessa, 2017 | Developmental performance of hospitalized severely acutely malnourished under-six children in low- income setting | 10.1186/s12887-017-0950-5 | Excluded by the title |
| 356 | Akilimali, 2017 | Disclosure of HIV status and its impact on the loss in the follow-up of HIV-infected patients on potent anti-retroviral therapy programs in a (post-) conflict setting: A retrospective cohort study from Goma, Democratic Republic of Congo |  | Excluded by the title |
| 357 | Barffour, 2017 | High Iron Stores in the Low Malaria Season Increase Malaria Risk in the High Transmission Season in a Prospective Cohort of Rural Zambian Children | 10.3945/jn.117.250381 | Excluded by the title |
| 358 | Bassett, 2017 | Barriers to care and 1-year mortality among newly diagnosed HIV-infected people in Durban, South Africa |  | Excluded by the title |
| 359 | Bénet, 2017 | Severity of Pneumonia in Under 5-Year-Old Children from Developing Countries: A Multicenter, Prospective, Observational Study | 10.4269/ajtmh.16-0733 | Excluded by the title |
| 360 | Bernard, 2017 | Prevalence and factors associated with depression in people living with HIV in sub-Saharan Africa: a systematic review and meta-analysis |  | Excluded by the title |
| 361 | Birhanu, 2017 | Relationship between exposure to malaria and haemoglobin level of children 2-9 years old in low malaria transmission settings | 10.1016/j.actatropica.2017.05.021 | Excluded by the title |
| 362 | Birungi, 2017 | Assessing causal effects of early life-course factors on early childhood caries in 5-year-old Ugandan children using directed acyclic graphs (DAGs): A prospective cohort stu | 10.1111/cdoe.12314 | Excluded by the title |
| 363 | Bizuneh, 2017 | actors associated with diarrheal morbidity among under-five children in Jigjiga town, Somali Regional State, eastern Ethiopia: a cross-sectional study | 10.1186/s12887-017-0934-5 | Excluded by the title |
| 364 | Byakika-Kibwika, 2017 | Intravenous artesunate plus Artemisnin based Combination Therapy (ACT) or intravenous quinine plus ACT for treatment of severe malaria in Ugandan children: a randomized controlled clinical trial | 10.1186/s12879-017-2924-5 | Excluded by the title |
| 365 | Caggiano, 2017 | Factors That Negatively Affect the Prognosis of Pediatric Community-Acquired Pneumonia in District Hospital in Tanzania | 10.3390/ijms18030623 | Excluded by the title |
| 366 | Carlucci, 2017 | Prevalence and determinants of malaria among children in Zambézia Province, Mozambique |  | Excluded by the title |
| 367 | Chiao, 2017 | Community vulnerability and symptoms of acute respiratory infection among preschool age children in the Democratic Republic of Congo, Malawi and Nigeria: evidence from Demographic and Health Surveys |  | Excluded by the title |
| 368 | Chikwari, 2017 | Association between self-reported adherence and HIV viral load suppression among older children and adolescents |  | Excluded by the abstract |
| 369 | Chiwungwe, 2017 | Diabetes-related knowledge, attitudes and practices [KAP] of adult patients with type 2 diabetes in Maseru, Lesotho |  | Excluded by the title |
| 370 | Cho, 2017 | Technology-mediated interventions and quality of life for persons living with HIV/AIDS |  | Excluded by the title |
| 371 | Conan, 2017 | Animal-related factors associated with moderate-to-severe diarrhea in children younger than five years in western Kenya: A matched case-control study |  | Excluded by the title |
| 372 | Cox, 2017 | The prevalence and risk factors for acute respiratory infections in children aged 0-59 months in rural Malawi: A cross-sectional study |  | Excluded by the title |
| 373 | Croome, 2017 | Patient-reported barriers and facilitators to antiretroviral adherence in sub-Saharan Africa |  | Excluded by the title |
| 374 | Cruz, 2017 | actors Associated with Stunting among Children Aged 0 to 59 Months from the Central Region of Mozambique | 10.3390/nu9050491 | Excluded by the title |
| 375 | Davids, 2017 | Exploration of adherence to antiretroviral treatment amongst adolescents in a low socio-economic urban setting in Cape Town, South Africa |  | Excluded by the title |
| 376 | Derso, 2017 | Stunting, wasting and associated factors among children aged 6-24 months in Dabat health and demographic surveillance system site: A community based cross-sectional study in Ethiopia |  | Excluded by the title |
| 377 | Desyibelew, 2017 | Recovery rate and associated factors of children age 6 to 59 months admitted with severe acute malnutrition at inpatient unit of Bahir Dar Felege Hiwot Referral hospital therapeutic feeding unite, northwest Ethiopia | 10.1371/journal.pone.0171020 | Excluded by the title |
| 378 | Evangeli, 2017 | Patient-reported barriers and facilitators to antiretroviral adherence in sub-Saharan Africa |  | Excluded by the abstract |
| 379 | Cruz, 2017 | Factors Associated with Stunting among Children Aged 0 to 59 Months from the Central Region of Mozambique | 10.3390/nu9050491 | Excluded by the title |
| 380 | Davids, 2017 | Exploration of adherence to antiretroviral treatment amongst adolescents in a low socio-economic urban setting in Cape Town, South Africa |  | Excluded by the title |
| 381 | Evangeli, 2017 | The Adolescent HIV Disclosure Cognition and Affect Scale: Preliminary Reliability and Validity | 10.1093/jpepsy/jsw107 | Excluded by the title |
| 382 | Ferede, 2017 | Prevalence and determinants of active trachoma among preschool-aged children in Dembia District, Northwest Ethiopia |  | Excluded by the title |
| 383 | Fidhow, 2017 | Molecular epidemiology and associated risk factors of rotavirus infection among children < 5 yrs hospitalized for acute gastroenteritis in North Eastern, Kenya, 2012 |  | Excluded by the title |
| 384 | Fonsah, 2017 | Adherence to antiretroviral therapy (ART) in Yaoundé-Cameroon: association with opportunistic infections, depression, ART regimen and side effects |  | Excluded by the title |
| 385 | Franz, 2017 | Autism spectrum disorder in sub-saharan africa: A comprehensive scoping review |  | Excluded by the title |
| 386 | Gall, 2017 | Associations between selective attention and soil-transmitted helminth infections, socioeconomic status, and physical fitness in disadvantaged children in Port Elizabeth, South Africa: An observational study |  | Excluded by the title |
| 387 | Garrison, 2017 | Technological methods to measure adherence to antiretroviral therapy and preexposure prophylaxis |  | Excluded by the title |
| 388 | Gathara, 2017 | Variation in and risk factors for paediatric inpatient all-cause mortality in a low income setting: data from an emerging clinical information network |  | Excluded by the title |
| 389 | Gebrehiwot, 2017 | Utilization of dual contraception method among reproductive age women on antiretroviral therapy in selected public hospitals of Northern Ethiopia |  | Excluded by the title |
| 390 | Geletaw, 2017 | Hematologic abnormalities and associated factors among HIV infected children pre- and postantiretroviral treatment, North West Ethiopia |  | Excluded by the title |
| 391 | Gitonga, 2017 | Potential of mhealth interventions in management of obstetric fistula |  | Excluded by the title |
| 392 | Gone, 2017 | The association between malaria and malnutrition among under-five children in Shashogo District, Southern Ethiopia: a case-control study | 10.1186/s40249-016-0221-y | Excluded by the title |
| 393 | Gordon, 2017 | Research Priorities for the Intersection of Alcohol and HIV/AIDS in Low and Middle Income Countries: A Priority Setting Exercise |  | Excluded by the title |
| 394 | Green, 2017 | Safety Profile of Cough and Cold Medication Use in Pediatrics |  | Excluded by the title |
| 395 | Grudziak, 2017 | he effect of pre-existing malnutrition on pediatric burn mortality in a sub-Saharan African burn unit |  | Excluded by the title |
| 396 | Habyarimana, 2017 | Structured Additive Quantile Regression for Assessing the Determinants of Childhood Anemia in Rwanda |  | Excluded by the title |
| 397 | Hagos, 2017 | patial heterogeneity and risk factors for stunting among children under age five in Ethiopia: A Bayesian geo-statistical model |  | Excluded by the title |
| 398 | Hasegawa, 2017 | Development of a screening tool to predict malnutrition among children under two years old in Zambia |  | Excluded by the title |
| 399 | Hategekimana, 2017 | The Role of Faith Bases Organizations in Enhancement of the Social Welfare Among the Rural Poor Households in Nyamweru sub-county, Kabale district; A case Study of Caritas Kabale diocese |  | Excluded by the title |
| 400 | Hershey, 2017 | Malaria Control Interventions Contributed to Declines in Malaria Parasitemia, Severe Anemia, and All-Cause Mortality in Children Less Than 5 Years of Age in Malawi, 2000-2010 | 10.4269/ajtmh.17-0203 | Excluded by the title |
| 401 | Higdon, 2017 | Should Controls With Respiratory Symptoms Be Excluded From Case-Control Studies of Pneumonia Etiology? Reflections From the PERCH Study |  | Excluded by the title |
| 402 | Himaubi, 2017 | Motivating factors to antiretroviral treatment adherence by people living with HIV in Ndola district |  | Excluded by the title |
| 403 | Jaacks, 2017 | Programming maternal and child overweight and obesity in the context of undernutrition: current evidence and key considerations for low- and middle-income countries | 10.1017/s1368980016003323 | Excluded by the title |
| 404 | Jelle, 2017 | The REFANI-S study protocol: a non-randomised cluster controlled trial to assess the role of an unconditional cash transfer, a non-food item kit, and free piped water in reducing the risk of acute malnutrition among children aged 6-59 months living in camps for internally displaced persons in the Afgooye corridor, Somalia | 10.1186/s12889-017-4550-y | Excluded by the title |
| 405 | Jérome, 2017 | Sociodemographic and Nutritional Factors Associated with Adherence to Antiretroviral Therapy in PLWHA in Benin |  | Excluded by the title |
| 406 | Kabaghe, 2017 | Short-Term Changes in Anemia and Malaria Parasite Prevalence in Children under 5 Years during One Year of Repeated Cross-Sectional Surveys in Rural Malawi | 10.4269/ajtmh.17-0335 | Excluded by the title |
| 407 | Kakooza-Mwesige, 2017 | Prevalence of cerebral palsy in Uganda: a population-based study | 10.1016/s2214-109x(17)30374-1 | Excluded by the title |
| 408 | Kapata, 2017 | Integration of HIV care and family planning: attitudes, challenges and opportunities in Lusaka, Zambia |  | Excluded by the title |
| 409 | Kariuki, 2017 | Burden, risk factors, and comorbidities of behavioural and emotional problems in Kenyan children: a population-based study | 10.1016/s2215-0366(16)30403-5 | Excluded by the title |
| 410 | Kariuki, 2017 | Influence of Health Literacy on Antiretroviral Treatment Adherence Among HIV/AIDS Infected Adolescents in Thika Level 5 Hospital, Kiambu County |  | Excluded by the title |
| 411 | Kashala Abotnes, 2017 | Disclosure of HIV status and its impact on the loss in the follow-up of HIV-infected patients on potent anti-retroviral therapy programs ina (post-) conflict setting: A retrospective cohort study from Goma, Democratic Republic of Congo |  | Excluded by the title |
| 412 | Keitel, 2017 | A novel electronic algorithm using host biomarker point-of-care tests for the management of febrile illnesses in Tanzanian children (e-POCT): A randomized, controlled non-inferiority trial | 10.1371/journal.pmed.1002411 | Excluded by the title |
| 413 | Kemp, 2017 | Pre/post evaluation of a pilot prevention with positives training program for healthcare providers in North West Province, Republic of South Africa |  | Excluded by the title |
| 414 | Khumalo, 2017 | Knowledge, attitudes and perceptions of males with regard to medical male circumcision |  | Excluded by the title |
| 415 | Kim, 2017 | High self-reported non-adherence to antiretroviral therapy amongst adolescents living with HIV in Malawi: Barriers and associated factors | 10.7448/IAS.20.1.21437 | Excluded by the title |
| 416 | Kronfli, 2017 | Understanding the correlates of attrition associated with antiretroviral use and viral suppression among women living with HIV in Canada |  | Excluded by the title |
| 417 | Kuziga, 2017 | Prevalence and factors associated with anaemia among children aged 6 to 59 months in Namutumba district, Uganda: a cross- sectional study |  | Excluded by the title |
| 418 | Laibon, 2017 | Factors that Influence Treatment Adherence among Male Sex Workers on Antiretroviral Therapy in Nairobi City County, Kenya |  | Excluded by the title |
| 419 | Langebeek, 2017 | Treatment adherence, health related quality of life and aging in HIV-1 infected patients |  | Excluded by the title |
| 420 | Lawn, 2017 | Group B Streptococcal Disease Worldwide for Pregnant Women, Stillbirths, and Children: Why, What, and How to Undertake Estimates? | 10.1093/cid/cix653 | Excluded by the title |
| 421 | le Roux, 2017 | Community-acquired pneumonia in children - a changing spectrum of disease |  | Excluded by the title |
| 422 | Lilian, 2017 | A 10-year cohort analysis of routine paediatric ART data in a rural South African setting |  | Excluded by the title |
| 423 | Lodebo, 2017 | Knowledge about mother-to-child transmission of HIV, its prevention and associated factors among Ethiopian women |  | Excluded by the title |
| 420 | Luma, 2017 | Cross-sectional assessment of three commonly used measures of adherence to combination antiviral therapy in a resource limited setting |  | Excluded by the title |
| 421 | Mafigiri, 2017 | Risk factors for measles death: Kyegegwa District, western Uganda, February-September, 2015 |  | Excluded by the title |
| 422 | Manyanga, 2017 | Socioeconomic status and dietary patterns in children from around the world: different associations by levels of country human development? |  | Excluded by the title |
| 423 | {Mariga, 2017 #7534} | Peer Support and retention in HIV care for sub-Saharan African and Caribbean migrant women: The Positive Sisters program in the Netherlands |  | Excluded by the title |
| 424 | Martínez, 2017 | Kathryn P. Derose, Melissa Felician, Bing Han, Kartika Palar, Blanca Ramírez, Hugo Farías, and |  | Excluded by the title |
| 425 | Mbengue, 2017 | Predictors of adherence among antiretroviral therapy naïve patients on first-line regimen at Themba Lethu Clinic inJohannesburg: Results from a prospective cohort study |  | Excluded by the title |
| 426 | Mbethe, 2017 | Incidence of refeeding syndrome and its associated factors in South African children hospitalized with severe acute malnutrition |  | Excluded by the title |
| 427 | McCulloch, 2017 | Globalization of pediatric transplantation: The risk of tuberculosis or not tuberculosis |  | Excluded by the title |
| 428 | McMahon, 2017 | Stigma, Facility Constraints, and Personal Disbelief: Why Women Disengage from HIV Care During and After Pregnancy in Morogoro Region, Tanzania | 10.1007/s10461-016-1505-8 | Excluded by the title |
| 429 | Mehari, 2017 | Factors affecting treatment adherence among HIV-positive patients in Eritrea |  | Excluded by the abstract |
| 430 | Mgongo, 2017 | Underweight, Stunting and Wasting among Children in Kilimanjaro Region, Tanzania; a Population-Based Cross-Sectional Study | 10.3390/ijerph14050509 | Excluded by the title |
| 431 | Mgosha, 2017 | Barriers to switching patients to second-line antiretroviral treatment among clinicians in Tanzania |  | Excluded by the title |
| 432 | Mohr, 2017 | DOT or SAT for Rifampicin-resistant tuberculosis? A non-randomized comparison in a high HIV-prevalence setting |  | Excluded by the title |
| 433 | Moraleda, 2017 | Anaemia in hospitalised preschool children from a rural area in Mozambique: a case control study in search for aetiological agents | 10.1186/s12887-017-0816-x | Excluded by the title |
| 434 | {Seetharaman, 2015 #7171} | Adherence to Anti Retroviral Therapy of People Living with HIV/AIDS: a Cross Sectional Survey |  | Excluded by the abstract |
| 435 | Mthethwa, 2017 | Measure of adherence to antiretroviral treatment amongst HIV positive patients attending antiretroviral clinics in selected rural, deep-rural and semi-urban areas of Ugu District in KwaZulu-Natal |  | Excluded by the title |
| 436 | Müller, 2017 | Shrinking risk profiles after deworming of children in Port Elizabeth, South Africa, with special reference to Ascaris lumbricoides and Trichuris trichiura | 10.4081/gh.2017.601 | Excluded by the title |
| 437 | Mumm, 2017 | Exploring urban health in Cape Town, South Africa: an interdisciplinary analysis of secondary data | 10.1080/20477724.2016.1275463 | Excluded by the title |
| 438 | Munthali, 2017 | Early Life Growth Predictors of Childhood Adiposity Trajectories and Future Risk for Obesity: Birth to Twenty Cohort | 10.1089/chi.2016.0310 | Excluded by the title |
| 439 | Muri, 2017 | Development of HIV drug resistance and therapeutic failure in children and adolescents in rural Tanzania: An emerging public health concern | 10.1097/QAD.0000000000001273 | Excluded by the title |
| 440 | Mutagoma, 2017 | Syphilis and HIV prevalence and associated factors to their co-infection, hepatitis B and hepatitis C viruses prevalence among female sex workers in Rwanda | 10.1186/s12879-017-2625-0 | Excluded by the title |
| 441 | Sekine, 2023 | Aderence to anti-retroviral therapy, decisional conflicts, and health-related quality of life among treatment-naïve individuals living with HIV: a DEARS-J observational study |  | Excluded by the abstract |
| 442 | Mutasa‐Apollo, 2017 | Effect of frequency of clinic visits and medication pick‐up on antiretroviral treatment outcomes: a systematic literature review and meta‐analysis |  | Excluded by the title |
| 443 | Mutsigiri-Murewanhema, 2017 | Factors associated with severe malaria among children below ten years in Mutasa and Nyanga districts, Zimbabwe, 2014-2015 | 10.11604/pamj.2017.27.23.10957 | Excluded by the title |
| 444 | Mwalumuli, 2017 | Comparison of level and predictors of adherence to art option b+ between HIV infected pregnant and lactating women at Mnazi Mmoja hospital Dar es salam Tanzania |  | Excluded by the title |
| 445 | Mwanguhya, 2017 | Factors that affect adherence to anti-retroviral therapy in HIV positive clients at Comboni Hospital Kyamuhunga Bushenyi Uganda |  | Excluded by the title |
| 446 | Nakimuli-Mpungu, 2017 | Process evaluation of a randomized controlled trial of group support psychotherapy for depression treatment among people with HIV/AIDS in Northern Uganda |  | Excluded by the title |
| 447 | Namara, 2017 | Effects of treating helminths during pregnancy and early childhood on risk of allergy-related outcomes: Follow-up of a randomized controlled trial | 10.1111/pai.12804 | Excluded by the title |
| 448 | Nath, 2017 | Pediatric HIV in India: Current scenario and the way forward |  | Excluded by the title |
| 449 | Ngari, 2017 | Mortality after Inpatient Treatment for Severe Pneumonia in Children: a Cohort Study | 10.1111/ppe.12348 | Excluded by the title |
|  | Noiman, 2017 | CHANGES in adherence and program retention and associated factors among hiv-infected women receiving option b+ for preventing mother-to-child transmission of hiv in Kampala, Uganda: a mixed methods approach |  | Excluded by the title |
| 450 | Nyamhanga, 2017 | Prevention of mother to child transmission of HIV in Tanzania: assessing gender mainstreaming on paper and in practice |  | Excluded by the title |
| 451 | Nyandwi, 2017 | Schistosomiasis mansoni incidence data in Rwanda can improve prevalence assessments, by providing high-resolution hotspot and risk factors identification | 10.1186/s12889-017-4816-4 | Excluded by the title |
| 450 | Nyati-Jokomo, 2017 | Risk factors for schistosomiasis transmission among school children in Gwanda district, Zimbabwe | 10.1016/j.actatropica.2017.03.033 | Excluded by the title |
| 451 | Chirundu, 2018 | Adherence to antiretroviral therapy among clients utilizing a primary health care facility Kadoma Zimbabwe (2016) |  | Excluded by the abstract |
| 452 | Osafo, 2017 | The experiences of caregivers of children living with HIV and AIDS in Uganda: a qualitative study |  | Excluded by the title |
| 453 | Ovnat Tamir, 2017 | Acute otitis media guidelines in selected developed and developing countries: uniformity and diversity | 10.1136/archdischild-2016-310729 | Excluded by the title |
| 454 | Pramila, 2017 | Effect of adherence to anti-retroviral therapy on cd4 t cells and hiv viral load in Nepalese Tertiary Care Hospital |  | Excluded by the title |
| 455 | Pranithram, 2017 | Factors influencing adherence to Anti-retroviral therapy in early and late treatment HIV groups in a teaching hospital, India; a qualitative cross-sectional study |  | Excluded by the title |
| 456 | {Chirundu, 2018 #7533} | Adherence to antiretroviral therapy among clients utilizing a primary health care facility Kadoma Zimbabwe (2016) | {Chirundu, 2018 #7533} | Excluded by the abstract |
| 457 | Rasooli-Nejad, 2017 | Assessing the efficacy of second-line antiretroviral treatment for hiv patients failing first-line antiretroviral therapy in iran: a cohort study |  | Excluded by the title |
| 458 | Rathbone, 2017 | A systematic review and thematic synthesis of patients' experience of medicines adherence |  | Excluded by the title |
| 459 | Razanamihaja, 2017 | The assessment of status of tobacco smoking among urban primary schoolchildren in Madagascar | 10.1093/heapro/daw077 | Excluded by the title |
| 460 | Robert, 2017 | Gender-based violence and adherence to anti-retroviral therapy among HIV-infected women attending care and treatment clinic, Mbeya Tanzania |  | Excluded by the title |
| 461 | Rode, 2017 | Experience and outcomes of micrografting for major paediatric burns | 10.1016/j.burns.2017.02.008 | Excluded by the title |
| 462 | Rogawski, 2017 | Determinants and Impact of Giardia Infection in the First 2 Years of Life in the MAL-ED Birth Cohort | 10.1093/jpids/piw082 | Excluded by the title |
| 463 | Oluwaseun, 2021 | Determinants contributing to adherence with antiretroviral regimen of people living with HIV/AIDS in Babcock University Teaching Hospital |  | Excluded by the title |
| 464 | Izizag, 2020 | Déterminants de la non-observance au traitement antirétroviral chez l’adulte à Kinshasa |  | Excluded by the title |
| 465 | Adeniran, 2021 | Determinants of Adherence among Patients on Highly Active Anti-Retroviral Therapy in Lagos State, Nigeria |  | Excluded by the title |
| 466 | Ondiek, 2018 | Determinants of adherence to anti-retroviral therapy among discordant couples in Usigu Division, Bondo sub-county, Kenya |  | Excluded by the title |
| 467 | {Heestermans, 2016 | Determinants of adherence to antiretroviral therapy among HIV-positive adults in sub-Saharan Africa: a systematic review |  | Excluded by the title |
| 468 | Beletew, 2020 # | Determinants of Anemia among HIV-Positive Children on Highly Active Antiretroviral Therapy Attending Hospitals of North Wollo Zone, Amhara Region, Ethiopia, 2019: A Case-Control Study | 10.1155/2020/3720572 | Excluded by the title |
| 469 | Alamneh, 2023 | among children aged 6-59 months in Ethiopia: Multilevel Bayesian statistical approach | 10.1038/s41598-022-20381-7 | Excluded by the title |
| 470 | Ahmadi, 2018 | Determinants of child anthropometric indicators in Ethiopia | 10.1186/s12889-018-5541-3 | Excluded by the title |
| 471 | Ramlagan, 2018 | Determinants of disclosure and non-disclosure of HIV-positive status, by pregnant women in rural South Africa |  | Excluded by the title |
| 472 | Njolomole, 2016 | Determinants of HIV related stigma and discrimination among healthcare professionals at a health facility in Malawi |  | Excluded by the title |
| 473 | Anjajo, 2023 | Determinants of hypertension among diabetic patients in southern Ethiopia: a case-control study | 10.1186/s12872-023-03245-4 | Excluded by the title |
| 474 | Bifftu, 2022 | Determinants of Intimate Partner Violence against Pregnant Women in Ethiopia: A Systematic Review and Meta-Analysis | 10.1155/2022/4641343 | Excluded by the title |
| 475 | Wanjiku, 2022 | Determinants of Involvement in Antenatal Care among Male Police Officers at Anti-Stock Theft Unit in Gilgil Ward, Nakuru County; Kenya |  | Excluded by the title |
| 476 | KEBAYA, 2023 | Determinants of linkage to HIV care and treatment among men who have sex with men (msm) in Kisumu county, Kenya |  | Excluded by the title |
| 477 | Semakula, 2023 | Determinants of malaria infections among children in refugee settlements in Uganda during 2018-2019 | 10.1186/s40249-023-01090-3 | Excluded by the title |
| 478 | MULI, 2023 | Determinants of mother to child transmission of HIV among exposed infants in Kericho County Referral Hospital, Kenya |  | Excluded by the title |
| 479 | Izizag, 2020 | Determinants of non-compliance with antiretroviral therapy in adult patients in Kinshasa |  | Excluded by the title |
| 480 | Amare, 2019 | Determinants of nutritional status among children under age 5 in Ethiopia: further analysis of the 2016 Ethiopia demographic and health survey | 10.1186/s12992-019-0505-7 | Excluded by the title |
| 481 | Dhlakama, 2023 | Determinants of option B+ treatment adherence among HIV-positive breastfeeding women in Zimbabwe | 10.29063/ajrh2023/v27i12.2 | Excluded by the title |
| 482 | Seramo, 2022 | Determinants of pneumonia among children attending public health facilities in Worabe town | 10.1038/s41598-022-10194-z | Excluded by the title |
| 483 | Fikadu, 2021 | Determinants of pre-eclampsia among pregnant women attending perinatal care in hospitals of the Omo district, Southern Ethiopia | 10.1111/jch.14073 | Excluded by the title |
| 484 | Asfaw, 2020 | Determinants of soil-transmitted helminth infections among pre-school-aged children in Gamo Gofa zone, Southern Ethiopia: A case-control study | 10.1371/journal.pone.0243836 | Excluded by the title |
| 485 | Rakotomanana, 2017 | Determinants of stunting in children under 5 years in Madagascar | 10.1111/mcn.12409 | Excluded by the title |
| 486 | Woodruff, 2017 | Determinants of stunting reduction in Ethiopia 2000 - 2011 | 10.1111/mcn.12307 | Excluded by the title |
| 487 | Chadambuka, 2019 | Determinants of Treatment Adherence and Retention in Care among HIV Positive Pregnant and Breastfeeding Women in a Rural District in Zimbabwe |  | Excluded by the title |
| 488 | Ogbo, 2019 | Determinants of trends in neonatal, post-neonatal, infant, child and under-five mortalities in Tanzania from 2004 to 2016 | 10.1186/s12889-019-7547-x | Excluded by the title |
| 489 | Kassie, 2020 | Determinants of under-nutrition among children under five years of age in Ethiopia | 10.1186/s12889-020-08539-2 | Excluded by the title |
| 490 | Ally, 2023 | Determinants of viral load suppression among orphaned and vulnerable children living with HIV on ART in Tanzania | 10.3389/fpubh.2023.1076614 | Excluded by the title |
| 491 | Elashi, 2021 | Determinants of viral suppression among adolescents on antiretroviral therapy in Thabo Mofutsanyane District Municipality, Free State province, South Africa |  | Excluded by the title |
| 492 | Shumetie, 2021 | Determinants of Virological Failure AmongHIV-Infected Children on First-Line Antiretroviral Therapy in West Gojjam Zone, Amhara Region, Ethiopia | 10.2147/HIV.S334067 | Excluded by the title |
| 493 | Dawa, 2019 | Developing a seasonal influenza vaccine recommendation in Kenya: Process and challenges faced by the National Immunization Technical Advisory Group (NITAG) | 10.1016/j.vaccine.2018.11.062 | Excluded by the title |
| 494 | Karugaba, 2020 | Developing policy guidelines to promote quality of life of young adults with perinatally acquired HIV in Botswana |  | Excluded by the title |
| 495 | Swendeman, 2015 | Development and pilot testing of daily interactive voice response (IVR) calls to support antiretroviral adherence in India: a mixed-methods pilot study |  | Excluded by the title |
| 496 | Ashaba, 2019 | Development and validation of a 20-item screening scale to detect major depressive disorder among adolescents with HIV in rural Uganda: A mixed-methods study |  | Excluded by the title |
| 497 | Mutumba, 2015 | Development of a psychosocial distress measure for Ugandan adolescents living with HIV |  | Excluded by the title |
| 498 | Wright, 2021 | Development of a Scale to Measure Infant Eating Behaviour Worldwide | 10.3390/nu13082495 | Excluded by the title |
| 499 | Hasegawa, 2017 | Development of a screening tool to predict malnutrition among children under two years old in Zambia | 10.1080/16549716.2017.1339981 | Excluded by the title |
| 500 | Kaimila, 2019 | Development of Acute Malnutrition Despite Nutritional Supplementation in Malawi | 10.1097/mpg.0000000000002241 | Excluded by the title |
| 501 | Davies, 2022 | Effect of antiretroviral therapy care interruptions on mortality in children living with HIV | 10.1097/QAD.0000000000003194 | Excluded by the title |
| 502 | Mbita, 2019 | Effect of antiretroviral therapy on fertility rate among women living with HIV in Tabora, Tanzania: An historical cohort study | 10.1371/journal.pone.0222173 | Excluded by the title |
| 503 | Velloza, 2018 | Effect of depression on adherence to oral PrEP among men and women in East Africa |  | Excluded by the title |
| 504 | Mugo, 2023 | Effect of Dolutegravir and Multimonth Dispensing on Viral Suppression among Children with HIV | 10.1097/QAI.0000000000003190 | Excluded by the title |
| 505 | Ezenwosu, 2023 | Effect of dolutegravir-based drug combinations on the level of medication adherence and viral load among adolescents living with HIV in resource-limited setting: a pre-post design |  | Excluded by the title |
| 506 | Mutasa‐Apollo, 2017 | Effect of frequency of clinic visits and medication pick‐up on antiretroviral treatment outcomes: a systematic literature review and meta‐analysis |  | Excluded by the title |
| 507 | Nakimuli-Mpungu, 2023 | The effect of group support psychotherapy on adherence to anti-retroviral therapy and viral suppression among HIV positive young people: Study protocol for a pilot randomized controlled trial |  | Excluded by the title |
| 508 | Tjituka, 2018 | disclosure on the psychosocial wellbeing, adherence levels and treatment outcomes of adolescents on Highly Active Anti-Retroviral Therapy (HAART) at Katututra State Hospital, Namibia |  | Excluded by the title |
| 509 | YILMAZER, 2020 | Effect of Information-Motivation-Behavioral Skills Model-Based Intervention on Quality of Life of Ostomy Patients |  | Excluded by the title |
| 510 | Ambarwati, 2021 | he effect of iron deficiency anemia on the child death related to HIV/AIDS infection: meta-analysis |  | Excluded by the title |
| 511 | Starck, 2022 | The effect of malaria on childhood anemia in a quasi-experimental study of 7,384 twins from 23 Sub-Saharan African countries | 10.3389/fpubh.2022.1009865 | Excluded by the title |
| 512 | Starck, 2021 | The effect of malaria on haemoglobin concentrations: a nationally representative household fixed-effects study of 17,599 children under 5 years of age in Burkina Faso | 10.1186/s12936-021-03948-z | Excluded by the title |
| 513 | Blessy, 2016 | Effect of micro and macro nutritient supplementation on disease outcome in adolescents with HIV on HAART: A Randomised Double-blinded clinical trial |  | Excluded by the title |
| 514 | Grudziak, 2017 | The effect of pre-existing malnutrition on pediatric burn mortality in a sub-Saharan African burn unit | 10.1016/j.burns.2017.03.022 | Excluded by the title |
| 515 | Akindele, 2015 | The effect of socio-economic status on adherence to anti-retroviral therapy |  | Excluded by the title |
| 516 | Bitew, 2018 | The effect of SODIS water treatment intervention at the household level in reducing diarrheal incidence among children under 5 years of age: a cluster randomized controlled trial in Dabat district, northwest Ethiopia | 10.1186/s13063-018-2797-y | Excluded by the title |
| 517 | Kaswa, 2023 | The effect of substance uses on antiretroviral therapy adherence among people living with HIV in Mthatha, Eastern Cape |  | Excluded by the  abstract |
| 518 | Tadesse, 2017 | Effect of water, sanitation and hygiene interventions on active trachoma in North and South Wollo zones of Amhara Region, Ethiopia: A Quasi-experimental study | 10.1371/journal.pntd.0006080 | Excluded by the title |
| 519 | Magnolini, 2024 | Effectiveness and acceptance of group therapy as a mental health intervention for people living with HIV in Africa–a scoping literature review | 10.1186/s12889-023-16181-x | Excluded by the title |
| 520 | Chinoda, 2020 | Effectiveness of a peer-led adolescent mental health intervention on HIV virological suppression and mental health in Zimbabwe: protocol of a cluster-randomised trial |  | Excluded by the title |
| 521 | Soboka, 2015 | The effectiveness of counseling, material support and/or nutritional supplementation on improving adherence to anti-retroviral therapy and clinical outcomes among HIV patients: a systematic review of quantitative evidence protocol |  | Excluded by the title |
| 522 | Sakthivel, 2021 | Effectiveness of HIV Intervention Package (HIP) on HIV Infected Adolescents: Pilot Study Report |  | Excluded by the title |
| 523 | Prisma, 2023 | The Effectiveness of Mobile Phone Text Messages on the Adherence of Antiretroviral Drug Taking in Patients Living with HIV/AIDS: A Meta-Analysis |  | Excluded by the title |
| 524 | bin Abdul Wahab, 2021 | Effectiveness of Phone Reminders to Improve Adherence to Anti-Retroviral Therapy: A Meta-Analysis |  | Excluded by the title |
| 525 | Ribeiro, 2015 | Effectiveness of psycho-educational intervention in HIV patients’ treatment |  | Excluded by the title |
| 526 | Cairns, 2021 | Effectiveness of seasonal malaria chemoprevention (SMC) treatments when SMC is implemented at scale: Case-control studies in 5 countries | 10.1371/journal.pmed.1003727 | Excluded by the title |
| 527 | Odetola, 2016 | Effects of a nursing intervention using a mobile phone application on Uptake of antenatal care, tetanus toxoids and malaria prevention among pregnant women in Nigeria |  | Excluded by the title |
| 528 | Muralidharan, 2015 | Effects of ART on CD4 Count and Body Weight in HIV/AIDS Patients using Longitudinal Analysis in the Case of Debre Berhan Referral Hospital |  | Excluded by the title |
| 530 | Crea, 2015 | Effects of cash transfers on Children's health and social protection in Sub-Saharan Africa: differences in outcomes based on orphan status and household assets | 10.1186/s12889-015-1857-4 | Excluded by the title |
| 531 | Sabourin, 2023 | Effects of Maternal HIV Infection on Early Kaposi Sarcoma-Associated Herpesvirus Seroconversion in a Ken an Mother-Infant Cohort | 10.1093/infdis/jiad310 | Excluded by the title |
| 532 | Odetola, 2016 | Effects of mHealth Nursing Intervention on Uptake of Antenatal Care and Pregnancy Drugs Among Pregnant Women Attendees of PHC in Oyo State |  | Excluded by the title |
| 533 | Strother, 2022 | Effects of psychosocial factors on nonadherence to ART in Ganta, Nimba county, Liberia |  | Excluded by the title |
| 534 | Ugburo, 2015 | Effects of telephonic SMS reminders influence on adherence to scheduled medication pick up appointments among adults on antiretrovirals at the Swakopmund State Hospital ART clinic Namibia |  | Excluded by the title |
| 535 | Namara, 2017 | Effects of treating helminths during pregnancy and early childhood on risk of allergy-related outcomes: Follow-up of a randomized controlled trial | 10.1111/pai.12804 | Excluded by the title |
| 536 | Nguyen, 2022 | Effects of Two Alcohol Reduction Interventions on Depression and Anxiety Symptoms of ART Clients in Vietnam | 10.1007/s10461-021-03532-1 | Excluded by the title |
| 537 | Bawn, 2022 | eHealth for family planning in Botswana: acceptability and feasibility |  | Excluded by the title |
| 538 | Nkwana, 2019 | Ellisras Longitudinal Study 2017: the association of fat patterning with blood pressure in Polokwane private school children aged five to 15 years (ELS 22) | 10.5830/cvja-2018-058 | Excluded by the title |
| 539 | Moonsamy, 2019 | Emergent Literacy Support for Children from Marginalised Populations | 10.1159/000493893 | Excluded by the title |
| 540 | Vinayagamoorthy, 2023 | Emergomycosis, an Emerging Thermally Dimorphic Fungal Infection: A Systematic Review |  | Excluded by the title |
| 541 | Kefale, 2019 | Emotional and behavioral problems and associated factors among children and adolescents on highly active anti-retroviral therapy in public hospitals of West Gojjam zone, Amhara regional state of Ethiopia, 2018: A cross-sectional study | 10.1186/s12887-019-1453-3 | Excluded by the title |
| 542 | Ouma, 2020 | Indothelial Activation, Acute Kidney Injury, and Cognitive Impairment in Pediatric Severe Malaria | 10.1097/ccm.0000000000004469 | Excluded by the title |
| 543 | Nkenfou, 2021 | Enhanced passive surveillance dengue infection among febrile children: Prevalence, co-infections and associated factors in Cameroon | 10.1371/journal.pntd.0009316 | Excluded by the title |
| 544 | Tsai, 2019 | Enteric Pathogen Diversity in Infant Foods in Low-Income Neighborhoods of Kisumu, Kenya | 10.3390/ijerph16030506 | Excluded by the title |
| 545 | Feleke, 2018 | Enteric pathogens and associated risk factors among under-five children with and without diarrhea in Wegera District, Northwestern Ethiopia | 10.11604/pamj.2018.29.72.13973 | Excluded by the title |
| 546 | Khabo-Mmekoa, 2022 | Enteric Pathogens Risk Factors Associated with Household Drinking Water: A Case Study in Ugu District Kwa-Zulu Natal Province, South Africa | 10.3390/ijerph19084431 | Excluded by the title |
| 547 | Zelelie, 2019 | Enteropathogens in Under-Five Children with Diarrhea in Health Facilities of Debre Berhan Town, North Shoa, Ethiopia | 10.4314/ejhs.v29i2.7 | Excluded by the title |
| 548 | Satzen, 2023 | Environmental Factors Associated with Late Initiation of Antiretroviral Therapy among People Living With HIV/AIDS in Jos, Plateau State, Nigeria: Implications for Psychotherapy |  | Excluded by the title |
| 549 | Kinyoki, 2016 | Environmental predictors of stunting among children under-five in Somalia: cross-sectional studies from 2007 to 2010 | 10.1186/s12889-016-3320-6 | Excluded by the title |
| 550 | Yeruva, 2022 | Enzyme Responsive Delivery of Anti-Retroviral Peptide via Smart Hydrogel |  | Excluded by the title |
| 551 | Musa, 2021 | Factors associated with nonadherence to antiretroviral therapy among children with HIV/AIDS in Ahmadu Bello University Teaching Hospital, Zaria, Nigeria |  | Excluded by the title |
| 552 | Tsegaye, 2023 | Epidemiological survival pattern, risk factors, and estimated time to develop tuberculosis after test and treat strategies declared for children living with human immune deficiency virus | 10.1016/j.ijtb.2023.05.008 | Excluded by the title |
| 553 | Bauleth, 2020 | Epidemiology and factors associated with diarrhoea among children under five years of age in the Engela District in the Ohangwena Region, Namibia | 10.4102/phcfm.v12i1.2361 | Excluded by the title |
| 554 | Korpe, 2018 | Epidemiology and Risk Factors for Cryptosporidiosis in Children From 8 Low-income Sites: Results From the MAL-ED Study | 10.1093/cid/ciy355 | Excluded by the title |
| 555 | Bigna, 2018 | Epidemiology of depressive disorders in people living with HIV in Africa: a systematic review and meta-analysis |  | Excluded by the title |
| 556 | Bigna, 2019 | Epidemiology of depressive disorders in people living with HIV in Africa: a systematic review and meta-analysis: Burden of depression in HIV in Africa |  | Excluded by the title |
| 557 | Bwogi, 2016 | The epidemiology of rotavirus disease in under-five-year-old children hospitalized with acute diarrhea in central Uganda, 2012-2013 | 10.1007/s00705-015-2742-2 | Excluded by the title |
| 558 | Rouhani, 2022 | The Epidemiology of Sapovirus in the Etiology, Risk Factors, and Interactions of Enteric Infection and Malnutrition and the Consequences for Child Health and Development Study: Evidence of Protection Following Natural Infection | 10.1093/cid/ciac165 | Excluded by the title |
| 559 | Tefera, 2020 | Epidemiology of Schistosoma mansoni infection and associated risk factors among school children attending primary schools nearby rivers in Jimma town, an urban setting, Southwest Ethiopia | 10.1371/journal.pone.0228007 | Excluded by the title |
| 560 | Kazembe, 2015 | Estimating areas of common risk in low birth weight and infant mortality in Namibia: a joint spatial analysis at sub-regional level | 10.1016/j.sste.2015.02.001 | Excluded by the title |
| 561 | Rasheed, 2021 | Estimating the health burden of aflatoxin attributable stunting among children in low income countries of Africa | 10.1038/s41598-020-80356-4 | Excluded by the title |
| 562 | Kimanya, 2021 | stimating the risk of aflatoxin-induced liver cancer in Tanzania based on biomarker data | 10.1371/journal.pone.0247281 | Excluded by the title |
| 563 | Mayer, 2020 | Estimating the Risk of Human Herpesvirus 6 and Cytomegalovirus Transmission to Ugandan Infants from Viral Shedding in Saliva by Household Contacts | 10.3390/v12020171 | Excluded by the title |
| 564 | Javalkar, 2016 | An estimation of mortality risks among people living with hiv in karnataka state, india: Learnings from an intensive hiv/aids care and support programme |  | Excluded by the title |
| 565 | Bongomin, 2021 } | Estimation of the burden of tinea capitis among children in Africa | 10.1111/myc.13221 | Excluded by the title |
| 566 | Gebreegziabher, 2019 } | Ethiopia's high childhood undernutrition explained: analysis of the prevalence and key correlates based on recent nationally representative data | 10.1017/s1368980019000569 | Excluded by the title |
| 567 | Shaikh, 2020 | Ethnic disparity and exposure to supplements rather than adverse childhood experiences linked to preterm birth in Pakistani women | 10.1016/j.jad.2020.01.180 | Excluded by the title |
| 568 | Howie, 2021 | The Etiology of Childhood Pneumonia in The Gambia: Findings From the Pneumonia Etiology Research for Child Health (PERCH) Study | 10.1097/inf.0000000000002766 | Excluded by the title |
| 569 | Ebruke, 2021 | The Etiology of Pneumonia From Analysis of Lung Aspirate and Pleural Fluid Samples: Findings From the Pneumonia Etiology Research for Child Health (PERCH) Study | 10.1093/cid/ciaa1032 | Excluded by the title |
| 570 | Moore, 2021 | he Etiology of Pneumonia in HIV-1-infected South African Children in the Era of Antiretroviral Treatment: Findings From the Pneumonia Etiology Research for Child Health (PERCH) Study | 10.1097/inf.0000000000002651 | Excluded by the title |
| 571 | Seidenberg, 2021 | The Etiology of Pneumonia in HIV-infected Zambian Children: Findings From the Pneumonia Etiology Research for Child Health (PERCH) Study | 10.1097/inf.0000000000002649 | Excluded by the title |
| 572 | Awori, 2021 | The Etiology of Pneumonia in HIV-uninfected Children in Kilifi, Kenya: Findings From the Pneumonia Etiology Research for Child Health (PERCH) Study | 10.1097/inf.0000000000002653 | Excluded by the title |
| 573 | Moore, 2021 | The Etiology of Pneumonia in HIV-uninfected South African Children: Findings From the Pneumonia Etiology Research for Child Health (PERCH) Study | 10.1097/inf.0000000000002650 | Excluded by the title |
| 574 | Mwananyanda, 2021 | he Etiology of Pneumonia in Zambian Children: Findings From the Pneumonia Etiology Research for Child Health (PERCH) Study | 10.1097/inf.0000000000002652 | Excluded by the title |
| 575 | Buchwald, 2023 | Etiology, Presentation, and Risk Factors for Diarrheal Syndromes in 3 Sub-Saharan African Countries After the Introduction of Rotavirus Vaccines From the Vaccine Impact on Diarrhea in Africa (VIDA) Study | 10.1093/cid/ciad022 | Excluded by the title |
| 576 | Obeagu, 2023 } | EURASIAN EXPERIMENT JOURNAL OF MEDICINE AND MEDICAL SCIENCES (EEJMMS) ISSN: 2992-4103© EEJMMS Publications Volume 4 Issue 1 2023 |  | Excluded by the title |
| 577 | Lule, 2023 | EURASIAN EXPERIMENT JOURNAL OF SCIENTIFIC AND APPLIED RESEARCH (EEJSAR) ISSN: 2992-4146© EEJSAR Publications Volume 4 Issue 1 2023 Factors associated with Adherence to Antiretroviral Treatment among Adolescents Attending Kalisizo |  | Excluded by the title |
| 578 | Cerna-Turoff, 2021 | Factors Associated With Violence Against Children in Low- and Middle-Income Countries: A Systematic Review and Meta-Regression of Nationally Representative Data | 10.1177/1524838020985532 | Excluded by the title |
| 579 | Chhim, 2018 | Factors associated with viral non-suppression among adolescents living with HIV in Cambodia: A cross-sectional study | 10.1186/s12981-018-0205-z | Excluded by the title |
| 580 | Elashi, 2022 | Factors associated with viral suppression among adolescents on antiretroviral therapy in Free State province, South Africa |  | Excluded by the title |
| 581 | Opoku, 2022 | Factors associated with viral suppression and rebound among adult HIV patients on treatment: A retrospective study in Ghana |  | Excluded by the title |
| 582 | Opoku, 2021 | Factors Associated With Viral Suppression and Rebound Among HIV Patients On Treatment: A Retrospective Study in Ghana |  | Excluded by the title |
| 583 | Nabukeera, 2021 | Factors associated with virological non-suppression among HIV-positive children receiving antiretroviral therapy at the Joint Clinical Research Centre in Lubowa, Kampala Uganda | 10.1371/journal.pone.0246140 | Excluded by the title |
| 584 | Ammon, 2018 | Factors impacting antiretroviral therapy adherence among human immunodeficiency virus–positive adolescents in Sub-Saharan Africa: a systematic review |  | Excluded by the title |
| 585 | Nangobi, 2018 | Factors Influencing Adherence To Anti-Retro viral Therapy Among HIV Positive Patients At Bugono Health Center Iv In Iganga District |  | Excluded by the title |
| 586 | Cruz, 2017 | Factors Associated with Stunting among Children Aged 0 to 59 Months from the Central Region of Mozambique |  | Excluded by the title |
| 587 | Pranithram, 2017 | Factors influencing adherence to Anti-retroviral therapy in early and late treatment HIV groups in a teaching hospital, India; a qualitative cross-sectional study |  | Excluded by the title |
| 588 | Mutsigiri-Murewanhema, 2017 | Factors associated with severe malaria among children below ten years in Mutasa and Nyanga districts, Zimbabwe, 2014-2015 |  | Excluded by the title |
| 589 | Habumugisha, 2019 | Factors influencing adherence to antiretroviral therapy among HIV positive adolescents at selected district hospitals' catchment area in Rwanda |  | Excluded by the title |
| 590 | Banagi Yathiraj, 2016 | Factors influencing adherence to antiretroviral therapy among people living with HIV in Coastal South India |  | Excluded by the title |
| 591 | ALABI, 2017 | FACTORS INFLUENCING ADHERENCE TO ANTIRETROVIRAL THERAPY AMONG PEOPLE LIVING WITH HIV/AIDS RECEIVING CARE AT ADEOYO MATERNITY TEACHING HOSPITAL, YEMETU, IBADAN, NIGERIA |  | Excluded by the title |
| 592 | Juma, 2019 | Factors influencing adherence to antiretroviral therapy among the Youth attending Rift valley Provincial General Hospital Nakuru County, Kenya |  | Excluded by the title |
| 591 | Oloi, 2022 | Factors influencing adherence to antiretroviral therapy among youth aged 15-35yrs accessing health care from Ober health center IV, lira city |  | Excluded by the title |
| 592 | Nyalela, 2022 | Factors influencing men’s decisions whether or not to utilize sexual and reproductive health services in low-middle-income countries: a narrative review |  | Excluded by the title |
| 593 | Makhado, 2019 | Factors influencing non-adherence to antiretroviral therapy in South Africa: a systematic review |  | Excluded by the title |
| 594 | Betunga, 2022 | Factors influencing the use of multiple HIV prevention services among Transport workers in a City in Southwestern Uganda |  | Excluded by the title |
| 595 | ADAGI, 2023 | Factors Influencing Virological Suppression Among Hiv Infected Sex Workers and General Populations in Homa Bay County, Kenya |  | Excluded by the title |
| 596 | Mwanguhya, 2017 | Factors that affect adherence to anti-retroviral therapy in HIV positive clients at Comboni Hospital Kyamuhunga Bushenyi Uganda |  | Excluded by the abstract |
| 597 | Adenomon, 2019 | Factors That Enhanced Prevention of Mother-to-Child Transmission of HIV in Nasarawa State of Nigeria Using Logistic, Poisson and Negative Binomial Regression Models |  | Excluded by the title |
| 598 | Laibon, 2017 | Factors that Influence Treatment Adherence among Male Sex Workers on Antiretroviral Therapy in Nairobi City County, Kenya |  | Excluded by the title |
| 599 | Caggiano, 2017 | Factors That Negatively Affect the Prognosis of Pediatric Community-Acquired Pneumonia in District Hospital in Tanzania |  | Excluded by the title |
| 600 | Tomasev, 2021 | Fairness for unobserved characteristics: Insights from technological impacts on queer communities |  | Excluded by the title |
| 601 | YULIYATNI, 2015 | FAKTOR DETERMINAN ORANG DENGAN HIV/AIDS (ODHA) MEMULAI TERAPI ARV PADA PROGRAM TEST AND TREAT DI KLINIK AMERTHA DENPASAR BALI |  | Excluded by the title |
| 602 | Tsegaye, 2017 | Family planning need of people living with HIV/AIDS in antiretroviral therapy clinics of Horro Guduru Wollega zone, Ethiopia | 10.1186/s13104-017-2914-0 | Excluded by the title |
| 603 | Seo, 2019 | The FANMI ("my FAMILY" in Creole) study to evaluate community-based cohort care for adolescent and young women living with HIV in Haiti: Protocol for a randomized controlled trial | 10.1186/s12889-019-8065-6 | Excluded by the title |
| 604 | Nabunya, 2024 | Feasibility and Acceptability of Group-Based Stigma Reduction Interventions for Adolescents Living with HIV and Their Caregivers: The Suubi4Stigma Randomized Clinical Trial (2020–2022) |  | Excluded by the title |
| 605 | Gizaw, 2022 | Fecal indicator bacteria along multiple environmental exposure pathways (water, food, and soil) and intestinal parasites among children in the rural northwest Ethiopia | 10.1186/s12876-022-02174-4 | Excluded by the title |
| 606 | Feyissa, 2020 | Fertility among women living with HIV in western Ethiopia and its implications for prevention of vertical transmission: a cross-sectional study | 10.1136/bmjopen-2019-036391 | Excluded by the title |
| 607 | Abbawa, 2015 | Fertility desire and associated factors among clients on highly active antiretroviral treatment at finoteselam hospital Northwest Ethiopia: A cross sectional study | 10.1186/s12978-015-0063-2 | Excluded by the title |
| 608 | Mekonnen, 2019 | ertility desire and associated factors among HIV positive women attending ART clinics in amhara region referral hospitals in northwest Ethiopia, 2017 | 10.2147/HIV.S221941 | Excluded by the title |
| 609 | Anbesu, 2021 | Fertility desire and associated factors among HIV-positive women attending ART clinics in Afar region, Northeast Ethiopia | 10.5114/hivar.2021.111395 | Excluded by the title |
| 610 | Eniyew, 2022 | Fertility desire and associated factors among people on antiretroviral treatment at a public health facility in Hawassa city, Southern Ethiopia |  | Excluded by the title |
| 611 | Shiferaw, 2019 | Fertility desire and associated factors among women on the reproductive age group of Antiretroviral treatment users in Jimma Town, South West Ethiopia | 10.1186/s13104-019-4190-7 | Excluded by the title |
| 612 | Peltzer, 2018 | Fertility intentions of prenatal and postpartum HIV-positive women in primary care in Mpumalanga province, South Africa: A longitudinal study | 10.2147/HIV.S153212 | Excluded by the title |
| 613 | Thorne, 2018 | he First Norovirus Longitudinal Seroepidemiological Study From Sub-Saharan Africa Reveals High Seroprevalence of Diverse Genotypes Associated With Host Susceptibility Factors | 10.1093/infdis/jiy219 | Excluded by the title |
| 614 | Ayalew, 2016 | First-line antiretroviral treatment failure and associated factors in HIV patients at the University of Gondar Teaching Hospital, Gondar, Northwest Ethiopia |  | Excluded by the title |
| 615 | Biset, 2016 | First-line antiretroviral treatment failure and associated factors in HIV patients at the University of Gondar Teaching Hospital, Gondar, Northwest Ethiopia |  | Excluded by the title |
| 616 | Ramjith, 2021 | Flexible modelling of risk factors on the incidence of pneumonia in young children in South Africa using piece-wise exponential additive mixed modeling | 10.1186/s12874-020-01194-6 | Excluded by the title |
| 617 | Nelima, 2023 | Fluoride distribution in selected foodstuffs from Nakuru County, Kenya, and the risk factors for its human overexposure | 10.1038/s41598-023-41601-8 | Excluded by the title |
| 618 | Thee, 2015 | Fluoroquinolones for the treatment of tuberculosis in children | 10.1016/j.tube.2015.02.037 | Excluded by the title |
| 619 | Davis, 2023 | Food as a Driver of a Cholera Epidemic in Jijiga, Ethiopia-June 2017 | 10.4269/ajtmh.22-0734 | Excluded by the title |
| 620 | Tolasa, 2015 | Food insecurity and associated factors among people living with HIV attending ART Clinic in Fitche Zonal Hospital, Ethiopia |  | Excluded by the title |
| 621 | Umutoniwase, 2022 | ood insecurity and level of depression among patients with chronic diseases, and associated factors during the COVID-19 lockdown: a cross-sectional study in rural Rwanda | 10.1136/bmjopen-2021-054137 | Excluded by the title |
| 622 | Rouhani, 2021 | Food Security among People who Inject Drugs in West Virginia |  | Excluded by the title |
| 623 | Foreman, 2018 | Forecasting life expectancy, years of life lost, and all-cause and cause-specific mortality for 250 causes of death: reference and alternative scenarios for 2016-40 for 195 countries and territories | 10.1016/s0140-6736(18)31694-5 | Excluded by the title |
| 624 | Okuku, 2021 | Forgetfulness and Non-Adherence to Antiretroviral Therapy in Nigeria: A Review |  | Excluded by the title |
| 625 | Jarchi, 2019 | The frequency of HIV-1 infection in iranian children and determination of the transmitted drug resistance in treatment-naïve children |  | Excluded by the title |
| 626 | Mengesha, 2022 | Gashaw Kerebeh1, Yeneneh Ayalew2, Demewoz Kefale1, Ermias Sisay Chanie1, Natnael Moges Misganaw1, Dejen Getaneh Feleke1, Amare Kassaw1, Agimasie Tigabu3, Berihun Bantie3, Mahlet Tamirat2 |  | Excluded by the title |
| 627 | Musa, 2021 | Factors associated with nonadherence to antiretroviral therapy among children with HIV/AIDS in Ahmadu Bello University Teaching Hospital, Zaria, Nigeria |  | Excluded by the title |
| 628 | Robert, 2017 | Gender-based violence and adherence to anti-retroviral therapy among HIV-infected women attending care and treatment clinic, Mbeya Tanzania |  | Excluded by the title |
| 629 | Aamodt, 2015 | Genetic relatedness and risk factor analysis of ampicillin-resistant and high-level gentamicin-resistant enterococci causing bloodstream infections in Tanzanian children | 10.1186/s12879-015-0845-8 | Excluded by the title |
| 630 | Valerie, 2016 | GENETIC STRUCTURE AND GEOGRAPHICAL RELATIONSHIP OF SELECTED COLOCASIA ESCULENTA [L. SCHOTT] GERMPLASM USING SSRS |  | Excluded by the title |
| 631 | Marcon, 2018 | Geno (feno) tipagem e perfil de resistência aos antirretrovirais em pessoas vivendo com HIV no sul de Santa Catarina |  | Excluded by the title |
| 632 | Aoun, 2015 | Geographical accessibility to healthcare and malnutrition in Rwanda | 10.1016/j.socscimed.2015.02.004 | Excluded by the title |
| 633 | Nambuusi, 2019 | Geographical variations of the associations between health interventions and all-cause under-five mortality in Uganda | 10.1186/s12889-019-7636-x | Excluded by the title |
| 634 | Nkwopara, 2019 | Geographically linked risk factors for enrolment into a fast breathing child pneumonia trial in Lilongwe, Malawi: an Innovative Treatments in Pneumonia (ITIP) secondary analysis | E. Nkwopara, R. Schmicker,  10.1136/bmjresp-2019-000414 | Excluded by the title |
| 635 | Endris, 2021 | Geospatial inequality of anaemia among children in Ethiopia | 10.4081/gh.2021.1036 | Excluded by the title |
| 636 | Ejigu, 2020 | Geostatistical analysis and mapping of malaria risk in children of Mozambique | 10.1371/journal.pone.0241680 | Excluded by the title |
| 637 | Yankson, 2019 | Geostatistical analysis and mapping of malaria risk in children under 5 using point-referenced prevalence data in Ghana | 10.1186/s12936-019-2709-y | Excluded by the title |
| 638 | Aheto, 2020 | Geostatistical analysis and mapping: social and environmental determinants of under-five child mortality, evidence from the 2014 Ghana demographic and health survey | 10.1186/s12889-020-09534-3 | Excluded by the title |
| 639 | Nzabakiriraho, 2021 | Geostatistical modeling of malaria prevalence among under-five children in Rwanda | 10.1186/s12889-021-10305-x | Excluded by the title |
| 640 | Ssempiira, 2017 | Geostatistical modelling of malaria indicator survey data to assess the effects of interventions on the geographical distribution of malaria prevalence in children less than 5 years in Uganda | 10.1371/journal.pone.0174948 | Excluded by the title |
| 641 | Beyene, 2023 | Gestational diabetes mellitus and its associated factors in Ethiopia: a systematic review and meta-analysis | 10.1186/s40001-023-01088-5 | Excluded by the title |
| 642 | Dachew, 2021 | Gestational urinary tract infections and the risk of antenatal and postnatal depressive and anxiety symptoms: A longitudinal population-based study | 10.1016/j.jpsychores.2021.110600 | Excluded by the title |
| 643 | Bhutta, 2023 | The global challenge of childhood obesity and its consequences: what can be done? |  | Excluded by the title |
| 644 | Maulsby, 2016 | The global engagement in care convening: Recommended actions to improve health outcomes for people living with HIV |  | Excluded by the title |
| 645 | Kassanjee, 2021 | Global HIV mortality trends among children on antiretroviral treatment corrected for under‐reported deaths: an updated analysis of the International epidemiology Databases to Evaluate AIDS collaboration |  | Excluded by the title |
| 646 | Crichton, 2023 | Global variations in pubertal growth spurts in adolescents living with perinatal HIV | 10.1097/QAD.0000000000003602 | Excluded by the title |
| 647 | Shi, 2017 | Global, regional, and national disease burden estimates of acute lower respiratory infections due to respiratory syncytial virus in young children in 2015: a systematic review and modelling study | 10.1016/s0140-6736(17)30938-8 | Excluded by the title |
| 648 | Zhang, 2023 | Global, Regional, and National Epidemiology of Diabetes in Children From 1990 to 2019 | 10.1001/jamapediatrics.2023.2029 | Excluded by the title |
| 649 | McCulloch, 2017 | Globalization of pediatric transplantation: The risk of tuberculosis or not tuberculosis | 10.1111/petr.12891 | Excluded by the title |
| 650 | Yohannes, 2022 | Glutathione S-transferase gene polymorphisms in association with susceptibility to lead toxicity in lead- and cadmium-exposed children near an abandoned lead-zinc mining area in Kabwe, Zambia | 10.1007/s11356-021-16098-1 | Excluded by the title |
| 651 | Folayan, 2020 | Governance, maternal well-being and early childhood caries in 3-5-year-old children | 10.1186/s12903-020-01149-9 | Excluded by the title |
| 652 | Christensen, 2020 | Governing the manifold subject: a praxiography of Swedish HIV treatment |  | Excluded by the title |
| 653 | Heise, 2016 | Greentree II: Violence against women and girls, and HIV |  | Excluded by the title |
| 654 | Lawn, 2017 | Group B Streptococcal Disease Worldwide for Pregnant Women, Stillbirths, and Children: Why, What, and How to Undertake Estimates? | 10.1093/cid/cix653 | Excluded by the title |
| 655 | Nyatete, 2018 | Group Therapy Model for Torture Survivors: A Case on the Ghosts of 82-IMLU |  | Excluded by the title |
| 656 | Mlilo, 2020 | Growing up and growing old with HIV”: HIV+ adolescents’ experiences of disclosing statuses to romantic partners in Bulawayo, Zimbabwe |  | Excluded by the title |
| 657 | Mwambenu, 2022 | Growth and the pubertal growth spurt in South African adolescents living with perinatally-acquired HIV infection | 10.1371/journal.pone.0262816 | Excluded by the title |
| 658 | Fanelli, 2018 | Guidelines for establishing and operating successful support groups for people living with HIV |  | Excluded by the title |
| 659 | Chanie, 2022 | Half-life time prediction of developing first-line antiretroviral treatment failure and its risk factors among TB and HIV co-infected children in Northwest Ethiopia; multi setting historical follow-up study | 10.1186/s12887-022-03177-6 | Excluded by the title |
| 660 | Pokhrel, 2018 | Harmful alcohol drinking among HIV-positive people in Nepal: an overlooked threat to anti-retroviral therapy adherence and health-related quality of life |  | Excluded by the title |
| 661 | Waiyon, 2021 | Health care providers' perspectives of antiretroviral therapy adherence in Western Highland Province, Papua New Guinea |  | Excluded by the title |
| 662 | Bwakura-Dangarembizi, 2019 | Health Outcomes, Pathogenesis and Epidemiology of Severe Acute Malnutrition (HOPE-SAM): rationale and methods of a longitudinal observational study | 10.1136/bmjopen-2018-023077 | Excluded by the title |
| 663 | ter Haar, 2022 | Health-related quality of life of perinatally HIV-infected young people: a longitudinal study | 10.1080/09540121.2021.1909695 | Excluded by the title |
| 664 | Atukunda, 2019 | Factors Associated with Pregnancy Intentions Amongst Postpartum Women Living with HIV in Rural Southwestern Uganda |  | Excluded by the title |
| 665 | Olivier, 2018 | Healthcare-associated infections in paediatric and neonatal wards: A point prevalence survey at four South African hospitals | 10.7196/SAMJ.2018.v108i5.12862 | Excluded by the title |
| 666 | Lancaster, 2021 | Heavy episodic drinking and HIV disclosure by HIV treatment status among People with HIV in IeDEA Cameroon |  | Excluded by the title |
| 667 | Tran, 2022 | Helicobacter pylori (H. pylori) risk factor analysis and prevalence prediction: a machine learning-based approach | 10.1186/s12879-022-07625-7 | Excluded by the title |
| 668 | Geletaw, 2017 | Hematologic abnormalities and associated factors among HIV infected children pre- and postantiretroviral treatment, North West Ethiopi | 10.2147/JBM.S137067 | Excluded by the title |
| 669 | Tadesse, 2019 | Hepatic and renal toxicity and associated factors among HIV-infected children on antiretroviral therapy: a prospective cohort study | 10.1111/hiv.12693 | Excluded by the title |
| 670 | Enoch, 2019 | Hepatitis A seroprevalence in Western Cape Province, South Africa: Are we in epidemiological transition? | 10.7196/SAMJ.2019.v109i5.13410 | Excluded by the title |
| 671 | Lewnard, 2019 | Heterogeneous susceptibility to rotavirus infection and gastroenteritis in two birth cohort studies: Parameter estimation and epidemiological implications | 10.1371/journal.pcbi.1007014 | Excluded by the title |
| 673 | Fagbamigbe, 2021 | Hierarchical disentanglement of contextual from compositional risk factors of diarrhoea among under-five children in low- and middle-income countries | 10.1038/s41598-021-87889-2 | Excluded by the title |
| 674 | Migisha, 2023 | High blood pressure and associated factors among HIV-infected young persons aged 13 to 25 years at selected health facilities in Rwenzori region, western Uganda, September–October 2021 | DOI: 10.1186/s40885-022-00230-5 | Excluded by the title |
| 675 | Naidu, 2020 | High Burden of Serious Bacterial Infections in African Children Treated for Cancer | 10.1097/inf.0000000000002758 | Excluded by the title |
| 677 | Barffour, 2017 | High Iron Stores in the Low Malaria Season Increase Malaria Risk in the High Transmission Season in a Prospective Cohort of Rural Zambian Children | 10.3945/jn.117.250381 | Excluded by the title |
| 678 | Wondemagegn, 2020 | High level risky sexual behavior among persons living with HIV in the urban setting of the highest HIV prevalent areas in Ethiopia: Implications for interventions | 10.1371/journal.pone.0242701 | Excluded by the title |
| 679 | Murray, 2020 | High levels of viral repression, malnutrition and second-line ART use in adolescents living with HIV: A mixed methods study from Myanmar | 10.1186/s12879-020-04968-x | Excluded by the title |
| 680 | Getachew, 2023 | High prevalence of active trachoma and associated factors among school-aged children in Southwest Ethiopia | 10.1371/journal.pntd.0011846 | Excluded by the title |
| 681 | Kemigisha, 2018 | High prevalence of depressive symptoms among adolescents living with HIV/aids in Uganda |  | Excluded by the title |
| 682 | Innes, 2016 | High Prevalence of Dyslipidemia and Insulin Resistance in HIV-infected Prepubertal African Children on Antiretroviral Therapy | 10.1097/inf.0000000000000927 | Excluded by the title |
| 683 | Yoseph, 2020 | The high prevalence of intestinal parasitic infections is associated with stunting among children aged 6-59 months in Boricha Woreda, Southern Ethiopia: a cross-sectional study | 10.1186/s12889-020-09377-y | Excluded by the title |
| 684 | Wolff, 2020 | igh prevalence of wheeze and atopy in rural Malagasy children | 10.1111/all.14303 | Excluded by the title |
| 685 | Desai, 2022 | High Rates of Viral Suppression and Care Retention among Youth Born Outside of the United States with Perinatally Acquired HIV | 10.1097/INF.0000000000003698 | Excluded by the title |
| 686 | Kim, 2017 | High self-reported non-adherence to antiretroviral therapy amongst adolescents living with HIV in Malawi: Barriers and associated factors | 10.7448/IAS.20.1.21437 | Excluded by the title |
| 687 | Nyakato, 2022 | High Unreported Mortality in Children and Youth (<25 Years) Living With HIV Who Were Lost to Care From Antiretroviral Therapy Programs in Southern Africa: Results From a Multicountry Tracing Study | DOI: 10.1097/QAI.0000000000003090 | Excluded by the title |
| 688 | Shrestha, 2021 | HIV AIDS related knowledge among antiretroviral therapy clients at Kathmandu and Dhulikhel Nepal a cross-sectional study |  | Excluded by the title |
| 689 | Kityo, 2016 | HIV Drug Resistance Among Children Initiating First-Line Antiretroviral Treatment in Uganda | 10.1089/aid.2015.0215 | Excluded by the title |
| 690 | Chia, 2022 | HIV drug resistance in Southeast Asia: prevalence, determinants, and strategic management |  | Excluded by the title |
| 691 | Azamar-Alonso, 2022 | HIV in Mexico: A 10-Year Population-Based Analysis to Evaluate Policy Changes in Diagnosis, Treatment, and Early Mortality on PLWH 2008-2017 |  | Excluded by the title |
| 692 | Cotton, 2020 | HIV outbreak in children in Pakistan: localised or more widespread? | 10.1016/s1473-3099(19)30746-7 | Excluded by the title |
| 693 | Siregar, 2016 | HIV patients drop out in Indonesia: associated factors and potential productivity loss |  | Excluded by the title |
| 694 | Shallo, 2020 | Hiv positive status disclosure and its associated factors among children on antiretroviral therapy in west shoa zone, western ethiopia, 2019: A mixed method cross-sectional study | 10.2147/JMDH.S258851 | Excluded by the title |
| 695 | Lemma, 2022 | HIV Serostatus Disclosure and Its Predictors Among Children Living With HIV in Ethiopia: A Systematic Review and Meta-Analysis | 10.3389/fpubh.2022.859469 | Excluded by the title |
| 696 | Abdulrahman, 2019 | HIV Treatment Adherence-A Shared Burden for Patients, Health-Care Providers, and Other Stakeholders |  | Excluded by the title |
| 697 | Chimbetete, 2018 | HIV-1 drug resistance and third-line therapy outcomes in patients failing second-line therapy in Zimbabwe |  | Excluded by the title |
| 698 | Marjani, 2020 | HIV-1 integrase drug-resistance mutations in Iranian treatment-experienced HIV-1-infected patients |  | Excluded by the title |
| 699 | De E, 2023 | HIV-1 Subtype Diversity and Factors Affecting Drug Resistance among Patients with Virologic Failure in Antiretroviral Therapy in Hainan Province, China, 2014–2020 |  | Excluded by the title |
| 700 | Strehlau, 2016 | HIV-associated neurodevelopmental delay: prevalence, predictors and persistence in relation to antiretroviral therapy initiation and viral suppression | 10.1111/cch.12399 | Excluded by the title |
| 701 | Zash, 2016 | HIV-exposed children account for more than half of 24-month mortality in Botswana | 10.1186/s12887-016-0635-5 | Excluded by the title |
| 702 | Guta, 2020 | HIV-positive status disclosure and associated factors among children in public health facilities in Dire Dawa, Eastern Ethiopia: A cross-sectional study | 10.1371/journal.pone.0239767 | Excluded by the title |
| 703 | Abdool Karim, 2015 | HIV-positive status disclosure in patients in care in rural South Africa: implications for scaling up treatment and prevention interventions |  | Excluded by the title |
| 704 | Chory, 2021 | HIV-Related Knowledge, Attitudes, Behaviors and Experiences of Kenyan Adolescents Living with HIV Revealed in WhatsApp Group Chats | 10.1177/2325958221999579 | Excluded by the title |
| 705 | Etowa, 2020 | HIV-related stigma among black mothers in two north American and one African cities |  | Excluded by the title |
| 706 | Hargreaves, 2016 | HIV-related stigma and universal testing and treatment for HIV prevention and care: design of an implementation science evaluation nested in the HPTN 071 (PopART) cluster-randomized trial in Zambia and South Africa |  | Excluded by the title |
| 708 | Nyalapa, 2019 | IV/AIDS among young women in Malawi: A review of risk factors and interventions |  | Excluded by the title |
| 709 | Tadesse, 2020 | HIV/AIDS Preventive Practice and Associated Factors among Female Sex Workers in Afar Region, Ethiopia: A Community Based Study | 10.4314/ejhs.v30i1.7 | Excluded by the title |
| 710 | Demartoto, 2021 | HIV/AIDS treatment funding system to support the people affected by HIV/AIDS in Surakarta, Indonesia | 10.1080/17290376.2020.1858946 | Excluded by the title |
| 711 | Letuka, 2020 | Household and individual risk factors of anaemia among under-5 children in Lesotho | 10.4314/ahs.v20i3.5 | Excluded by the title |
| 712 | Debele, 2022 | Household food insecurity and physically demanding work during pregnancy are risk factors for low birth weight in north Shewa zone public hospitals, Central Ethiopia, 2021: a multicenter cross-sectional study | 10.1186/s12887-022-03480-2 | Excluded by the title |
| 713 | ABEBE, 2019 | Household food security and associated factors among Adult people living with HIV/AIDS attending ART clinic in Hospitals of Hawassa Town, Southern Ethiopia 2017 |  | Excluded by the title |
| 714 | Tonui, 2018 | Household Food Security and Nutritional Status of Hiv Sero-Positive Clients Attending Longisa County Hospital Comprehensive Care Clinic, Bomet County, Kenya﻿ |  | Excluded by the title |
| 715 | Abuga, 2020 | How Severe Anaemia Might Influence the Risk of Invasive Bacterial Infections in African Children | 10.3390/ijms21186976 | Excluded by the title |
| 716 | SIMANUNGKALIT, 2021 | HUBUNGAN DUKUNGAN KELUARGA TERHADAP KEPATUHAN MINUM OBAT ANTIRETROVIRAL (ARV) PADA ANAK PENDERITA HIV/AIDS DI RSUD Dr. H. ABDUL MOELOEK PROVINSI LAMPUNG |  | Excluded by the title |
| 717 | Mehari, 2021 | Human immune deficiency virus serostatus and associated factors among children of adult index cases in northern Ethiopia | 10.2147/HIV.S267848 | Excluded by the title |
| 718 | Lencha, 2018 | Human immunodeficiency virus infection disclosure status to infected school aged children and associated factors in bale zone, Southeast Ethiopia: Cross sectional study | 10.1186/s12887-018-1336-z | Excluded by the title |
| 719 | Adella, 2023 | Human immunodeficiency virus positive status disclosure among children in northwest Ethiopia: a cross-sectional study | 10.4314/ahs.v23i1.20 | Excluded by the title |
| 720 | Lamas, 2019 | Human-Computer Interaction–INTERACT 2019: 17th IFIP TC 13 International Conference, Paphos, Cyprus, September 2–6, 2019, Proceedings, Part III |  | Excluded by the title |
| 721 | Petermann-Rocha, 2023 | Hygiene Practices and Early Childhood Development in the East Asia-Pacific Region: A Cross-Sectional Analysis | 10.3390/ijerph20042798 | Excluded by the title |
| 722 | Joubert, 2021 | Hypertension among South African children in disadvantaged areas and associations with physical activity, fitness, and cardiovascular risk markers: A cross-sectional study | 10.1080/02640414.2021.1939964 | Excluded by the title |
| 723 | Dachew, 2020 | Hypertensive disorders of pregnancy and the risk of offspring depression in childhood: Findings from the Avon Longitudinal Study of Parents and Children | 10.1017/s0954579419000944 | Excluded by the title |
| 724 | Madrid, 2016 | Hypoglycemia and Risk Factors for Death in 13 Years of Pediatric Admissions in Mozambique | 10.4269/ajtmh.15-0475 | Excluded by the title |
| 725 | Gutin, 2020 | “I did not know about all these”: Perceptions regarding safer conception methods by women living with HIV in Gaborone, Botswana |  | Excluded by the title |
| 726 | Chandra, 2018 | I have no peace of mind—psychosocial distress expressed by rural women living with HIV in India as part of a mobile health intervention—a qualitative study |  | Excluded by the title |
| 727 | Tadmor, 2023 | I won't let you down; why 20% of Men's and Women's Super League players underreported suspected concussions | 10.1016/j.jsams.2023.09.015 | Excluded by the title |
| 728 | Seyoum, 2016 | Identification of different malaria patterns due to Plasmodium falciparum and Plasmodium vivax in Ethiopian children: a prospective cohort study | 10.1186/s12936-016-1253-2 | Excluded by the title |
| 729 | Daniel, 2024 | Identification of risk factors for inpatient mortality in infants and children with severe wasting and/or nutritional oedema-what next? |  | Excluded by the title |
| 730 | Bonawitz, 2016 | Identifying Gaps in Prevention of Mother to Child Transmission of HIV: A Case Series of HIV-positive Infants in Zambia | 10.1097/inf.0000000000001155 |  |
| 731 | Rees, 2020 | Identifying Infants and Young Children at Risk of Unplanned Hospital Admissions and Clinic Visits in Dar es Salaam, Tanzania | 10.1097/inf.0000000000002875 | Excluded by the title |
| 732 | Eckerle, 2022 | Identifying modifiable risk factors for mortality in children aged 1-59 months admitted with WHO-defined severe pneumonia: a single-centre observational cohort study from rural Malawi | 10.1136/bmjpo-2021-001330 | Excluded by the title |
| 733 | Vonaesch, 2018 | Identifying the etiology and pathophysiology underlying stunting and environmental enteropathy: study protocol of the AFRIBIOTA project | 10.1186/s12887-018-1189-5 | Excluded by the title |
| 734 | Nyirenda, 2018 | Immunological bases of increased susceptibility to invasive nontyphoidal Salmonella infection in children with malaria and anaemia | 10.1016/j.micinf.2017.11.014 | Excluded by the title |
| 735 | Vagenas, 2015 | The impact of alcohol use and related disorders on the HIV continuum of care: a systematic review: alcohol and the HIV continuum of care |  | Excluded by the title |
| 736 | Pascom, 2020 | Impact of antiretroviral regimen on viral suppression among pregnant women living with HIV in Brazil |  | Excluded by the title |
| 737 | Papa, 2020 | Impact of ART-induced viral suppression on the HIV epidemic in Italy |  | Excluded by the title |
| 738 | Pretorius, 2020 | Impact of breastfeeding on mortality in sub-Saharan Africa: a systematic review, meta-analysis, and cost-evaluation | 10.1007/s00431-020-03721-5 | Excluded by the title |
| 739 | Macharia, 2021 | The impact of child health interventions and risk factors on child survival in Kenya, 1993-2014: a Bayesian spatio-temporal analysis with counterfactual scenarios | 10.1186/s12916-021-01974-x | Excluded by the title |
| 740 | Tickell, 2017 | Impact of Childhood Nutritional Status on Pathogen Prevalence and Severity of Acute Diarrhea | 10.4269/ajtmh.17-0139 | Excluded by the title |
| 741 | Michielsen, 2020 | he impact of community health workers on HIV therapy outcome in sub-Saharan Africa |  | Excluded by the title |
| 742 | Mayman, 2023 | The impact of COVID-19 on HIV treatment of adolescents in sub-Saharan Africa: A scoping review |  | Excluded by the title |
| 743 | Buckley, 2022 | Impact of Disclosure over Time on the Emotional Well-Being of Children with Perinatally Acquired HIV Infection in South Africa | DOI: 10.1097/DBP.0000000000001008 | Excluded by the title |
| 744 | Wolf, 2018 | Impact of drinking water, sanitation and handwashing with soap on childhood diarrhoeal disease: updated meta-analysis and meta-regression | 10.1111/tmi.13051 | Excluded by the title |
| 745 | Gray, 2020 | mpact of HIV and antiretroviral drug exposure on lung growth and function over 2 years in an African Birth Cohort | 10.1097/qad.0000000000002444 | Excluded by the title |
| 746 | Gray, 2020 | Impact of HIV and antiretroviral drug exposure on lung growth and function over 2 years in an African Birth Cohort | 10.1097/qad.0000000000002444 | Excluded by the title |
| 747 | Izudi, 2023 | Impact of intensive adherence counseling on viral load suppression and mortality among people living with HIV in Kampala, Uganda: a regression discontinuity design |  | Excluded by the title |
| 748 | Edwards, 2015 | The impact of leadership hubs on the uptake of evidence-informed nursing practices and workplace policies for HIV care: a quasi-experimental study in Jamaica, Kenya, Uganda and South Africa |  | Excluded by the title |
| 749 | Sudjaritruk, 2020 | Impact of low-level viraemia on virological failure among Asian children with perinatally acquired HIV on first-line combination antiretroviral treatment: a multicentre, retrospective cohort study | 10.1002/jia2.25550 | Excluded by the title |
| 750 | Asiki, 2016 | The impact of maternal factors on mortality rates among children under the age of five years in a rural Ugandan population between 2002 and 2012 | 10.1111/apa.13252 | Excluded by the title |
| 751 | ABDULRAHMAN, 2015 | IMPACT OF REMINDER MODULE ON ADHERENCE AND TREATMENT OUTCOMES AMONG HIV-POSITIVE PATIENTS ON ANTIRETROVIRAL THERAPY IN HOSPITAL SUNGAI BULOH, MALAYSIA |  | Excluded by the title |
| 752 | Vojnov, 2017 | Impact of SMS/GPRS Printers in Reducing Time to Early Infant Diagnosis Compared With Routine Result Reporting: A Systematic Review and Meta-Analysis | 10.1097/QAI.0000000000001526 | Excluded by the title |
| 753 | Upoalkpajor, 2021 | Impact of Social Media in the Fight Against Misinformation on Corona Virus Pandemic |  | Excluded by the title |
| 754 | Kiwanuka, 2021 | Impact of stigma on HIV treatment seeking behavior among the youth living with HIV and AIDS in sub-Saharan Africa: critical review of literature |  | Excluded by the title |
| 755 | Coombs, 2020 | The impact of stressful life events on antiretroviral treatment adherence and viral load amongst adults in Gugulethu, Cape Town |  | Excluded by the title |
| 756 | Bateganya, 2015 | Impact of support groups for people living with HIV on clinical outcomes: a systematic review of the literature |  | Excluded by the title |
| 757 | Sikkema, 2022 | ImpACT+, a coping intervention to improve clinical outcomes for women living with HIV and sexual trauma in South Africa: study protocol for a randomized controlled trial |  | Excluded by the title |
| 758 | Owili, 2020 | Impacts of discriminated PM(2.5) on global under-five and maternal mortality | 10.1038/s41598-020-74437-7 | Excluded by the title |
| 759 | Ardura-Garcia, 2015 | Implementation and Operational Research: Early Tracing of Children Lost to Follow-Up From Antiretroviral Treatment: True Outcomes and Future RisksS | 10.1097/qai.0000000000000772 | Excluded by the title |
| 760 | McCormick, 2015 | Implementation and Operational Research: Risk Factors of Loss to Follow-up Among HIV-Positive Pediatric Patients in Dar es Salaam, Tanzania | 10.1097/qai.0000000000000782 | Excluded by the title |
| 761 | Molepo, 2018 | Implementation fidelity of HIV care continuum among healthcare workers in Agincourt healthcare facilities |  | Excluded by the title |
| 762 | Costenaro, 2016 | Implementation of the WHO 2011 Recommendations for Isoniazid Preventive Therapy (IPT) in Children Living with HIV/AIDS: A Ugandan Experience | 1097/QAI.0000000000000806 | Excluded by the title |
| 763 | Mosehle, 2020 | Implementing antiretroviral treatment programmes to support employees living with HIV in adherence: the case of companies partnering with the Swedish workplace HIV/AIDS programme (SWHAP) |  | Excluded by the title |
| 764 | Wijaya, 2022 | The Importance Of Nursing Values And Morals In The Care Of Patients With HIV AIDS |  | Excluded by the title |
| 765 | Burman, 2019 | Improved adherence to anti-retroviral therapy among traditionalists: reflections from rural South Africa |  | Excluded by the title |
| 766 | Igweagu, 2019 | Improving Adherence to Anti-retroviral Therapy among Persons Living with HIV/AIDS in Enugu State, South East Nigeria |  | Excluded by the title |
| 767 | Tomlinson, 2016 | Improving early childhood care and development, HIV-testing, treatment and support, and nutrition in Mokhotlong, Lesotho: study protocol for a cluster randomized controlled trial | 10.1186/s13063-016-1658-9 | Excluded by the title |
| 768 | Chimbwete-Phiri, 2020 | Improving HIV/AIDS consultations in Malawi: How interactional sociolinguistics can contribute |  | Excluded by the title |
| 769 | Hejoaka, 2019 | Improving the informed consent process among HIV-infected undisclosed minors participating in a biomedical research: insights from the multicentre nutritional SNACS study in Senegal | 10.1111/tmi.13202 | Excluded by the title |
| 770 | Matare, 2015 | Improving Uptake and Utilization of Nutrition Interventions: A Caregiver Capabilities Perspective |  | Excluded by the title |
| 771 | De Smidt, 2021 | In utero teratogen exposure and cardiometabolic risk in 5-year-old children: a prospective pediatric study | 10.1080/14767058.2019.1692337 | Excluded by the title |
| 772 | Prakash, 2019 | Incentive-driven post-discharge compliance management for chronic disease patients in healthcare service operations |  | Excluded by the title |
| 773 | Duarte, 2022 | Incidence and Clinical Description of Lymphomas in Children and Adolescents with Vertical Transmission of HIV in Rio de Janeiro, Brazil, in Pre- and Post-Combined Antiretroviral Therapy Eras: A Multicentric Hospital-Based Survival Analysis Study | 10.3390/cancers14246129 | Excluded by the title |
| 774 | Bjornstad, 2020 | Incidence and epidemiology of acute kidney injury in a pediatric Malawian trauma cohort: a prospective observational study | 10.1186/s12882-020-01755-3 | Excluded by the title |
| 775 | Belay, 2023 | Incidence and pattern of childhood cancer in Addis Ababa, Ethiopia (2012-2017) | 10.1186/s12885-023-11765-7 | Excluded by the title |
| 776 | Menshw Snr, 2021 | Incidence and predictors of loss to follow-up among children attending art clinics in northeast ethiopia: A retrospective cohort study | DOI: 10.2147/HIV.S320601 | Excluded by the title |
| 777 | Kebede, 2022 | Incidence and predictors of severe acute malnutrition mortality in children aged 6-59 months admitted at Pawe general hospital, Northwest Ethiopia | 10.1371/journal.pone.0263236 | Excluded by the title |
| 778 | Tekese, 2023 | Incidence and predictors of tuberculosis among children receiving antiretroviral therapy in the Wolaita Zone: A retrospective cohort study | DOI: 10.1371/journal.pone.0291502 | Excluded by the title |
| 779 | Temesgen, 2019 | Incidence and predictors of tuberculosis among HIV-positive adults on antiretroviral therapy at Debre Markos referral hospital, Northwest Ethiopia: a retrospective record review |  | Excluded by the title |
| 780 | Beshir, 2019 | Incidence and predictors of tuberculosis among HIV-positive children at Adama Referral Hospital and Medical College, Oromia, Ethiopia: a retrospective follow-up study | 10.4178/epih.e2019028 | Excluded by the title |
| 781 | Kashaf, 2020 | Incidence and progression of trachomatous scarring in a cohort of children in a formerly hyper-endemic district of Tanzania | 10.1371/journal.pntd.0008708 | Excluded by the title |
| 782 | Sahiledengle, 2020 | ncidence and risk factors for hospital-acquired infection among paediatric patients in a teaching hospital: a prospective study in southeast Ethiopia | 10.1136/bmjopen-2020-037997 | Excluded by the title |
| 783 | Devred, 2023 | Incidence and risk factors of neonatal bacterial infections: a community-based cohort from Madagascar (2018-2021) | 10.1186/s12879-023-08642-w | Excluded by the title |
| 784 | Kerebeh, 2022 | Incidence of anemia and predictors among Human Immunodeficiency Virus-infected children on antiretroviral therapy at public health facilities of Bahir Dar City, Northwest Ethiopia: multicenter retrospective follow up study |  | Excluded by the title |
| 785 | Melkamu, 2020 | Incidence of common opportunistic infections among HIV-infected children on ART at Debre Markos referral hospital, Northwest Ethiopia: A retrospective cohort study | 10.1186/s12879-020-4772-y | Excluded by the title |
| 786 | Tebeila, 2021 | Incidence of febrile seizures and associated factors in children in Soweto, South Africa | 10.7196/SAMJ.2021.V111I8.15431 | Excluded by the title |
| 787 | Gaida, 2016 | Incidence of neuropsychiatric side effects of efavirenz in HIV-positive treatment-naïve patients in public-sector clinics in the Eastern Cape |  | Excluded by the title |
| 788 | Mbethe, 2017 | Incidence of refeeding syndrome and its associated factors in South African children hospitalized with severe acute malnutrition | 10.5812/ijp.8297 | Excluded by the title |
| 789 | Temesgen, 2019 | Incidence of tuberculosis among HIV-positive adults on antiretroviral therapy at Debre Markos Referral Hospital, Northwest Ethiopia: A retrospective record review |  | Excluded by the title |
| 790 | Endalamaw, 2018 | Incidence of tuberculosis in children on antiretroviral therapy: A retrospective cohort study | 10.1186/s13104-018-3846-z | Excluded by the title |
| 791 | Denison, 2015 | Incomplete adherence among treatment-experienced adults on antiretroviral therapy in Tanzania, Uganda and Zambia |  | Excluded by the title |
| 792 | Ayele, 2016 | Indirect child mortality estimation technique to identify trends of under-five mortality in Ethiopia | 10.4314/ahs.v16i1.3 | Excluded by the title |
| 793 | Alemayehu, 2020 | Indirect child mortality estimation technique to identify trends of under-five mortality in Ethiopia |  | Excluded by the title |
| 794 | Coulaud, 2022 | Individual and healthcare supply-related HIV transmission factors in HIV-positive patients enrolled in the antiretroviral treatment access program in the Centre and Littoral regions in Cameroon (ANRS-12288 EVOLCam survey) |  | Excluded by the title |
| 795 | Becker, 2020 | Individual, household, and community level barriers to ART adherence among women in rural Eswatini |  | Excluded by the title |
| 796 | Moschovis, 2018 | Individual, maternal and household risk factors for anaemia among young children in sub-Saharan Africa: a cross-sectional study |  | Excluded by the title |
| 797 | PrayGod, 2016 | ndoor Air Pollution and Delayed Measles Vaccination Increase the Risk of Severe Pneumonia in Children: Results from a Case-Control Study in Mwanza, Tanzania | 10.1371/journal.pone.0160804 | Excluded by the title |
| 798 | Kassa Mekonnen, 2022 | Infant feeding practices and its associated factors among HIV positive mothers attending public health institutions at Gondar Town, Northwest Ethiopia, 2019: An institutional based cross-sectional study | 10.1016/j.ijans.2022.100485 | Excluded by the title |
| 799 | Negash, 2019 | Infants and young children feeding practice and associated factors among HIV positive mothers of children 0-23 months in health centers of Gulele sub-city, Addis Ababa, Ethiopia | 10.1186/s13104-019-4729-7 | Excluded by the title |
| 800 | Lubyayi, 2021 | Infection-exposure in infancy is associated with reduced allergy-related disease in later childhood in a Ugandan cohort | 10.7554/eLife.66022 | Excluded by the title |
| 801 | Juma, 2019 | Influence of Contextual Factors on Adherence to ART among the Youth Attending Provincial General Hospital (PGH), Nakuru County, Kenya |  | Excluded by the title |
| 802 | Olajide, | INFLUENCE OF DEMOGRAPHIC AND SOCIO-CULTURAL VARIABLES ON NON-ADHERENCE TO ANTI-RETROVIRAL THERAPY (ART) AMONG HIV/AIDS PATIENTS ATTENDING A GOVERNMENT OWNED HOSPITAL, ONDO STATE, NIGERIA |  | Excluded by the title |
| 803 | Kariuki, 2017 | Influence of Health Literacy on Antiretroviral Treatment Adherence Among HIV/AIDS Infected Adolescents in Thika Level 5 Hospital, Kiambu County |  | Excluded by the title |
| 804 | Pius, 2021 | Influence of intensified adherence counselling on viral load suppression of people receiving antiretroviral therapy at a health centre IV in southwestern Uganda: a qualitative study | 10.1186/s12981-021-00372-w | Excluded by the title |
| 805 | Kimaru, 2021 | The Influence of Neighborhood Characteristics on HIV Treatment Outcomes Among Adults: A Scoping Review Protocol |  | Excluded by the title |
| 806 | Kiarie, 2016 | Influence Of Self-Reported Highly Active Anti Retroviral Therapy Side Effects On Adherence Among Persons With HIV Attending Tigoni District Hospital, Kenya |  | Excluded by the title |
| 807 | Nyazika, 2020 | Influenza-like illness is associated with high pneumococcal carriage density in Malawian children | 10.1016/j.jinf.2020.06.079 | Excluded by the title |
| 808 | Santillán Torres Torija, 2015 | The information and motivation and behavioral skills model of ART adherence among HIV-positive adults in Mexico |  | Excluded by the title |
| 809 | O’Dowd, 2017 | Integrated Care as a Model for Interprofessional Disease Management and the Benefits for People Living with HIV/AIDS |  | Excluded by the title |
| 810 | Avong, 2018 | Integrating community pharmacy into community based anti-retroviral therapy program: A pilot implementation in Abuja, Nigeria |  | Excluded by the title |
| 811 | Kapata, 2017 | Integration of HIV care and family planning: attitudes, challenges and opportunities in Lusaka, Zambia |  | Excluded by the title |
| 812 | Hobson, 2022 | Intersectional HIV and chronic pain stigma: implications for mood, sleep, and pain severity |  | Excluded by the title |
| 813 | Reif, 2020 | Interventions to improve antiretroviral therapy adherence among adolescents and youth in low-and middle-income countries: a systematic review 2015–2019 |  | Excluded by the title |
| 814 | Ndlovu, 2023 } | Interventions to improve young men’s utilisation of HIV-testing services in KwaZulu-Natal, South Africa: perspectives of young men and health care providers |  | Excluded by the title |
| 815 | Mulatu, 2015 | Intestinal parasitic infections among children under five years of age presenting with diarrhoeal diseases to two public health facilities in Hawassa, South Ethiopia | 10.1186/s40249-015-0081-x | Excluded by the title |
| 816 | Otieno, 2023 | Intestinal parasitic infections and risk factors for infection in Kenyan children with and without HIV infection | 10.1016/j.parint.2022.102717 | Excluded by the title |
| 817 | Ferreira, 2020 | Intestinal parasitic infections in children under five in the Central Hospital of Nampula, Northern Mozambique |  | Excluded by the title |
| 818 | Wasihun, 2020 | Intestinal parasitosis, anaemia and risk factors among pre-school children in Tigray region, northern Ethiopia | 10.1186/s12879-020-05101-8 | Excluded by the title |
| 819 | Ferreira, 2020 | Intestinal parasitic infections in children under five in the Central Hospital of Nampula, Northern Mozambique | 10.3855/jidc.11620 | Excluded by the title |
| 820 | Otieno, 2023 } | Intestinal parasitic infections and risk factors for infection in Kenyan children with and without HIV infection | 10.1016/j.parint.2022.102717 | Excluded by the title |
| 821 | Mulatu, 2015 | Intestinal parasitic infections among children under five years of age presenting with diarrhoeal diseases to two public health facilities in Hawassa, South Ethiopia | 10.1186/s40249-015-0081-x | Excluded by the title |
| 822 | Ndlovu, 2023 | Interventions to improve young men’s utilisation of HIV-testing services in KwaZulu-Natal, South Africa: perspectives of young men and health care providers |  | Excluded by the title |
| 823 | Reif, 2020 | Interventions to improve antiretroviral therapy adherence among adolescents and youth in low-and middle-income countries: a systematic review 2015–2019 |  | Excluded by the title |
| 824 | Hobson, 2022 | Intersectional HIV and chronic pain stigma: implications for mood, sleep, and pain severity |  | Excluded by the title |
| 825 | Kapata, 2017 | Integration of HIV care and family planning: attitudes, challenges and opportunities in Lusaka, Zambia |  | Excluded by the title |
| 826 | Avong, 2018 | Integrating community pharmacy into community based anti-retroviral therapy program: A pilot implementation in Abuja, Nigeria |  | Excluded by the title |
| 827 | Beichler, 2023 | Integrated Care as a Model for Interprofessional Disease Management and the Benefits for People Living with HIV/AIDS |  | Excluded by the title |
| 828 | O’Dowd, 2017 | Insomnia and HIV: a biopsychosocial approach |  | Excluded by the title |
| 829 | Santillán Torres Torija, 2015 | The information and motivation and behavioral skills model of ART adherence among HIV-positive adults in Mexico |  | Excluded by the title |
| 830 | Nyazika, 2020 | Influenza-like illness is associated with high pneumococcal carriage density in Malawian children | 10.1016/j.jinf.2020.06.079 | Excluded by the title |
| 831 | Kiarie, 2016 | Influence Of Self-Reported Highly Active Anti Retroviral Therapy Side Effects On Adherence Among Persons With HIV Attending Tigoni District Hospital, Kenya |  | Excluded by the title |
| 832 | Kimaru, 2021 | The Influence of Neighborhood Characteristics on HIV Treatment Outcomes Among Adults: A Scoping Review Protocol |  | Excluded by the title |
| 833 | Pius, 2021 | Influence of intensified adherence counselling on viral load suppression of people receiving antiretroviral therapy at a health centre IV in southwestern Uganda: a qualitative study | 10.1186/s12981-021-00372-w | Excluded by the title |
| 834 | Kariuki, 2017 | Influence of Health Literacy on Antiretroviral Treatment Adherence Among HIV/AIDS Infected Adolescents in Thika Level 5 Hospital, Kiambu County |  | Excluded by the title |
| 835 | Juma, 2019 | Influence of Contextual Factors on Adherence to ART among the Youth Attending Provincial General Hospital (PGH), Nakuru County, Kenya |  | Excluded by the title |
| 836 | Lubyayi, 2021 | Influence of Contextual Factors on Adherence to ART among the Youth Attending Provincial General Hospital (PGH), Nakuru County, Kenya | 10.7554/eLife.66022 | Excluded by the title |
| 837 | Negash, 2019 | Infants and young children feeding practice and associated factors among HIV positive mothers of children 0-23 months in health centers of Gulele sub-city, Addis Ababa, Ethiopia | 10.1186/s13104-019-4729-7 | Excluded by the title |
| 838 | PrayGod, 2016 | Indoor Air Pollution and Delayed Measles Vaccination Increase the Risk of Severe Pneumonia in Children: Results from a Case-Control Study in Mwanza, Tanzania | 10.1371/journal.pone.0160804 | Excluded by the title |
| 839 | Moschovis, 2018 | Individual, maternal and household risk factors for anaemia among young children in sub-Saharan Africa: a cross-sectional study | 10.1136/bmjopen-2017-019654 | Excluded by the title |
| 840 | Becker, 2020 | Individual, household, and community level barriers to ART adherence among women in rural Eswatini |  | Excluded by the title |
| 841 | Coulaud, 2022 | Individual and healthcare supply-related HIV transmission factors in HIV-positive patients enrolled in the antiretroviral treatment access program in the Centre and Littoral regions in Cameroon (ANRS-12288 EVOLCam survey) |  | Excluded by the title |
| 842 | Alemayehu, 2020 | Individual and community-level risk factors in under-five children diarrhea among agro-ecological zones in southwestern Ethiopia | 10.1016/j.ijheh.2019.11344 | Excluded by the title |
| 843 | Ayele, 2016 | Indirect child mortality estimation technique to identify trends of under-five mortality in Ethiopia | 10.4314/ahs.v16i1.3 | Excluded by the title |
| 844 | Denison, 2015 | Incomplete adherence among treatment-experienced adults on antiretroviral therapy in Tanzania, Uganda and Zambia |  | Excluded by the title |
| 845 | Endalamaw, 2018 | Incidence of tuberculosis in children on antiretroviral therapy: A retrospective cohort study |  | Excluded by the title |
| 846 | Temesgen, 2019 | Incidence of tuberculosis among HIV-positive adults on antiretroviral therapy at Debre Markos Referral Hospital, Northwest Ethiopia: A retrospective record review |  | Excluded by the title |
| 847 | Mbethe, 2017 | Incidence of refeeding syndrome and its associated factors in South African children hospitalized with severe acute malnutrition | 10.5812/ijp.8297 | Excluded by the title |
| 848 | Gaida, 2016 | Incidence of neuropsychiatric side effects of efavirenz in HIV-positive treatment-naïve patients in public-sector clinics in the Eastern Cape |  | Excluded by the title |
| 849 | Tebeila, 2021 } | Incidence of febrile seizures and associated factors in children in Soweto, South Africa | 10.7196/SAMJ.2021.V111I8.15431 | Excluded by the title |
| 850 | Melkamu, 2020 | Incidence of common opportunistic infections among HIV-infected children on ART at Debre Markos referral hospital, Northwest Ethiopia: A retrospective cohort study | 10.1186/s12879-020-4772-y | Excluded by the title |
| 851 | Kerebeh, 2022 | ncidence of anemia and predictors among Human Immunodeficiency Virus-infected children on antiretroviral therapy at public health facilities of Bahir Dar City, Northwest Ethiopia: multicenter retrospective follow up study |  | Excluded by the title |
| 852 | Devred, 2023 | Incidence and risk factors of neonatal bacterial infections: a community-based cohort from Madagascar (2018-2021) | 10.1186/s12879-023-08642-w | Excluded by the title |
| 853 | Sahiledengle, 2020 | Incidence and risk factors for hospital-acquired infection among paediatric patients in a teaching hospital: a prospective study in southeast Ethiopia | 10.1136/bmjopen-2020-037997 | Excluded by the title |
| 854 | Kashaf, 2020 | Incidence and progression of trachomatous scarring in a cohort of children in a formerly hyper-endemic district of Tanzania | 10.1371/journal.pntd.0008708 | Excluded by the title |
| 855 | Beshir, 2019 | Incidence and predictors of tuberculosis among HIV-positive children at Adama Referral Hospital and Medical College, Oromia, Ethiopia: a retrospective follow-up study | 10.4178/epih.e2019028 | Excluded by the title |
| 856 | Temesgen, 2019 | Incidence and predictors of tuberculosis among HIV-positive adults on antiretroviral therapy at Debre Markos referral hospital, Northwest Ethiopia: a retrospective record review |  | Excluded by the title |
| 857 | Tekese, 2023 | Incidence and predictors of tuberculosis among children receiving antiretroviral therapy in the Wolaita Zone: A retrospective cohort study | 10.1371/journal.pone.0291502 | Excluded by the title |
| 858 | Kebede, 2022 | Incidence and predictors of severe acute malnutrition mortality in children aged 6-59 months admitted at Pawe general hospital, Northwest Ethiopia | 10.1371/journal.pone.0263236 | Excluded by the title |
| 859 | Menshw Snr, 2021 | Incidence and predictors of loss to follow-up among children attending art clinics in northeast ethiopia: A retrospective cohort study | 10.2147/HIV.S320601 | Excluded by the title |
| 860 | Belay, 2023 | Incidence and pattern of childhood cancer in Addis Ababa, Ethiopia (2012-2017) | 10.1186/s12885-023-11765-7 | Excluded by the title |
| 861 | Bjornstad, 2020 | Incidence and epidemiology of acute kidney injury in a pediatric Malawian trauma cohort: a prospective observational study | 10.1186/s12882-020-01755-3 | Excluded by the title |
| 863 | Duarte, 2022 | Incidence and Clinical Description of Lymphomas in Children and Adolescents with Vertical Transmission of HIV in Rio de Janeiro, Brazil, in Pre- and Post-Combined Antiretroviral Therapy Eras: A Multicentric Hospital-Based Survival Analysis Study | 10.3390/cancers14246129 | Excluded by the title |
| 864 | Prakash, 2019 | Incentive-driven post-discharge compliance management for chronic disease patients in healthcare service operations |  | Excluded by the title |
| 865 | De Smidt, 2021 | In utero teratogen exposure and cardiometabolic risk in 5-year-old children: a prospective pediatric study | 10.1080/14767058.2019.1692337 | Excluded by the title |
| 866 | Matare, 2015 | Improving Uptake and Utilization of Nutrition Interventions: A Caregiver Capabilities Perspective |  | Excluded by the title |
| 867 | Hejoaka, 2019 | Improving the informed consent process among HIV-infected undisclosed minors participating in a biomedical research: insights from the multicentre nutritional SNACS study in Senegal | 10.1111/tmi.13202 | Excluded by the title |
| 868 | Chimbwete-Phiri, 2020 | Improving HIV/AIDS consultations in Malawi: How interactional sociolinguistics can contribute |  | Excluded by the title |
| 869 | Tomlinson, 2016 | Improving early childhood care and development, HIV-testing, treatment and support, and nutrition in Mokhotlong, Lesotho: study protocol for a cluster randomized controlled trial | 10.1186/s13063-016-1658-9 | Excluded by the title |
| 870 | Igweagu, 2019 | mproving Adherence to Anti-retroviral Therapy among Persons Living with HIV/AIDS in Enugu State, South East Nigeria |  | Excluded by the title |
| 871 | Burman, 2019 | Improved adherence to anti-retroviral therapy among traditionalists: reflections from rural South Africa |  | Excluded by the title |
| 872 | Wijaya, 2022 | The Importance Of Nursing Values And Morals In The Care Of Patients With HIV AIDS |  | Excluded by the title |
| 873 | Mosehle, 2020 | Implementing antiretroviral treatment programmes to support employees living with HIV in adherence: the case of companies partnering with the Swedish workplace HIV/AIDS programme (SWHAP) |  | Excluded by the title |
| 874 | Costenaro, 2016 | Implementation of the WHO 2011 Recommendations for Isoniazid Preventive Therapy (IPT) in Children Living with HIV/AIDS: A Ugandan Experience | 10.1097/QAI.0000000000000806 | Excluded by the title |
| 875 | Molepo, 2018 | Implementation fidelity of HIV care continuum among healthcare workers in Agincourt healthcare facilities |  | Excluded by the title |
| 876 | McCormick, 2015 | Implementation and Operational Research: Risk Factors of Loss to Follow-up Among HIV-Positive Pediatric Patients in Dar es Salaam, Tanzania | 10.1097/qai.0000000000000782 | Excluded by the title |
| 877 | Ardura-Garcia, 2015 | Implementation and Operational Research: Early Tracing of Children Lost to Follow-Up From Antiretroviral Treatment: True Outcomes and Future Risks | 10.1097/qai.0000000000000772 | Excluded by the title |
| 878 | Owili, 2020 | Impacts of discriminated PM(2.5) on global under-five and maternal mortality | 10.1038/s41598-020-74437-7 | Excluded by the title |
| 879 | Sikkema, 2022 | mpACT+, a coping intervention to improve clinical outcomes for women living with HIV and sexual trauma in South Africa: study protocol for a randomized controlled trial |  | Excluded by the title |
| 880 | Bateganya, 2015 | Impact of support groups for people living with HIV on clinical outcomes: a systematic review of the literature |  | Excluded by the title |
| 881 | Coombs, 2020 | The impact of stressful life events on antiretroviral treatment adherence and viral load amongst adults in Gugulethu, Cape Town |  | Excluded by the title |
| 882 | Kiwanuka, 2021 | Impact of stigma on HIV treatment seeking behavior among the youth living with HIV and AIDS in sub-Saharan Africa: critical review of literature |  | Excluded by the title |
| 883 | Upoalkpajor, 2021 | Impact of Social Media in the Fight Against Misinformation on Corona Virus Pandemic |  | Excluded by the title |
| 884 | Vojnov, 2017 | Impact of SMS/GPRS Printers in Reducing Time to Early Infant Diagnosis Compared With Routine Result Reporting: A Systematic Review and Meta-Analysis | 10.1097/QAI.0000000000001526 | Excluded by the title |
| 885 | ABDULRAHMAN, 2015 | IMPACT OF REMINDER MODULE ON ADHERENCE AND TREATMENT OUTCOMES AMONG HIV-POSITIVE PATIENTS ON ANTIRETROVIRAL THERAPY IN HOSPITAL SUNGAI BULOH, MALAYSIA |  | Excluded by the title |
| 886 | Asiki, 2016 | The impact of maternal factors on mortality rates among children under the age of five years in a rural Ugandan population between 2002 and 2012 | 10.1111/apa.13252 | Excluded by the title |
| 887 | Sudjaritruk, 2020 | Impact of low-level viraemia on virological failure among Asian children with perinatally acquired HIV on first-line combination antiretroviral treatment: a multicentre, retrospective cohort study | 10.1002/jia2.25550 | Excluded by the title |
| 888 | Edwards, 2015 | The impact of leadership hubs on the uptake of evidence-informed nursing practices and workplace policies for HIV care: a quasi-experimental study in Jamaica, Kenya, Uganda and South Africa |  | Excluded by the title |
| 889 | Izudi, 2023 | Impact of intensive adherence counseling on viral load suppression and mortality among people living with HIV in Kampala, Uganda: a regression discontinuity design |  | Excluded by the title |
| 890 | Gray, 2020 | Impact of HIV and antiretroviral drug exposure on lung growth and function over 2 years in an African Birth Cohort | 10.1097/qad.0000000000002444 | Excluded by the title |
| 891 | Wolf, 2018 | Impact of drinking water, sanitation and handwashing with soap on childhood diarrhoeal disease: updated meta-analysis and meta-regression | 10.1111/tmi.13051 | Excluded by the title |
| 892 | Buckley, 2022 | Impact of Disclosure over Time on the Emotional Well-Being of Children with Perinatally Acquired HIV Infection in South Africa | 10.1097/DBP.0000000000001008 | Excluded by the title |
| 893 | Mayman, 2023 | The impact of COVID-19 on HIV treatment of adolescents in sub-Saharan Africa: A scoping review |  | Excluded by the title |
| 894 | Michielsen, 2020 | The impact of community health workers on HIV therapy outcome in sub-Saharan Africa |  | Excluded by the title |
| 895 | Tickell, 2017 | Impact of Childhood Nutritional Status on Pathogen Prevalence and Severity of Acute Diarrhea | 10.4269/ajtmh.17-0139 | Excluded by the title |
| 896 | Macharia, 2021 | The impact of child health interventions and risk factors on child survival in Kenya, 1993-2014: a Bayesian spatio-temporal analysis with counterfactual scenarios | 10.1186/s12916-021-01974-x | Excluded by the title |
| 897 | Pretorius, 2020 | Impact of breastfeeding on mortality in sub-Saharan Africa: a systematic review, meta-analysis, and cost-evaluation | 10.1007/s00431-020-03721-5 | Excluded by the title |
| 898 | Papa, 2020 | Impact of ART-induced viral suppression on the HIV epidemic in Italy |  | Excluded by the title |
| 899 | Pascom, 2020 | Impact of antiretroviral regimen on viral suppression among pregnant women living with HIV in Brazil |  | Excluded by the title |
| 900 | Vagenas, 2015 | The impact of alcohol use and related disorders on the HIV continuum of care: a systematic review: alcohol and the HIV continuum of care |  | Excluded by the title |
| 901 | Nyirenda, 2018 | Immunological bases of increased susceptibility to invasive nontyphoidal Salmonella infection in children with malaria and anaemia | 10.1016/j.micinf.2017.11.014 | Excluded by the title |
| 902 | Vonaesch, 2018 | Identifying the etiology and pathophysiology underlying stunting and environmental enteropathy: study protocol of the AFRIBIOTA project | 10.1186/s12887-018-1189-5 | Excluded by the title |
| 903 | Deresse, 2016 | Tuberculosis among Ethiopian-born Georgia residents: An ethnographic approach to understand the sociocultural aspects of tuberculosis |  | Excluded by the title |
| 904 | Pascom, 2020 | Impact of antiretroviral regimen on viral suppression among pregnant women living with HIV in Brazil |  | Excluded by the title |
| 905 | Papa, 2020 | Impact of ART-induced viral suppression on the HIV epidemic in Italy |  | Excluded by the title |
| 906 | Pretorius, 2020 | Impact of breastfeeding on mortality in sub-Saharan Africa: a systematic review, meta-analysis, and cost-evaluation | 10.1007/s00431-020-03721-5 | Excluded by the title |
| 907 | Macharia, 2021 | The impact of child health interventions and risk factors on child survival in Kenya, 1993-2014: a Bayesian spatio-temporal analysis with counterfactual scenarios | 10.1186/s12916-021-01974-x | Excluded by the title |
| 908 | Tickell, 2017 | Impact of Childhood Nutritional Status on Pathogen Prevalence and Severity of Acute Diarrhea | 10.4269/ajtmh.17-0139 | Excluded by the title |
| 909 | Michielsen, 2020 | The impact of community health workers on HIV therapy outcome in sub-Saharan Africa |  | Excluded by the title |
| 910 | Mayman, 2023 | The impact of COVID-19 on HIV treatment of adolescents in sub-Saharan Africa: A scoping review |  | Excluded by the title |
| 911 | Buckley, 2022 | Impact of Disclosure over Time on the Emotional Well-Being of Children with Perinatally Acquired HIV Infection in South Africa | 10.1097/DBP.0000000000001008 | Excluded by the title |
| 912 | Wolf, 2018 | Impact of drinking water, sanitation and handwashing with soap on childhood diarrhoeal disease: updated meta-analysis and meta-regression | 10.1111/tmi.13051 | Excluded by the title |
| 913 | Gray, 2020 | Impact of HIV and antiretroviral drug exposure on lung growth and function over 2 years in an African Birth Cohort | 10.1097/qad.0000000000002444 | Excluded by the title |
| 914 | Izudi, 2023 | Impact of intensive adherence counseling on viral load suppression and mortality among people living with HIV in Kampala, Uganda: a regression discontinuity design |  | Excluded by the title |
| 915 | Edwards, 2015 | The impact of leadership hubs on the uptake of evidence-informed nursing practices and workplace policies for HIV care: a quasi-experimental study in Jamaica, Kenya, Uganda and South Africa |  | Excluded by the title |
| 916 | Sudjaritruk, 2020 | Impact of low-level viraemia on virological failure among Asian children with perinatally acquired HIV on first-line combination antiretroviral treatment: a multicentre, retrospective cohort study | 10.1002/jia2.25550 | Excluded by the title |
| 917 | Asiki, 2016 | The impact of maternal factors on mortality rates among children under the age of five years in a rural Ugandan population between 2002 and 2012 | 10.1111/apa.13252 | Excluded by the title |
| 918 | ABDULRAHMAN, 2015 | IMPACT OF REMINDER MODULE ON ADHERENCE AND TREATMENT OUTCOMES AMONG HIV-POSITIVE PATIENTS ON ANTIRETROVIRAL THERAPY IN HOSPITAL SUNGAI BULOH, MALAYSIA |  | Excluded by the title |
| 919 | Vojnov, 2017 | Impact of SMS/GPRS Printers in Reducing Time to Early Infant Diagnosis Compared With Routine Result Reporting: A Systematic Review and Meta-Analysis | 10.1097/QAI.0000000000001526 | Excluded by the title |
| 920 | Upoalkpajor, 2021 | Impact of Social Media in the Fight Against Misinformation on Corona Virus Pandemic |  | Excluded by the title |
| 921 | Kiwanuka, 2021 | Impact of stigma on HIV treatment seeking behavior among the youth living with HIV and AIDS in sub-Saharan Africa: critical review of literature |  | Excluded by the title |
| 922 | Coombs, 2020 | The impact of stressful life events on antiretroviral treatment adherence and viral load amongst adults in Gugulethu, Cape Town |  | Excluded by the title |
| 923 | Bateganya, 2015 | Impact of support groups for people living with HIV on clinical outcomes: a systematic review of the literature |  | Excluded by the title |
| 924 | Sikkema, 2022 | ImpACT+, a coping intervention to improve clinical outcomes for women living with HIV and sexual trauma in South Africa: study protocol for a randomized controlled trial |  | Excluded by the title |
| 925 | Owili, 2020 | Impacts of discriminated PM(2.5) on global under-five and maternal mortality | 10.1038/s41598-020-74437-7 | Excluded by the title |
| 926 | Ardura-Garcia, 2015 | mplementation and Operational Research: Early Tracing of Children Lost to Follow-Up From Antiretroviral Treatment: True Outcomes and Future Risks | 10.1097/qai.0000000000000772 | Excluded by the title |
| 927 | McCormick, 2015 | Implementation and Operational Research: Risk Factors of Loss to Follow-up Among HIV-Positive Pediatric Patients in Dar es Salaam, Tanzania | 10.1097/qai.0000000000000782 | Excluded by the title |
| 928 | Molepo, 2018 | Implementation fidelity of HIV care continuum among healthcare workers in Agincourt healthcare facilities |  | Excluded by the title |
| 929 | Costenaro, 2016 | Implementation of the WHO 2011 Recommendations for Isoniazid Preventive Therapy (IPT) in Children Living with HIV/AIDS: A Ugandan Experience | 10.1097/QAI.0000000000000806 | Excluded by the title |
| 930 | Mosehle, 2020 | Implementing antiretroviral treatment programmes to support employees living with HIV in adherence: the case of companies partnering with the Swedish workplace HIV/AIDS programme (SWHAP) |  | Excluded by the title |
| 931 | Wijaya, 2022 | The Importance Of Nursing Values And Morals In The Care Of Patients With HIV AIDS |  | Excluded by the title |
| 932 | Burman, 2019 | Improved adherence to anti-retroviral therapy among traditionalists: reflections from rural South Africa |  | Excluded by the title |
| 933 | Igweagu, 2019 | Improving Adherence to Anti-retroviral Therapy among Persons Living with HIV/AIDS in Enugu State, South East Nigeria |  | Excluded by the title |
| 934 | Tomlinson, 2016 | Improving early childhood care and development, HIV-testing, treatment and support, and nutrition in Mokhotlong, Lesotho: study protocol for a cluster randomized controlled trial | 10.1186/s13063-016-1658-9 | Excluded by the title |
| 935 | Chimbwete-Phiri, 2020 | Improving HIV/AIDS consultations in Malawi: How interactional sociolinguistics can contribute |  | Excluded by the title |
| 936 | Hejoaka, 2019 | Improving the informed consent process among HIV-infected undisclosed minors participating in a biomedical research: insights from the multicentre nutritional SNACS study in Senegal | 10.1111/tmi.13202 | Excluded by the title |
| 937 | Matare, 2015 | Improving Uptake and Utilization of Nutrition Interventions: A Caregiver Capabilities Perspective |  | Excluded by the title |
| 938 | De Smidt, 2021 | In utero teratogen exposure and cardiometabolic risk in 5-year-old children: a prospective pediatric study | 10.1080/14767058.2019.1692337 | Excluded by the title |
| 939 | Prakash, 2019 | Incentive-driven post-discharge compliance management for chronic disease patients in healthcare service operations |  | Excluded by the title |
| 940 | Duarte, 2022 | Incidence and Clinical Description of Lymphomas in Children and Adolescents with Vertical Transmission of HIV in Rio de Janeiro, Brazil, in Pre- and Post-Combined Antiretroviral Therapy Eras: A Multicentric Hospital-Based Survival Analysis Study | 10.3390/cancers14246129 | Excluded by the title |
| 941 | Bjornstad, 2020 | Incidence and epidemiology of acute kidney injury in a pediatric Malawian trauma cohort: a prospective observational study | 10.1186/s12882-020-01755-3 | Excluded by the title |
| 942 | Belay, 2023 | Incidence and pattern of childhood cancer in Addis Ababa, Ethiopia (2012-2017) | 10.1186/s12885-023-11765-7 | Excluded by the title |
| 943 | Menshw Snr, 2021 | Incidence and predictors of loss to follow-up among children attending art clinics in northeast ethiopia: A retrospective cohort study | 10.2147/HIV.S320601 | Excluded by the title |
| 944 | Kebede, 2022 | Incidence and predictors of severe acute malnutrition mortality in children aged 6-59 months admitted at Pawe general hospital, Northwest Ethiopia | 10.1371/journal.pone.0263236 | Excluded by the title |
| 945 | Tekese, 2023 | Incidence and predictors of tuberculosis among children receiving antiretroviral therapy in the Wolaita Zone: A retrospective cohort study | 10.1371/journal.pone.0291502 | Excluded by the title |
| 946 | Temesgen, 2019 | Incidence and predictors of tuberculosis among HIV-positive adults on antiretroviral therapy at Debre Markos referral hospital, Northwest Ethiopia: a retrospective record review |  | Excluded by the title |
| 947 | Beshir, 2019 | Incidence and predictors of tuberculosis among HIV-positive children at Adama Referral Hospital and Medical College, Oromia, Ethiopia: a retrospective follow-up study | 10.4178/epih.e2019028 | Excluded by the title |
| 948 | Kashaf, 2020 | Incidence and progression of trachomatous scarring in a cohort of children in a formerly hyper-endemic district of Tanzania | 10.1371/journal.pntd.0008708 | Excluded by the title |
| 949 | Sahiledengle, 2020 | Incidence and risk factors for hospital-acquired infection among paediatric patients in a teaching hospital: a prospective study in southeast Ethiopia | 10.1136/bmjopen-2020-037997 | Excluded by the title |
| 950 | Devred, 2023 | Incidence and risk factors of neonatal bacterial infections: a community-based cohort from Madagascar (2018-2021) | 10.1186/s12879-023-08642-w | Excluded by the title |
| 951 | Kerebeh, 2022 | Incidence of anemia and predictors among Human Immunodeficiency Virus-infected children on antiretroviral therapy at public health facilities of Bahir Dar City, Northwest Ethiopia: multicenter retrospective follow up study |  | Excluded by the title |
| 952 | Melkamu, 2020 | Incidence of common opportunistic infections among HIV-infected children on ART at Debre Markos referral hospital, Northwest Ethiopia: A retrospective cohort study | 10.1186/s12879-020-4772-y | Excluded by the title |
| 953 | Tebeila, 2021 | Incidence of febrile seizures and associated factors in children in Soweto, South Africa | 10.7196/SAMJ.2021.V111I8.15431 | Excluded by the title |
| 954 | Gaida, 2016 | Incidence of neuropsychiatric side effects of efavirenz in HIV-positive treatment-naïve patients in public-sector clinics in the Eastern Cape |  | Excluded by the title |
| 955 | Mbethe, 2017 | Incidence of refeeding syndrome and its associated factors in South African children hospitalized with severe acute malnutrition | 10.5812/ijp.8297 | Excluded by the title |
| 956 | Temesgen, 2019 | Incidence of tuberculosis among HIV-positive adults on antiretroviral therapy at Debre Markos Referral Hospital, Northwest Ethiopia: A retrospective record review |  | Excluded by the title |
| 957 | Endalamaw, 2018 | Incidence of tuberculosis in children on antiretroviral therapy: A retrospective cohort study |  | Excluded by the title |
| 958 | Denison, 2015 | Incomplete adherence among treatment-experienced adults on antiretroviral therapy in Tanzania, Uganda and Zambia |  | Excluded by the title |
| 959 | Ayele, 2016 | Indirect child mortality estimation technique to identify trends of under-five mortality in Ethiopia | 10.4314/ahs.v16i1.3 | Excluded by the title |
| 960 | Alemayehu, 2020 | Individual and community-level risk factors in under-five children diarrhea among agro-ecological zones in southwestern Ethiopia | 10.1016/j.ijheh.2019.113447 | Excluded by the title |
| 961 | Coulaud, 2022 | Individual and healthcare supply-related HIV transmission factors in HIV-positive patients enrolled in the antiretroviral treatment access program in the Centre and Littoral regions in Cameroon (ANRS-12288 EVOLCam survey) |  | Excluded by the title |
| 962 | Becker, 2020 | Individual, household, and community level barriers to ART adherence among women in rural Eswatini |  | Excluded by the title |
| 963 | Moschovis, 2018 | Individual, maternal and household risk factors for anaemia among young children in sub-Saharan Africa: a cross-sectional study | 10.1136/bmjopen-2017-019654 | Excluded by the title |
| 964 | PrayGod, 2016 | Indoor Air Pollution and Delayed Measles Vaccination Increase the Risk of Severe Pneumonia in Children: Results from a Case-Control Study in Mwanza, Tanzania | 10.1371/journal.pone.0160804 | Excluded by the title |
| 965 | Kassa Mekonnen, 2022 } | Infant feeding practices and its associated factors among HIV positive mothers attending public health institutions at Gondar Town, Northwest Ethiopia, 2019: An institutional based cross-sectional study | 10.1016/j.ijans.2022.100485 | Excluded by the title |
| 966 | Negash, 2019 | Infants and young children feeding practice and associated factors among HIV positive mothers of children 0-23 months in health centers of Gulele sub-city, Addis Ababa, Ethiopia | 10.1186/s13104-019-4729-7 | Excluded by the title |
| 967 | Lubyayi, 2021 | Infection-exposure in infancy is associated with reduced allergy-related disease in later childhood in a Ugandan cohort | 10.7554/eLife.66022 | Excluded by the title |
| 968 | Juma, 2019 | Influence of Contextual Factors on Adherence to ART among the Youth Attending Provincial General Hospital (PGH), Nakuru County, Kenya |  | Excluded by the title |
| 969 | Kariuki, 2017 | Influence of Health Literacy on Antiretroviral Treatment Adherence Among HIV/AIDS Infected Adolescents in Thika Level 5 Hospital, Kiambu County |  | Excluded by the title |
| 970 | Pius, 2021 | Influence of intensified adherence counselling on viral load suppression of people receiving antiretroviral therapy at a health centre IV in southwestern Uganda: a qualitative study | 10.1186/s12981-021-00372-w | Excluded by the title |
| 971 | Kimaru, 2021 | The Influence of Neighborhood Characteristics on HIV Treatment Outcomes Among Adults: A Scoping Review Protocol |  | Excluded by the title |
| 972 | Kiarie, 2016 | Influence Of Self-Reported Highly Active Anti Retroviral Therapy Side Effects On Adherence Among Persons With HIV Attending Tigoni District Hospital, Kenya |  | Excluded by the title |
| 973 | Nyazika, 2020 | Influenza-like illness is associated with high pneumococcal carriage density in Malawian children | 10.1016/j.jinf.2020.06.079 | Excluded by the title |
| 974 | Santillán Torres Torija, 2015 | The information and motivation and behavioral skills model of ART adherence among HIV-positive adults in Mexico |  | Excluded by the title |
| 975 | O’Dowd, 2017 | Insomnia and HIV: a biopsychosocial approach |  | Excluded by the title |
| 976 | Beichler, 2023 | Integrated Care as a Model for Interprofessional Disease Management and the Benefits for People Living with HIV/AIDS |  | Excluded by the title |
| 977 | Avong, 2018 | Integrating community pharmacy into community based anti-retroviral therapy program: A pilot implementation in Abuja, Nigeria |  | Excluded by the title |
| 978 | Kapata, 2017 | Integration of HIV care and family planning: attitudes, challenges and opportunities in Lusaka, Zambia |  | Excluded by the title |
| 979 | Hobson, 2022 | Intersectional HIV and chronic pain stigma: implications for mood, sleep, and pain severity |  | Excluded by the title |
| 980 | Reif, 2020 | Interventions to improve antiretroviral therapy adherence among adolescents and youth in low-and middle-income countries: a systematic review 2015–2019 |  | Excluded by the title |
| 981 | Ndlovu, 2023 | Interventions to improve young men’s utilisation of HIV-testing services in KwaZulu-Natal, South Africa: perspectives of young men and health care providers |  | Excluded by the title |
| 982 | Mulatu, 2015 | Intestinal parasitic infections among children under five years of age presenting with diarrhoeal diseases to two public health facilities in Hawassa, South Ethiopia | 10.1186/s40249-015-0081-x | Excluded by the title |
| 983 | Otieno, 2023 | Intestinal parasitic infections and risk factors for infection in Kenyan children with and without HIV infection | 10.1016/j.parint.2022.102717 | Excluded by the title |
| 984 | Ferreira, 2020 | Intestinal parasitic infections in children under five in the Central Hospital of Nampula, Northern Mozambique | 10.3855/jidc.11620 | Excluded by the title |
| 985 | Wasihun, 2020 | Intestinal parasitosis, anaemia and risk factors among pre-school children in Tigray region, northern Ethiopia | 10.1186/s12879-020-05101-8 | Excluded by the title |
| 986 | Bauhofer, 2021 | Intestinal protozoa in hospitalized under-five children with diarrhoea in Nampula - a cross-sectional analysis in a low-income setting in northern Mozambique | 10.1186/s12879-021-05881-7 | Excluded by the title |
| 987 | Nalugwa, 2015 | Intestinal schistosomiasis among preschool children along the shores of Lake Victoria in Uganda | 10.1016/j.actatropica.2014.11.014 | Excluded by the title |
| 988 | Kabwama, 2019 | Intimate partner violence among HIV positive women in care - Results from a national survey, Uganda 2016 | 10.1186/s12905-019-0831-1 | Excluded by the title |
| 989 | Getinet, 2022 | Intimate partner violence among reproductive-age women in central Gondar zone, Northwest, Ethiopia: a population-based study | 10.1186/s12905-022-01685-2 | Excluded by the title |
| 990 | McClintock, 2023 | Intimate partner violence and child loss: an evaluation of 7 sub-Saharan African countries | 10.4314/ahs.v23i1.30 | Excluded by the title |
| 991 | Dadabhai, 2024 | Intimate partner violence and excess fertility among women of reproductive age in Malawi | 10.1371/journal.pone.0297959 | Excluded by the title |
| 992 | Hampanda, 2016 | Intimate partner violence and HIV-positive women's non-adherence to antiretroviral medication for the purpose of prevention of mother-to-child transmission in Lusaka, Zambia | 10.1016/j.socscimed.2016.02.011 | Excluded by the title |
| 993 | Imo, 2022 | Intimate partner violence and its association with skilled birth attendance among women in Nigeria: evidence from | 10.1186/s12884-022-04989-1 | Excluded by the title |
| 994 | Byakika-Kibwika, 2017 | Intravenous artesunate plus Artemisnin based Combination Therapy (ACT) or intravenous quinine plus ACT for treatment of severe malaria in Ugandan children: a randomized controlled clinical trial | 10.1186/s12879-017-2924-5 | Excluded by the title |
| 995 | Deloria Knoll, 2021 | Introduction to the Site-specific Etiologic Results From the Pneumonia Etiology Research for Child Health (PERCH) Study | 10.1097/inf.0000000000002778 | Excluded by the title |
| 996 | Jadgal, 2022 | Investigating social support, self-efficacy, and factors affecting adherence to medication in people living with HIV/AIDS: application of IMB model |  | Excluded by the title |
| 997 | Woolley, 2020 | Investigating the Association between Wood and Charcoal Domestic Cooking, Respiratory Symptoms and Acute Respiratory Infections among Children Aged Under 5 Years in Uganda: A Cross-Sectional Analysis of the 2016 Demographic and Health Survey | 10.3390/ijerph17113974 | Excluded by the title |
| 998 | Stein, 2015 | Investigating the psychosocial determinants of child health in Africa: The Drakenstein Child Health Study | 10.1016/j.jneumeth.2015.03.016 | Excluded by the title |
| 999 | Roberts, 2020 | Investigating the spatial variation and risk factors of childhood anaemia in four sub-Saharan African countries | 10.1186/s12889-020-8189-8 | Excluded by the title |
| 1000 | Philip, 2022 | Investigation into Factors Contributing to Adherence with Antiretroviral Regimen of People Living with HIV/AIDs attending Babcock University Teaching Hospital |  | Excluded by the title |
| 1001 | Suklal, 2017 | Investigation of the medication adherence behaviour of private sector patients with communicable and non-communicable diseases |  | Excluded by the title |
| 1002 | Caddick, 2016 | Investing in mental health in low-income countries |  | Excluded by the title |
| 1003 | Muriuki, 2019 | Iron Status and Associated Malaria Risk Among African Children | 10.1093/cid/ciy791 | Excluded by the title |
| 1004 | Gerber, 2022 | Is grip strength linked to body composition and cardiovascular risk markers in primary schoolchildren? Cross-sectional data from three African countries | 10.1136/bmjopen-2021-052326 | Excluded by the title |
| 1005 | Shayo, 2021 | Is the source of domestic water associated with the risk of malaria infection? Spatial variability and a mixed-effects multilevel analysis | 10.1016/j.ijid.2020.12.062 | Excluded by the title |
| 1006 | Nakyanzi, 2024 | “It Soothes Your Heart”: A Multimethod Study Exploring Acceptability of Point-of-Care Viral Load Testing among Ugandan Pregnant and Postpartum Women Living with HIV | 10.3390/diagnostics14010072 | Excluded by the title |
| 1007 | Derose, 2019 | t Was as Though My Spirit Left, Like They Killed Me: The Disruptive Impact of an HIV-Positive Diagnosis among Women in the Dominican Republic |  | Excluded by the title |
| 1008 | Bokoro, 2022 | Joint binary response modelling for childhood comorbidity in Ethiopia | 10.1371/journal.pone.0268040 | Excluded by the title |
| 1009 | Gaston, 2022 | Joint modelling of anaemia and stunting in children less than five years of age in Lesotho: a cross-sectional case study | 10.1186/s12889-022-12690-3 | Excluded by the title |
| 1010 | Adeyemi, 2019 | Joint spatial mapping of childhood anemia and malnutrition in sub-Saharan Africa: a cross-sectional study of small-scale geographical disparities | 10.4314/ahs.v19i3.45 | Excluded by the title |
| 1011 | Rohner, 2016 | Kaposi Sarcoma Risk in HIV-Infected Children and Adolescents on Combination Antiretroviral Therapy From Sub-Saharan Africa, Europe, and Asia | 10.1093/cid/ciw519 | Excluded by the title |
| 1012 | Khulu, 2020 | Key Determinants of Anemia among Youngsters under Five Years in Senegal, Malawi, and Angola | 10.3390/ijerph17228538 | Excluded by the title |
| 1013 | Kayange, 2015 | Kidney disease among children in sub-Saharan Africa: systematic review | 10.1038/pr.2014.189 | Excluded by the title |
| 1014 | Buys, 2016 | Klebsiella pneumoniae bloodstream infections at a South African children's hospital 2006-2011, a cross-sectional study | 10.1186/s12879-016-1919-y | Excluded by the title |
| 1015 | Luba, 2017 | Knowledge about mother-to-child transmission of HIV, its prevention and associated factors among Ethiopian women | 10.7189/jogh.07.020414 | Excluded by the title |
| 1016 | Yanthi, 2024 | Knowledge and Behavior in Human Immunodeficiency Viruses and Reproductive Health of Human Immunodeficiency Viruses-infected Serodiscordant Couples in the Capital City of Indonesia |  | Excluded by the title |
| 1017 | Nalukenge, 2019 | Knowledge and causal attributions for mental disorders in HIV-positive children and adolescents: results from rural and urban Uganda |  | Excluded by the title |
| 1018 | Ntinda, 2022 | Knowledge and Practices of the Fourth Year Degree Nursing Students Regarding Tuberculosis Management at a University in Khomas Region, Namibia |  | Excluded by the title |
| 1019 | Ngwenya, 2020 | Knowledge level on treatment-as-prevention among HIV sero-positive adults on antiretroviral therapy in three health facilities of Lusaka district in Zambia |  | Excluded by the title |
| 1020 | Abtew, 2016 | Knowledge of pregnant women on mother-to-child transmission of HIV, its prevention, and associated factors in Assosa town, northwest Ethiopia | 10.2147/HIV.S100301 | Excluded by the title |
| 1021 | Matlala, 2021 | Knowledge, attitude and perception of university students regarding Medical Male Circumcision at the University of Venda, South Africa |  | Excluded by the title |
| 1022 | Shrestha, 2023 | Knowledge, Attitude, Practice, and Adherence to Antiretroviral Therapy among People Living with HIV in Nepal |  | Excluded by the title |
| 1023 | Mulelu, 2016 | Knowledge, Attitudes and Experiences of PeopleLiving with HIV who are on Antiretroviral Treatment at a Public Health Clinic in Limpopo, South Africa |  | Excluded by the title |
| 1024 | Khumalo, 2017 | Knowledge, attitudes and perceptions of males with regard to medical male circumcision |  | Excluded by the title |
| 1025 | Moxon, 2015 | Laboratory evidence of disseminated intravascular coagulation is associated with a fatal outcome in children with cerebral malaria despite an absence of clinically evident thrombosis or bleeding | 10.1111/jth.13060 | Excluded by the title |
| 1026 | Aramburo, 2018 | Lactate clearance as a prognostic marker of mortality in severely ill febrile children in East Africa | 10.1186/s12916-018-1014-x | Excluded by the title |
| 1027 | Patten, 2018 | Lamivudine monotherapy as a holding regimen for HIV-positive children | 10.1371/journal.pone.0205455 | Excluded by the title |
| 1028 | van Schalkwyk, 2018 | Large Outbreaks of Fungal and Bacterial Bloodstream Infections in a Neonatal Unit, South Africa, 2012-2016 | 10.3201/eid2407.171087 | Excluded by the title |
| 1029 | Omondi, 2023 | Late morning biting behaviour of Anopheles funestus is a risk factor for transmission in schools in Siaya, western Kenya | 10.1186/s12936-023-04806-w | Excluded by the title |
| 1030 | Ryan, 2021 | Lay-delivered talk therapies for adults affected by humanitarian crises in low-and middle-income countries |  | Excluded by the title |
| 1031 | Lepère, 2015 | Length of stay to recover from severe acute malnutrition and associated factors among under-five years children admitted to public hospitals in Aksum, Ethiopia | 10.1371/journal.pone.0238311 | Excluded by the title |
| 1032 | Lodebo, 2017 | Level of adherence and associated factors to option B+ PMTCT among HIV positive pregnant women in Hadiya Zone, Southern Ethiopia |  | Excluded by the title |
| 1033 | Ebuy, 2015 | Level of adherence and predictors of adherence to the Option B+ PMTCT programme in Tigray, northern Ethiopia |  | Excluded by the title |
| 1034 | Asefa, 2020 | Level of good adherence on option b+ pmtctand associated factors among hiv positive pregnant and lactating mothers in public health facilities of ilu abba bor and buno bedele zones, oromia regional state, Southwestern Ethiopia, 2018 | 10.2147/HIV.S283184 | Excluded by the title |
| 1035 | Asgedom, 2024 | Levels of stunting associated factors among under-five children in Ethiopia: A multi-level ordinal logistic regression analysis | 10.1371/journal.pone.0296451 | Excluded by the title |
| 1036 | Lyatuu, 2022 | Lifelong Antiretroviral Treatment for the Prevention of Mother-To-Child Transmission of Hiv in Routine Healthcare in Tanzania, What Works? |  | Excluded by the title |
| 1037 | McNairy, 2015 | The Link4Health study to evaluate the effectiveness of a combination intervention strategy for linkage to and retention in HIV care in Swaziland: protocol for a cluster randomized trial |  | Excluded by the title |
| 1038 | Sobane, 2018 | Literature Review Report: Communication sharing practices and needs of people living with HIV: A case of Nkangala in Mpumalanga and Ekurhuleni in Gauteng |  | Excluded by the title |
| 1039 | Eavanna Maloney, 2023 | The Lived Experience of Adherence to HIV Medication in the Context of Homelessness and Addiction |  | Excluded by the title |
| 1040 | Maloney, 2023 | The lived experience of adherence to HIV medication in the context of homelessness and addiction |  | Excluded by the title |
| 1041 | Ebot, 2015 | Liver function tests of HIV/AIDS patients at the nylon district hospital, Douala, Cameroon |  | Excluded by the title |
| 1042 | Zuma, 2021 | Lives interrupted: navigating hardship during COVID-19 provides lessons in solidarity and visibility for mobile young people in South Africa and Uganda |  | Excluded by the title |
| 1043 | Di Risio, 2016 | Living Optimally with HIV: Youth Experience in a Metropolitan Canadian City |  | Excluded by the title |
| 1044 | Doyal, 2016 | Living with HIV and dying with AIDS: Diversity, inequality and human rights in the global pandemic |  | Excluded by the title |
| 1045 | Addo, 2022 | Living with tuberculosis: a qualitative study of patients’ experiences with disease and treatment |  | Excluded by the title |
| 1046 | Rugnao, 2019 | LLIN Evaluation in Uganda Project (LLINEUP): factors associated with childhood parasitaemia and anaemia 3 years after a national long-lasting insecticidal net distribution campaign: a cross-sectional survey | 10.1186/s12936-019-2838-3 | Excluded by the title |
| 1047 | Moosa, 2018 | Long term adherence to antiretroviral therapy in a South African cohort |  | Excluded by the abstract |
| 1048 | Fenta, 2021 | Long-Term Immunological and Virological Outcomes in Children Receiving Highly Active Antiretroviral Therapy at Hawassa University College of Medicine and Health Sciences, Southern Ethiopia | 10.1155/2021/2498025 | Excluded by the title |
| 1049 | Chandrasekaran, 2018 | Long-term virological outcome in children receiving first-line antiretroviral therapy | 10.1186/s12981-018-0208-9 | Excluded by the title |
| 1050 | Morberg, 2019 | A Longitudinal Analysis of Chlamydial Infection and Trachomatous Inflammation Following Mass Azithromycin Distribution | 10.1080/09286586.2018.1512635 | Excluded by the title |
| 1051 | Olp, 2016 | Longitudinal analysis of the humoral response to Kaposi's sarcoma-associated herpesvirus after primary infection in children | 10.1002/jmv.24546 | Excluded by the title |
| 1052 | Fleece, 2019 | Longitudinal Assessment of Antibiotic Resistance in Fecal Escherichia coli in Tanzanian Children | 10.4269/ajtmh.18-0789 | Excluded by the title |
| 1053 | Gorantla, 2019 | A Longitudinal Study on Adherence to Anti Retroviral Therapy and Its Determinants in Telangana State |  | Excluded by the title |
| 1054 | Matey, 2016 | Lower prevalence of Entamoeba species in children with vertically transmitted HIV infection in Western Kenya | 10.1097/qad.0000000000001002 | Excluded by the title |
| 1055 | Zafar, 2022 | Machine learning-based risk factor analysis and prevalence prediction of intestinal parasitic infections using epidemiological survey data | 10.1371/journal.pntd.0010517 | Excluded by the title |
| 1056 | Mussa, 2022 | Magnitude and associated factors of antiretroviral therapy adherence among children attending HIV care and treatment clinics in Dar es Salaam, Tanzania | 10.1371/journal.pone.0275420 | Excluded by the title |
| 1057 | Tadesse, 2023 | Magnitude and associated factors of low birth weight among term newborns delivered in Addis Ababa public hospitals, Ethiopia, 2021 | 10.1080/01443615.2022.2114332 | Excluded by the title |
| 1058 | Bayleyegn, 2021 } | Magnitude and associated factors of peripheral cytopenia among HIV-infected children attending at University of Gondar Specialized Referral Hospital, Northwest Ethiopia | 10.1371/journal.pone.0247878 | Excluded by the title |
| 1059 | Gelaw, 2021 | Magnitude and associated factors of virological failure among children on ART in Bahir Dar Town public health facilities, Northwest Ethiopia: a facility based cross-sectional study | 10.1186/s13052-021-01030-7 | Excluded by the title |
| 1060 | Alamneh, 2020 | Magnitude and Predictors of Pneumonia among Under-Five Children in Ethiopia: A Systematic Review and Meta-Analysis | 10.1155/2020/1606783 | Excluded by the title |
| 1061 | Tsegaye, 2020 | The magnitude of adherence to option B plus program and associated factors among women in eastern African countries: a systematic review and meta-analysis |  | Excluded by the title |
| 1062 | Fentaw Mulaw, 2020 | Magnitude of Anemia and Associated Factors among HIV-Infected Children Receiving Antiretroviral Therapy in Pastoral Community, Ethiopia: A Retrospective Cross-Sectional Study | 10.1155/2020/9643901 | Excluded by the title |
| 1063 | Asrie, 2020 | Magnitude of anemia and associated factors among human immunodeficiency virus infected children on highly active antiretroviral therapy at university of gondar comprehensive and specialized referral hospital northwest Ethiopia | 10.7754/Clin.Lab.2019.190835 | Excluded by the title |
| 1064 | Tsegay, 2017 | Magnitude of cytopenias among HIV-infected children in Bahir Dar, northwest Ethiopia: A comparison of HAART-naïve and HAART-experienced children |  | Excluded by the title |
| 1065 | Sewale, 2018 | Magnitude of malnutrition and associated factors among HIV infected children attending HIV-care in three public hospitals in East and West Gojjam Zones, Amhara, Northwest, Ethiopia, 2017: a cross-sectional study | DOI: 10.1186/s13104-018-3882-8 | Excluded by the title |
| 1066 | Abera, 2018 | Magnitude of stunting and its determinants in children aged 6-59 months among rural residents of Damot Gale district; southern Ethiopia | 10.1186/s13104-018-3666-1 | Excluded by the title |
| 1067 | Biset, 2022 | Malaria among under-five children in Ethiopia: a systematic review and meta-analysis | 10.1186/s12936-022-04370-9 | Excluded by the title |
| 1068 | Hershey, 2017 | Malaria illness mediated by anaemia lessens cognitive development in younger Ugandan children | 10.1186/s12936-016-1266-x | Excluded by the title |
| 1069 | Hajison, 2018 | Malaria in children under-five: A comparison of risk factors in lakeshore and highland areas, Zomba district, Malawi | 10.1371/journal.pone.0207207 | Excluded by the title |
| 1070 | Gari, 2018 | Malaria increased the risk of stunting and wasting among young children in Ethiopia: Results of a cohort study | 10.1371/journal.pone.0190983 | Excluded by the title |
| 1071 | Mwaiswelo, 2021 | Malaria infection and anemia status in under-five children from Southern Tanzania where seasonal malaria chemoprevention is being implemented | 10.1371/journal.pone.0260785 | Excluded by the title |
| 1072 | Paton, 2021 | Malaria infection and severe disease risks in Africa | 10.1126/science.abj0089 | Excluded by the title |
| 1073 | Mwaba, 2023 | Malaria is the leading cause of acute kidney injury among a Zambian paediatric renal service cohort retrospectively evaluated for aetiologies, predictors of the need for dialysis, and outcomes | 10.1371/journal.pone.0293037 | Excluded by the title |
| 1074 | Debash, 2023 | Malaria surveillance, outbreak investigation, response and its determinant factors in Waghemra Zone, Northeast Ethiopia: unmatched case-control study | 10.1038/s41598-023-36918-3 | Excluded by the title |
| 1075 | Madrid, 2015 | Malaria-associated hypoglycaemia in children | 10.1586/14787210.2015.995632 | Excluded by the title |
| 1076 | Melku, 2018 | Male and undernourished children were at high risk of anemia in Ethiopia: a systematic review and meta-analysis | 10.1186/s13052-018-0513-x | Excluded by the title |
| 1077 | Chibango, 2018 | Male partner involvement in the prevention of mother-to-child transmission (PMTCT) of HIV: a mixed methods study of the Gokwe North District, Zimbabwe |  | Excluded by the title |
| 1078 | Oumer, 2019 | Malnutrition as predictor of survival from anti-retroviral treatment among children living with HIV/AIDS in Southwest Ethiopia: Survival analysis | 10.1186/s12887-019-1823-x | Excluded by the title |
| 1079 | Barnett, 2018 | Maltreatment in childhood and intimate partner violence: A latent class growth analysis in a South African pregnancy cohort | 10.1016/j.chiabu.2018.08.020 | Excluded by the title |
| 1080 | Edwin, 2022 | Management of Hypoplastic Left Heart Syndrome in Low-Resource Settings and the Ethics of Decision-Making | 10.1177/21501351221103511 | Excluded by the title |
| 1081 | Chibango, 2023 | Mapping Evidence of Gender and HIV-Related Health Literacy in Sub-Saharan Africa: A Scoping Review |  | Excluded by the title |
| 1082 | Nwagbara, 2022 | Mapping evidence on factors contributing to maternal and child mortality in sub-Saharan Africa: A scoping review protocol | 10.1371/journal.pone.0272335 | Excluded by the title |
| 1083 | Ndlovu, 2022 | Mapping evidence on the risk factors associated with pediatric cancers in sub-Saharan Africa: a scoping review | 10.1186/s13643-022-01931-6 | Excluded by the title |
| 1084 | Ahmed, 2021 | Mapping Geographical Differences and Examining the Determinants of Childhood Stunting in Ethiopia: A Bayesian Geostatistical Analysis | 10.3390/nu13062104 | Excluded by the title |
| 1085 | Ahmed, 2023 | Mapping Local Variations and the Determinants of Childhood Stunting in Nigeria | 10.3390/ijerph20043250 | Excluded by the title |
| 1086 | Hailu, 2023 | Mapping, trends, and factors associated with anemia among children aged under 5 y in East Africa | 10.1016/j.nut.2023.112202 | Excluded by the title |
| 1087 | Shifa, 2018 | Maternal and child characteristics and health practices affecting under-five mortality: A matched case control study in Gamo Gofa Zone, Southern Ethiopia | 10.1371/journal.pone.0202124 | Excluded by the title |
| 1088 | Kimmie-Dhansay, 2022 | Maternal and infant risk factors and risk indicators associated with early childhood caries in South Africa: a systematic review | 10.1186/s12903-022-02218-x | Excluded by the title |
| 1089 | Osoti, 2023 | Maternal Determinants of Prevention of Mother to Child Transmission of Human Immuno-deficiency Virus among Women in Homa Bay County Referral Hospital, Kenya |  | Excluded by the title |
| 1090 | Ehlert, 2020 | Maternal knowledge and views regarding early hearing detection and intervention in children aged 0-5 years at a semi-urban primary care clinic in South Africa | 10.4102/sajcd.v67i1.681 | Excluded by the title |
| 1091 | Bauserman, 2020 | Maternal mortality in six low and lower-middle income countries from 2010 to 2018: risk factors and trends | 10.1186/s12978-020-00990-z | Excluded by the title |
| 1092 | May, 2016 | Maternal nutritional status as a contributing factor for the risk of fetal alcohol spectrum disorders | 10.1016/j.reprotox.2015.11.006 | Excluded by the title |
| 1093 | Rutayisire, 2023 | Maternal, obstetric and gynecological factors associated with preterm birth in Rwanda: findings from a national longitudinal study | 10.1186/s12884-023-05653-y | Excluded by the title |
| 1094 | Walekhwa, 2021 | Measles outbreak in Western Uganda: a case-control study | 10.1186/s12879-021-06213-5 | Excluded by the title |
| 1095 | Mthethwa, 2017 | Measure of adherence to antiretroviral treatment amongst HIV positive patients attending antiretroviral clinics in selected rural, deep-rural and semi-urban areas of Ugu District in KwaZulu-Natal |  | Excluded by the title |
| 1096 | Ujeneza, 2021 | mechanistic model for long-term immunological outcomes in South African HIV-infected children and adults receiving ART | 10.7554/eLife.42390 | Excluded by the title |
| 1097 | Tiruye, 2020 | The mediation effect of contraceptive use and women's autonomy on the relationship between intimate partner violence and unintended pregnancy in Ethiopia | 10.1186/s12889-020-09514-7 | Excluded by the title |
| 1098 | Muhingi, 2022 | MEDICAL SOCIAL WORK IN KENYA: SCOPE, RELEVANCE, AND UTILITY |  | Excluded by the title |
| 1099 | Dennis, 2022 | Medical, behavioural and social preconception and interconception risk factors among pregnancy planning and recently pregnant Canadian women | 10.1136/fmch-2021-001175 | Excluded by the title |
| 1100 | Aduloju, 2020 | Medication adherence in HIV-positive pregnant women on antiretroviral therapy attending antenatal clinics in Ado metropolis, south-west Nigeria: A multicentre study |  | Excluded by the title |
| 1101 | Rimmele, 2023 | Medication adherence in patients with cluster headache and migraine: an online survey |  | Excluded by the title |
| 1102 | Croome, 2020 | Medication beliefs among people living with HIV taking antiretroviral treatment in Zimbabwe: a qualitative study |  | Excluded by the title |
| 1103 | Matovu, 2021 | Men's comfort in distributing or receiving HIV self-test kits from close male social network members in Dar Es Salaam, Tanzania: baseline results from the STEP project |  | Excluded by the title |
| 1104 | Yi, 2016 | Mental health among men who have sex with men in Cambodia: Implications for integration of mental health services within HIV programmes |  | Excluded by the title |
| 1105 | Smith Fawzi, 2016 | Mental health and antiretroviral adherence among youth living with HIV in Rwanda |  | Excluded by the title |
| 1106 | Wali, 2020 | Factors Associated with Stunting among Children under 5 Years in Five South Asian Countries (2014-2018): Analysis of Demographic Health Surveys | 10.3390/nu12123875 | Excluded by the title |
| 1107 | Altare, 2016 | Factors Associated with Stunting among Pre-school Children in Southern Highlands of Tanzania | 10.1093/tropej/fmw024 | Excluded by the title |
| 1108 | Faris, 2022 | 1.5 billion Muslims fast during Ramadan: The impact on Sustainable Development Goal 3 (health and wellbeing) via bibliometric mapping analysis of literature over seven decades |  | Excluded by the title |
| 1109 | Lilian, 2017 | A 10-year cohort analysis of routine paediatric ART data in a rural South African setting | 10.1017/S0950268816001916 | Excluded by the title |
| 1110 | Valenzuela Sárraga, 2018 | Abordaje de la baja adherencia al tratamiento antirretroviral en personas con virus de inmunodeficiencia humana: revisión sistemática |  | Excluded by the title |
| 1111 | Das, 2016 | Abundance of psychiatric morbidity in perinatally HIV infected children and adolescents with comparison to their HIV negative sibling |  | Excluded by the title |
| 1112 | Tucker, 2021 | Accelerating adolescent HIV research in LMICs: the NICHD HIV Prevention and Treatment through a Comprehensive Care Continuum (PATC3H) Consortium |  | Excluded by the title |
| 1113 | Smith, 2022 | Accuracy of measures for antiretroviral adherence in people living with HIV |  | Excluded by the title |
| 1114 | Cluver, 2016 | Acinetobacter baumannii infections in a South African paediatric intensive care unit |  | Excluded by the title |
| 1115 | Tuke, 2023 | Active Trachoma Prevalence and Related Variables among Children in a Pastoralist Community in Southern Ethiopia in 2021: A Community-Based Cross-Sectional Study |  | Excluded by the title |
| 1116 | Olofin, 2016 | Active Tuberculosis in HIV-Exposed Tanzanian Children up to 2 years of Age: Early-Life Nutrition, Multivitamin Supplementation and Other Potential Risk Factors | 10.1093/tropej/fmv073 | Excluded by the title |
| 1117 | BRONIA, 2020 | ACTORS THAT LEAD TO NON-ADHERENCE TO ANTI-RETROVIRAL THERAPY AMONG HIV INFECTED ADULTS AT CHILENJE FIRST LEVEL HOSPITAL IN LUSAKA DISTRICT |  | Excluded by the title |
| 1118 | Batte, 2022 | Acute kidney injury in hospitalized children with sickle cell anemia | 10.1186/s12882-022-02731-9 | Excluded by the title |
| 1119 | Namazzi, 2022 | Acute Kidney Injury Interacts With Coma, Acidosis, and Impaired Perfusion to Significantly Increase Risk of Death in Children With Severe Malaria | 10.1093/cid/ciac229 | Excluded by the title |
| 1120 | Conroy, 2019 | Acute kidney injury is associated with impaired cognition and chronic kidney disease in a prospective cohort of children with severe malaria | 10.1186/s12916-019-1332-7 | Excluded by the title |
| 1121 | Ovnat Tamir, 2017 | Acute otitis media guidelines in selected developed and developing countries: uniformity and diversity | 10.1136/archdischild-2016-310729 | Excluded by the title |
| 1122 | Zar, 2016 | cute viral bronchiolitis in South Africa: Strategies for management and prevention |  | Excluded by the title |
| 1123 | Le Prevost, 2023 | An adapted algorithm for patient engagement in care for young people living with perinatal HIV in England | 10.1186/s12913-023-10122-5 | Excluded by the title |
| 1124 | Hiregoudar, 2021 | Adherence and Associated Factors to Antiretroviral Therapy among Human Immunodeficiency Virus-Positive Children at Antiretroviral Therapy Centre in South India; A Caretaker’s Report |  | Excluded by the title |
| 1125 | Seetharaman, 2015 | Adherence to Anti Retroviral Therapy of People Living with HIV/AIDS: a Cross Sectional Survey |  | Excluded by the title |
| 1126 | Itoua, 2015 | Adherence to anti-retroviral drugs in pregnant and lactating HIV positive women in Brazzaville |  | Excluded by the title |
| 1127 | Shibabaw, 2018 | Adherence to anti-retroviral therapy among HIV positive pregnant women in Ayder Refferal hospital, Northern Ethiopia |  | Excluded by the title |
| 1128 | Muchena, 2021 | Adherence to anti-retroviral therapy during COVID-19 pandemic among adolescents born HIV-positive |  | Excluded by the title |
| 1129 | Tuychiev, 2021 | Adherence to anti-retroviral therapy in children |  | Excluded by the abstract |
| 1130 | Kambai Avong, 2015 | Adherence to anti-retroviral therapy in north central Nigeria |  | Excluded by the title |
| 1131 | Sekine, 2023 | Adherence to anti-retroviral therapy, decisional conflicts, and health-related quality of life among treatment-naïve individuals living with HIV: a DEARS-J observational study |  | Excluded by the title |
| 1132 | Fonsah, 2017 | Adherence to antiretroviral therapy (ART) in Yaoundé-Cameroon: association with opportunistic infections, depression, ART regimen and side effects |  | Excluded by the title |
| 1133 | Reif, 2020 | Adherence to antiretroviral therapy among adolescents and young adults living with HIV in Haiti: Point-of-care viral load testing to simplify viral load monitoring and improve outcomes |  | Excluded by the title |
| 1134 | Mehta, 2016 | dherence to antiretroviral therapy among children living with HIV in South India |  | Excluded by the title |
| 1135 | Chirundu, 2018 | dherence to antiretroviral therapy among clients utilizing a primary health care facility Kadoma Zimbabwe (2016) |  | Excluded by the abstract |
| 1136 | Chakraborty, 2020 | Adherence to antiretroviral therapy among HIV patients in India: a systematic review and meta-analysis |  | Excluded by the title |
| 1137 | Regmi, 2020 | Adherence to Antiretroviral Therapy among Patient Living with Human Immunodeficiency Virus/Acquired Immune Deficiency Syndrome (PLHA) Patients Visiting at Anti-retroviral Therapy Center in Pokhara |  | Excluded by the abstract |
| 1138 | Ahmad Seyed Alinaghi, 2016 | Adherence to antiretroviral therapy and tuberculosis treatment in a prison of Tehran, Iran |  | Excluded by the title |
| 1139 | Onwunata, 2022 | Adherence to Antiretroviral Therapy by Pregnant Women Accessing Antenatal Clinic at The University of Port Harcourt Teaching Hospital, Tertiary Institution in Rivers State |  | Excluded by the title |
| 1140 | Ali, 2018 | Adherence to antiretroviral therapy in HIV-positive, male intravenous drug users in Pakistan |  | Excluded by the title |
| 1141 | LARGU, 2015 | ADHERENCE TO ANTIRETROVIRAL THERAPY IN HIV/AIDS INFECTED INDIVIDUALS: REASONS FOR NON-ADHERENCE IN THE HIV-POSITIVE POPULATION OF THE NORTHEASTERN OF ROMANIA |  | Excluded by the title |
| 1142 | Ricci, 2016 | Adherence to antiretroviral therapy of Brazilian HIV-infected children and their caregivers |  | Excluded by the title |
| 1143 | Ali, 2018 | Adherence to antiretroviral therapy in HIV-positive, male intravenous drug users in Pakistan |  | Excluded by the title |
| 1144 | LARGU, 2015 | ADHERENCE TO ANTIRETROVIRAL THERAPY IN HIV/AIDS INFECTED INDIVIDUALS: REASONS FOR NON-ADHERENCE IN THE HIV-POSITIVE POPULATION OF THE NORTHEASTERN OF ROMANIA |  | Excluded by the title |
| 1145 | Martelli, 2019 | Adherence to antiretroviral treatment among children and adolescents in Tanzania: Comparison between pill count and viral load outcomes in a rural context of Mwanza region |  | Excluded by the title |
| 1146 | Neupane, 2019 | Adherence to antiretroviral treatment and associated factors among people living with HIV and AIDS in CHITWAN, Nepal |  | Excluded by the title |
| 1147 | Demas, 2022 | Adherence to Antiretroviral Treatment and Associated Factors among Seropositive People Received Treatment in Jimma Town Public Health Facilities, Ethiopia |  | Excluded by the title |
| 1148 | Ehlers, 2015 | Adherence to antiretroviral treatment by adults in a rural area of Botswana |  | Excluded by the title |
| 1149 | Yusuf, 2019 | Adherence to ART among HIV Infected Female Sex Workers in Nigeria |  | Excluded by the title |
| 1150 | Opoka, 2019 | Adherence to clinical guidelines is associated with reduced inpatient mortality among children with severe anemia in Ugandan hospitals  10.1371/journal.pone.0210982 |  | Excluded by the title |
| 1151 | Ejigu, 2020 | Adherence to Highly Active Antiretroviral Therapy Among Children in Ethiopia: A Systematic Review and Meta-analysis |  | Excluded by the title |
| 1152 | Dworkin, 2016 | Adherence to highly active antiretroviral therapy in Hyderabad, India: barriers, facilitators and identification of target groups |  | Excluded by the title |
| 1153 | Zoungrana-Yameogo, 2022 | Adherence to HIV Antiretroviral Therapy Among Pregnant and Breastfeeding Women, Non-Pregnant Women, and Men in Burkina Faso: Nationwide Analysis 2019–2020 |  | Excluded by the title |
| 1154 | Shakya Shrestha, 2020 | Adherence to iron, folic acid and calcium supplement and factors affecting it among the Antenatal care attending women in a tertiary care hospital: A cross sectional study |  | Excluded by the title |
| 1155 | Chukwu, 2019 | Adherence to Medical Treatments Among HIV/AIDS Positive Patients Attending Antiretroviral Therapy Clinics in Nasarawa State, Nigeria |  | Excluded by the title |
| 1156 | Wubneh, 2022 | Adherence to option B+ and its association with disclosure status and counseling among HIV-positive pregnant and lactating women in Ethiopia: systematic review and meta-analysis |  | Excluded by the title |
| 1157 | Fassinou, 2024 | Adherence to option B+ antiretroviral therapy and associated factors in pregnant and breastfeeding women in Sub-Saharan Africa: a systematic review and meta-analysis |  | Excluded by the title |
| 1158 | Barchi, 2019 | Adherence to screening appointments in a cervical cancer clinic serving HIV-positive women in Botswana |  | Excluded by the title |
| 1159 | Takalani, 2019 | Adherence: Perceptions and behaviour of patients on Antiretroviral in Vhembe District of Limpopo Province, South Africa |  | Excluded by the title |
| 1160 | Mpimbaza, 2015 | Admission Risk Score to Predict Inpatient Pediatric Mortality at Four Public Hospitals in Uganda | 10.1371/journal.pone.0133950 | Excluded by the title |
| 1161 | Mhungu, 2023 | Adolescent Girls and Young Women’s Experiences of Living with HIV in the Context of Patriarchal Culture in Sub-Saharan Africa: A Scoping Review |  | Excluded by the title |
| 1162 | Evangeli, 2017 | The Adolescent HIV Disclosure Cognition and Affect Scale: Preliminary Reliability and Validity | 10.1093/jpepsy/jsw107 | Excluded by the title |
| 1163 | Zurbachew, 2023 | Adolescent’s and youth’s adherence to antiretroviral therapy for better treatment outcome and its determinants: multi-center study in public health facilities |  | Excluded by the title |
| 1164 | McCarraher, 2018 | Adolescents living with HIV in the Copperbelt Province of Zambia: Their reproductive health needs and experiences |  | Excluded by the title |
| 1165 | Kadima, 2018 | Adoption of routine virologic testing and predictors of virologic failure among HIV-infected children on antiretroviral treatment in western Kenya | 10.1371/journal.pone.0200242 | Excluded by the title |
| 1166 | Kerrigan, 2015 | Advancing the strategic use of HIV operations research to strengthen local policies and programmes: the Research to Prevention Project |  | Excluded by the title |
| 1167 | Okuku, 2021 | Adverse Drug Effects and Non-Adherence to Antiretroviral Therapy in Nigeria: A Review |  | Excluded by the title |
| 1168 | Nkenfou-Tchinda, 2020 | Adverse Drug Reactions and associated factors among adult HIV-positive patients taking ART at the Yaoundé Central Hospital, Cameroon |  | Excluded by the title |
| 1169 | Mutenda, 2015 | Adverse effects experienced by patients on first line antiretroviral drugs used at Keetmanshoop Hospital (Namibia) |  | Excluded by the title |
| 1170 | de Moura Bubadué, 2019 | dvocacy care on HIV disclosure to children | 10.1111/nin.12278 | Excluded by the title |
| 1171 | Qamar, 2016 | Aeromonas-Associated Diarrhea in Children Under 5 Years: The GEMS Experience | 10.4269/ajtmh.16-0321 | Excluded by the title |
| 1172 | Ndungu, 2021 | Afghan Women's Use of Violence against Their Children and Associations with IPV, Adverse Childhood Experiences and Poverty: A Cross-Sectional and Structural Equation Modelling Analysis | 10.3390/ijerph18157923 | Excluded by the title |
| 1173 | Nalwoga, 2018 | Age of Infection with Kaposi Sarcoma-Associated Herpesvirus and Subsequent Antibody Values Among Children in Uganda | 10.1097/inf.0000000000001909 | Excluded by the title |
| 1174 | Onovo, 2020 | Aggregating loss to follow-up behaviour in people living with HIV on ART: a cluster analysis using unsupervised machine learning algorithm in R |  | Excluded by the title |
| 1175 | Atim, 2018 | Agony resulting from cultural practices of canine bud extraction among children under five years in selected slums of Makindye: a cross sectional study | 10.1186/s12903-018-0599-y | Excluded by the title |
| 1176 | Traisathit, 2018 | AIDS-Defining Events and Deaths in HIV-Infected Children and Adolescents on Antiretrovirals: A 14-Year Study in Thailand | 10.1097/QAI.0000000000001571 | Excluded by the title |
| 1177 | Gebretsadik, 2020 | Alarm Clock-Based reminder for improving low adherence on option B plus antiretroviral therapy among HIV positive pregnant and lactating mothers in Northern Ethiopia |  | Excluded by the title |
| 1178 | Bete, 2023 | Alcohol consumption and associated factors among pregnant women attending antenatal care at governmental hospitals in Harari regional state, Eastern, Ethiopia | 10.1186/s13011-023-00567-6 | Excluded by the title |
| 1179 | Adrawa, 2020 | Alcohol consumption increases non-adherence to ART among people living with HIV enrolled to the community-based care model in rural northern Uganda |  | Excluded by the title |
| 1180 | Mduluza-Jokonya, 2021 | Algorithm for diagnosis of early Schistosoma haematobium using prodromal signs and symptoms in pre-school age children in an endemic district in Zimbabwe | 10.1371/journal.pntd.0009599 | Excluded by the title |
| 1181 | Kiguli, 2015 | Anaemia and blood transfusion in African children presenting to hospital with severe febrile illness | 10.1186/s12916-014-0246-7 | Excluded by the title |
| 1182 | Heinrichs, 2021 | Anaemia and its determinants among young children aged 6-23 months in Ethiopia (2005-2016) | 10.1111/mcn.13082 | Excluded by the title |
| 1183 | Moraleda, 2017 | Anaemia in hospitalised preschool children from a rural area in Mozambique: a case control study in search for aetiological agents | 10.1186/s12887-017-0816-x | Excluded by the title |
| 1184 | Vonaesch, 2021 | Factors Associated with Stunted Growth in Children Under Five Years in Antananarivo, Madagascar and Bangui, Central African Republic | 10.1007/s10995-021-03201-8 | Excluded by the abstract |
| 1185 | {Nopita, 2019 | ANALYSIS OF CST (CASE, SUPPORT AND TREATMENT) AT H ABDUL MANAP HOSPITAL IN JAMBI CITY IN 2018 |  | Excluded by the title |
| 1186 | Nkwopara, 2019 | Analysis of serious adverse events in a paediatric fast breathing pneumonia clinical trial in Malawi | 10.1136/bmjresp-2019-000415 | Excluded by the title |
| 1187 | Ogorenko, 2021 | ANALYSIS OF THE EFFECTIVENESS OF THE INFLUENCE OF COMPLEX TREATMENT ON PSYCHOLOGICAL ADAPTATION IN PATIENTS WITH HUMAN IMMUNODEFFICIENCY VIRUS |  | Excluded by the title |
| 1188 | Tanna, 2016 | Analytical chemistry for assessing medication adherence |  | Excluded by the title |
| 1189 | Chang Cojulun, 2015 | Anemia Among Children Exposed to Polyparasitism in Coastal Kenya | 10.4269/ajtmh.15-0353 | Excluded by the title |
| 1190 | Birhanu, 2018 | Anemia among School-Age Children: Magnitude, Severity and Associated Factors in Pawe Town, Benishangul-Gumuz Region, Northwest Ethiopia | 10.4314/ejhs.v28i3.3 | Excluded by the title |
| 1191 | Ngasala, 2019 | Anemia among Schoolchildren with Malaria and Soil-Transmitted Helminth Coinfections after Repeated Rounds of Mass Drug Administration in Muheza District, Tanzania | 10.4269/ajtmh.19-0362 | Excluded by the title |
| 1192 | Melku, 2018 | Anemia severity among children aged 6-59 months in Gondar town, Ethiopia: a community-based cross-sectional study | 10.1186/s13052-018-0547-0 | Excluded by the title |
| 1193 | Conan, 2017 | Animal-related factors associated with moderate-to-severe diarrhea in children younger than five years in western Kenya: A matched case-control study | 10.1371/journal.pntd.0005795 | Excluded by the title |
| 1194 | Duri, 2023 | Antenatal hepatitis B virus sero-prevalence, risk factors, pregnancy outcomes and vertical transmission rate within 24 months after birth in a high HIV prevalence setting | 10.1186/s12879-023-08523-2 | Excluded by the title |
| 1195 | Kasonka, 2022 | Anthropometry, body composition and chronic disease risk factors among Zambian school-aged children who experienced severe malnutrition in early childhood | 10.1017/s000711452100345 | Excluded by the title |
| 1196 | Salih, 2022 | Anticipated stigma and associated factors among chronic illness patients in Amhara Region Referral Hospitals, Ethiopia: A multicenter cross-sectional study | 10.1371/journal.pone.0273734 | Excluded by the title |
| 1197 | Ameya, 2018 | Antimicrobial susceptibility pattern, and associated factors of Salmonella and Shigella infections among under five children in Arba Minch, South Ethiopia | 10.1186/s12941-018-0253-1 | Excluded by the title |
| 1198 | Storholm, 2019 | Antiretroviral adherence trajectories among Black Americans living with HIV |  | Excluded by the title |
| 1199 | Axelsson, 2015 | Antiretroviral therapy adherence strategies used by patients of a large HIV clinic in Lesotho |  | Excluded by the title |
| 1200 | Mengesha, 2022 | Antiretroviral therapy non-adherence among children living with HIV in Dire Dawa, Eastern Ethiopia: a case-control study | 10.1186/s12887-022-03697-1 | Excluded by the title |
| 1201 | Lwidiko, 2018 | Association between HIV status and depressive symptoms among children and adolescents in the Southern Highlands Zone, Tanzania: A case-control study | 10.1371/journal.pone.0193145 | Excluded by the title |
| 1202 | Ngandu, 2021 | Association Between Household and Maternal Socioeconomic Factors with Birth Outcomes in the Democratic Republic of Congo and South Africa: A Comparative Study | 10.1007/s10995-021-03147-x | Excluded by the title |
| 1203 | Gone, 2017 | The association between malaria and malnutrition among under-five children in Shashogo District, Southern Ethiopia: a case-control study | 10.1186/s40249-016-0221-y | Excluded by the title |
| 1204 | Meyer, 2020 | The association between preoperative anemia and postoperative morbidity in pediatric surgical patients: A secondary analysis of a prospective observational cohort study | 10.1111/pan.13872 | Excluded by the title |
| 1205 | Taiwo, 2017 | Association between Psychological Wellbeing and Antiretroviral Therapy Adherence in North-Central, Nigeria |  | Excluded by the title |
| 1206 | Chikwari, 2017 | Association between self-reported adherence and HIV viral load suppression among older children and adolescents |  | Excluded by the title |
| 1207 | Nutor, 2024 | Association between water insecurity and antiretroviral therapy adherence among pregnant and postpartum women in Greater Accra region of Ghana |  | Excluded by the title |
| 1208 | Nutor, 2024 | Association between water insecurity and antiretroviral therapy adherence among pregnant and postpartum women in Greater Accra region of Ghana. PLOS Glob Public Health 4 (1): e0002747 |  | Excluded by the title |
| 1209 | Zainal-Abidin, 2024 | Association of coping mechanisms with medication adherence among young People living with HIV (PLHIV) in Klang Valley |  | Excluded by the title |
| 1210 | Shapaka, 2023 | Association of full blood count findings with risk of mortality in children with Klebsiella pneumoniae bloodstream infection at a south african children's hospital | 10.1186/s12887-023-04104-z | Excluded by the title |
| 1211 | Ntenda, 2019 | Association of low birth weight with undernutrition in preschool-aged children in Malawi | 10.1186/s12937-019-0477-8 | Excluded by the title |
| 1212 | Masa, 2019 | The Association of Material Hardship with medication adherence and perceived stress among people living with HIV in rural Zambia |  | Excluded by the title |
| 1213 | Forman, 2021 | Association of Respiratory Syncytial Virus Infection and Underlying Risk Factors for Death Among Young Infants Who Died at University Teaching Hospital, Lusaka Zambia | 10.1093/cid/ciab466 | Excluded by the title |
| 1214 | Semba, 2016 | The association of serum choline with linear growth failure in young children from rural Malawi | 10.3945/ajcn.115.129684 | Excluded by the title |
| 1215 | Mpimbaza, 2018 | Associations between erythrocyte polymorphisms and risks of uncomplicated and severe malaria in Ugandan children: A case control study | 10.1371/journal.pone.0203229 | Excluded by the title |
| 1216 | Price, 2018 | Associations between health systems capacity and mother-to-child HIV prevention program outcomes in Zambia | 10.1371/journal.pone.0202889 | Excluded by the title |
| 1217 | Habibi, 2021 | Associations between HIV Status Disclosure, Social Support, and Adherence to and Antiretroviral Therapy in Adults Patients with HIV/AIDS |  | Excluded by the title |
| 1218 | Santri, 2023 | Associations Between Indoor Air Pollutants and Risk Factors for Acute Respiratory Infection Symptoms in Children Under 5: An Analysis of Data From the Indonesia Demographic Health Survey | 10.3961/jpmph.22.470 | Excluded by the title |
| 1219 | Ahmed, 2020 | Associations between infant and young child feeding practices and acute respiratory infection and diarrhoea in Ethiopia: A propensity score matching approach | 10.1371/journal.pone.0230978 | Excluded by the abstract |
| 1220 | Filiatreau, 2021 | Associations between key psychosocial stressors and viral suppression and retention in care among youth with HIV in rural South Africa |  | Excluded by the title |
| 1221 | Feelemyer, 2022 | Associations Between Methamphetamine Use and HIV Viral Load among People Who Inject Drugs in Hai Phong Vietnam: Examining Behavioral vs. Potential Biological Relationships |  | Excluded by the title |
| 1222 | Freeman, 2015 | Associations between school- and household-level water, sanitation and hygiene conditions and soil-transmitted helminth infection among Kenyan school children | 10.1186/s13071-015-1024-x | Excluded by the title |
| 1223 | Gall, 2017 | Associations between selective attention and soil-transmitted helminth infections, socioeconomic status, and physical fitness in disadvantaged children in Port Elizabeth, South Africa: An observational study | 10.1371/journal.pntd.0005573 | Excluded by the title |
| 1224 | Altherr, 2019 | Associations between Water, Sanitation and Hygiene (WASH) and trachoma clustering at aggregate spatial scales, Amhara, Ethiopia | 10.1186/s13071-019-3790-3 | Excluded by the title |
| 1225 | Olaniyan, 2019 | Asthma-related outcomes associated with indoor air pollutants among schoolchildren from four informal settlements in two municipalities in the Western Cape Province of South Africa | 10.1111/ina.12511 | Excluded by the abstract |
| 1226 | Marotta, 2018 | The At Risk Child Clinic (ARCC): 3 Years of Health Activities in Support of the Most Vulnerable Children in Beira, Mozambique | 10.3390/ijerph15071350 | Excluded by the title |
| 1227 | Madiba, 2019 | Attending Informal Preschools and Daycare Centers Is a Risk Factor for Underweight, Stunting and Wasting in Children under the Age of Five Years in Underprivileged Communities in South Africa | 10.3390/ijerph16142589 | Excluded by the title |
| 1228 | Knippler, 2021 | Attitudes Toward Pregnancy Among Women Enrolled in Prevention of Mother-to-Child Transmission of HIV (PMTCT) Services in Moshi, Tanzania | 10.1007/s10461-021-03339-0 | Excluded by the title |
| 1229 | Makenga, 2022 | Attributable risk factors for asymptomatic malaria and anaemia and their association with cognitive and psychomotor functions in schoolchildren of north-eastern Tanzania | 10.1371/journal.pone.0268654 | Excluded by the title |
| 1230 | Mengistu, 2022 | Attrition and associated factors among children living with HIV at a tertiary hospital in Eritrea: A retrospective cohort analysis | 10.1136/bmjpo-2022-001414 | Excluded by the title |
| 1231 | Franz, 2017 | Autism spectrum disorder in sub-saharan africa: A comprehensive scoping review | 10.1002/aur.1766 | Excluded by the title |
| 1232 | Dobaño, 2019 | A Balanced Proinflammatory and Regulatory Cytokine Signature in Young African Children Is Associated With Lower Risk of Clinical Malaria | 10.1093/cid/ciy934 | Excluded by the title |
| 1233 | Antelman, 2021 | Balancing HIV testing efficiency with HIV caseidentification among children and adolescents (2-19 years) using an HIV risk screening approach in Tanzania | 10.1371/journal.pone.0251247 | Excluded by the title |
| 1234 | Buh, 2023 | Barriers and facilitators for interventions to improve ART adherence in Sub-Saharan African countries: A systematic review and meta-analysis |  | Excluded by the title |
| 1235 | Hlophe, 2023 | Barriers and facilitators to anti-retroviral therapy adherence among adolescents aged 10 to 19 years living with HIV in sub-Saharan Africa: A mixed-methods systematic review and meta-analysis |  | Excluded by the title |
| 1236 | Hutahaean, 2023 | Barriers and Facilitators to HIV Treatment Adherence in Indonesia: Perspectives of People Living with HIV and HIV Service Providers |  | Excluded by the title |
| 1237 | Kip, 2022 | Barriers and facilitators to implementing the HEADSS psychosocial screening tool for adolescents living with HIV/AIDS in teen club program in Malawi: health care providers perspectives |  | Excluded by the title |
| 1238 | Chejana, 2021 | Barriers to Adherence to Antiretroviral Medication Among Patients with High Viral Load Compared to Low Viral Load, Mokhotlong, Lesotho |  | Excluded by the title |
| 1239 | Farhoudi, 2018 | Barriers to adherence to antiretroviral treatment among inmates of a prison in Tehran, Iran: A qualitative study |  | Excluded by the title |
| 1240 | Azia, 2016 | Barriers to adherence to antiretroviral treatment in a regional hospital in Vredenburg, Western Cape, South Africa |  | Excluded by the title |
| 1241 | Apondi, 2021 | Barriers to ART adherence among school students living with HIV in Kenya |  | Excluded by the title |
| 1242 | Bassett, 2017 | Barriers to care and 1-year mortality among newly diagnosed HIV-infected people in Durban, South Africa |  | Excluded by the title |
| 1243 | Moucheraud, 2019 | Barriers to HIV treatment adherence: a qualitative study of discrepancies between perceptions of patients and health providers in Tanzania and Uganda |  | Excluded by the title |
| 1244 | Mgosha, 2017 | arriers to switching patients to second-line antiretroviral treatment among clinicians in Tanzania |  | Excluded by the title |
| 1245 | Abiola, 2015 | Baseline adherence, socio-demographic, clinical, immunological, virological and anthropometric characteristics of 242 HIV positive patients on ART in Malaysia |  | Excluded by the title |
| 1246 | Semakula, 2023 | Bayesian belief network modelling approach for predicting and ranking risk factors for malaria infections among children under 5 years in refugee settlements in Uganda | 10.1186/s12936-023-04735-8 | Excluded by the title |
| 1247 | Uwiringiyimana, 2022 | Bayesian geostatistical modelling of stunting in Rwanda: risk factors and spatially explicit residual stunting burden | 10.1186/s12889-022-12552-y | Excluded by the title |
| 1248 | Ibeji, 2022 | Bayesian spatio-temporal modelling and mapping of malaria and anaemia among children between 0 and 59 months in Nigeria | 10.1186/s12936-022-04319-y | Excluded by the title |
| 1249 | Ekstrand, 2020 | A behavioral adherence intervention improves rates of viral suppression among adherence-challenged people living with HIV in South India |  | Excluded by the title |
| 1250 | Zhou, 2023 | Behavioral and emotional difficulties and HIV treatment outcomes among HIV-infected children in rural southwestern China | 10.1186/s13034-023-00601-2 | Excluded by the title |
| 1251 | Derseh, 2021 | Behavioral and environmental determinants of acute diarrhea among under-five children from public health facilities of Siyadebirena Wayu district, north Shoa zone, Amhara regional state, Ethiopia: Unmatched case-control study | 10.1371/journal.pone.0259828 | Excluded by the title |
| 1252 | Cancelliere, 2021 | Being There: Early Career Medical Anthropologists’ Perspectives on Contemporary Challenges in the Field |  | Excluded by the title |
| 1253 | Larkan, 2015 | Between the clinic and the community: Temporality and patterns of ART adherence in the Western Cape Province, South Africa |  | Excluded by the title |
| 1254 | Ssewanyana, 2020 | Beyond Their HIV Status: the Occurrence of Multiple Health Risk Behavior Among Adolescents from a Rural Setting of Sub-Saharan Africa | 10.1007/s12529-020-09877-6 | Excluded by the title |
| 1255 | Fauk, 2023 | Biographical Reinvention: An Asset-Based Approach to Understanding the World of Men Living with HIV in Indonesia | 10.3390/ijerph20166616 | Excluded by the title |
| 1256 | Onyango, 2024 | Biomarker-confirmed suboptimal adherence to isoniazid preventive therapy among children with HIV in western Kenya | 10.1097/QAD.0000000000003719 | Excluded by the title |
| 1257 | Lule, 2019 | Blood pressure risk factors in early adolescents: results from a Ugandan birth cohort | 10.1038/s41371-019-0178-y | Excluded by the title |
| 1258 | Onyango, 2024 | Biomarker-confirmed suboptimal adherence to isoniazid preventive therapy among children with HIV in western Kenya | 10.1097/QAD.0000000000003719 | Excluded by the title |
| 1259 | Onyango, 2024 | Biomarker-confirmed suboptimal adherence to isoniazid preventive therapy among children with HIV in western Kenya | 10.1097/QAD.000000000000371 | Excluded by the title |
| 1260 | Vonaesch, 2021 | Factors Associated with Stunted Growth in Children Under Five Years in Antananarivo, Madagascar and Bangui, Central African Republic | 10.1038/s41371-019-0178-y | Excluded by the title |
| 1261 | Taha, 2024 | Breastfeeding Among Women Living With HIV in the Era of Lifelong ART: An Observational Multicountry Study in Eastern and Southern Africa | 10.1097/QAI.0000000000003306 | Excluded by the title |
| 1262 | Houle, 2019 | Breastfeeding, HIV exposure, childhood obesity, and prehypertension: A South African cohort study | 10.1371/journal.pmed.1002889 | Excluded by the title |
| 1263 | Walakira, 2017 | Building research evidence and collaborative partnerships in Uganda to prevent and protect children against violence: Concerns and priorities for action |  | Excluded by the title |
| 1264 | Houle, 2019 | Breastfeeding, HIV exposure, childhood obesity, and prehypertension: A South African cohort study | 10.1371/journal.pmed.1002889 | Excluded by the title |
| 1265 | Walakira, 2017 | Building research evidence and collaborative partnerships in Uganda to prevent and protect children against violence: Concerns and priorities for action |  | Excluded by the title |
| 1266 | Holden, 2019 | Building resilience to adverse childhood experiences: an assessment of the effects of the stepping stones with children training programme on Tanzanian children affected by HIV and their caregivers |  | Excluded by the title |
| 1267 | Okatch, 2020 | Trends in HIV Treatment Adherence Before and After HIV Status Disclosure to Adolescents in Botswana | 10.1016/j.jadohealth.2020.02.023 | Excluded by the title |
| 1268 | Casale, 2021 | Bullying and ART nonadherence among South African ALHIV: effects, risks, and protective factors |  | Excluded by the title |
| 1269 | Ogweno, 2023 | Burden and risk factors for Schistosoma mansoni infection among primary school children: A quantitative school-based cross-sectional survey in Busega district, Northern Tanzania | 10.1371/journal.pone.0280180 | Excluded by the title |
| 1270 | Tadesse, 2017 | The burden of and risk factors for active trachoma in the North and South Wollo Zones of Amhara Region, Ethiopia: a cross-sectional study | 10.1186/s40249-017-0358-3 | Excluded by the title |
| 1271 | Wagnew, 2019 | Burden of anemia and its association with HAART in HIV infected children in Ethiopia: A systematic review and meta-analysis | 10.1186/s12879-019-4656-1 | Excluded by the title |
| 1272 | Khalil, 2016 | Burden of Diarrhea in the Eastern Mediterranean Region, 1990-2013: Findings from the Global Burden of Disease Study 2013 | 10.4269/ajtmh.16-0339 | Excluded by the title |
| 1273 | Dangor, 2015 | Burden of invasive group B Streptococcus disease and early neurological sequelae in South African infants | 10.1371/journal.pone.0123014 | Excluded by the title |
| 1274 | Gouda, 2019 | Burden of non-communicable diseases in sub-Saharan Africa, 1990-2017: results from the Global Burden of Disease Study 2017 | 109x(19)30374-2 | Excluded by the title |
| 1275 | Kariuki, 2017 | Burden, risk factors, and comorbidities of behavioural and emotional problems in Kenyan children: a population-based study | 10.1016/s2215-0366(16)30403-5 | Excluded by the title |
| 1276 | Chen, 2020 | Campylobacter Colonization, Environmental Enteric Dysfunction, Stunting, and Associated Risk Factors Among Young Children in Rural Ethiopia: A Cross-Sectional Study From the Campylobacter Genomics and Environmental Enteric Dysfunction (CAGED) Project | 10.3389/fpubh.2020.615793 | Excluded by the title |
| 1277 | Karriker-Jaffe, 2023 | Can alcohol policy prevent harms to women and children from men's alcohol consumption? An overview of existing literature and suggested ways forward | 10.1016/j.drugpo.2023.104148 | Excluded by the title |
| 1278 | Semakula, 2017 | Can an educational podcast improve the ability of parents of primary school children to assess the reliability of claims made about the benefits and harms of treatments: study protocol for a randomised controlled trial | 10.1186/s13063-016-1745-y | Excluded by the title |
| 1279 | Nako, 2022 | The capabilities of male migrant miners in preventing and managing HIV: a Lesotho case study |  | Excluded by the title |
| 1280 | van Biljon, 2019 | Cardiac autonomic function and its association with cardiometabolic disease risk factors in Black South African children | 10.1016/j.autneu.2019.03.002 | Excluded by the title |
| 1281 | Namuyonga, 2016 | Cardiac dysfunction among Ugandan HIV-infected children on antiretroviral therapy | 10.1097/INF.0000000000000997 | Excluded by the title |
| 1282 | Garegnani, 2023 | Cardiovascular health metrics in low and middle-income countries: A scoping review | 10.1016/j.ypmed.2023.107534 | Excluded by the title |
| 1283 | Echiru, 2017 | Care Giver Factors Associated With Adherence To Antiretroviral Therapy Among HIV Infected Children At Kisugu Health Centre III |  | Excluded by the title |
| 1284 | Mpimbaza, 2018 | Caregiver responses and association with delayed care-seeking in children with uncomplicated and severe malaria | 10.1186/s12936-018-2630-9 | Excluded by the title |
| 1285 | Lain, 2023 | Caregivers’ psychosocial assessment for identifying HIV-infected infants at risk of poor treatment adherence: an exploratory study in southern Mozambique | 10.1080/09540121.2022.2125159 | Excluded by the title |
| 1286 | Melo, 2018 | Casais heterossexuais sorodiscordantes para o HIV-1: estudo comportamental e de biomarcadores para avaliar práticas sexuais de risco, adesão ao tratamento antirretroviral e transmissão sexual do HIV com outras infecções sexualmente transmissíveis |  | Excluded by the title |
| 1287 | Farhoudi, 2022 | Cascade of care in people living with HIV in Iran in 2019; how far to reach UNAIDS/WHO targets |  | Excluded by the title |
| 1288 | De Neve, 2018 | Causal Effect of Parental Schooling on Early Childhood Undernutrition: Quasi-Experimental Evidence From Zimbabwe | 10.1093/aje/kwx195 | Excluded by the title |
| 1289 | Ragwar, 2023 | Causal factors of childhood pneumonia high mortalities and the impact of community case management on child survival in Sub-Saharan Africa: a systematic review | 10.1016/j.puhe.2023.07.033 | Excluded by the title |
| 1290 | Umeta, 2021 | Causes and predictors of hospitalization and in-hospital mortality among HIV/AIDS patients on highly active antiretroviral therapy in secondary and tertiary care hospitals in Oromia Regional State: multi-center cross-sectional study |  | Excluded by the title |
| 1291 | Gupta, 2018 | Causes of death and predictors of childhood mortality in Rwanda: a matched case-control study using verbal social autopsy | 10.1186/s12889-018-6282-z | Excluded by the title |
| 1292 | Nasuuna, 2019 | Challenges faced by caregivers of virally non-suppressed children on the intensive adherence counselling program in Uganda: A qualitative study | 10.1186/s12913-019-3963-y | Excluded by the title |
| 1293 | Zar, 2020 | Challenges of COVID-19 in children in low- and middle-income countries | 10.1016/j.prrv.2020.06.016 | Excluded by the title |
| 1294 | Kakkar, 2020 | Challenges to achieving and maintaining viral suppression among children living with HIV | 10.1097/QAD.0000000000002454 | Excluded by the title |
| 1295 | van Wyk, 2019 | Challenges to HIV treatment adherence amongst adolescents in a low socio-economic setting in Cape Town |  | Excluded by the title |
| 1296 | Noiman, 2017 | CHANGES in adherence and program retention and associated factors among hiv-infected women receiving option b+ for preventing mother-to-child transmission of hiv in Kampala, Uganda: a mixed methods approach |  | Excluded by the title |
| 1297 | Ngari, 2018 | Changes in susceptibility to life-threatening infections after treatment for complicated severe malnutrition in Kenya | 10.1093/ajcn/nqy007 | Excluded by the title |
| 1298 | Magomere, 2019 | Characterization of HIV drug resistance mutations and subtype diversity of isolates from children and adolescents failing viral suppression in Kenyatta national hospital |  | Excluded by the title |
| 1299 | Nguyen, 2023 | Characterizing the Development of Research Landscapes in Substance Use and HIV/AIDS During 1990 to 2021 |  | Excluded by the title |
| 1300 | Tsondai, 2020 | Characterizing the double‐sided cascade of care for adolescents living with HIV transitioning to adulthood across Southern Africa | 10.1002/jia2.25447 | Excluded by the title |
| 1301 | DeAtley, 2021 | The child ecosystem and childhood pulmonary tuberculosis: A South African perspective | 10.1002/ppul.25369 | Excluded by the title |
| 1302 | Semba, 2016 | Child Stunting is Associated with Low Circulating Essential Amino Acids | 10.1016/j.ebiom.2016.02.030 | Excluded by the title |
| 1303 | Namirembe, 2022 | Child stunting starts in utero: Growth trajectories and determinants in Ugandan infants | 10.1111/mcn.13359 | Excluded by the title |
| 1304 | Titi, 2018 | Child understandings of the causation of childhood burn injuries: Child activity, parental domestic demands, and impoverished settings | 10.1111/cch.12484 | Excluded by the title |
| 1305 | Altare, 2016 | Child Wasting in Emergency Pockets: A Meta-Analysis of Small-Scale Surveys from Ethiopia | 10.3390/ijerph13020178 | Excluded by the title |
| 1306 | Mortier, 2022 | Childhood adversities and suicidal thoughts and behaviors among first-year college students: results from the WMH-ICS initiative | 10.1007/s00127-021-02151-4 | Excluded by the title |
| 1307 | Champion, 2022 | Childhood blood lead levels and environmental risk factors in Madagascar |  | Excluded by the title |
| 1308 | Le Roux, 2024 | Childhood deaths due to pneumonia: a novel causal analysis of aetiology |  | Excluded by the title |
| 1309 | Gizaw, 2018 | Childhood intestinal parasitic infection and sanitation predictors in rural Dembiya, northwest Ethiopia | 10.1186/s12199-018-0714-3 | Excluded by the abstract |
| 1310 | Le Roux, 2024 | Childhood deaths due to pneumonia: a novel causal analysis of aetiology | 10.1016/s2352-4642(24)00015-4 | Excluded by the title |
| 1311 | Gizaw, 2018 | Childhood intestinal parasitic infection and sanitation predictors in rural Dembiya, northwest Ethiopia | 10.1186/s12199-018-0714-3 | Excluded by the title |
| 1312 | Marangu, 2019 | Childhood pneumonia in low-and-middle-income countries: An update | 10.1016/j.prrv.2019.06.001 | Excluded by the title |
| 1313 | Sema, 2021 | Childhood stunting and associated factors among irrigation and non-irrigation user northwest, Ethiopia: a comparative cross-sectional study |  | Excluded by the title |
| 1314 | Lopez-Patton, 2016 | Childhood trauma and METH abuse among men who have sex with men: Implications for intervention |  | Excluded by the title |
| 1315 | Messersmith, 2021 | Childhood Trauma, Gender Inequitable Attitudes, Alcohol Use and Multiple Sexual Partners: Correlates of Intimate Partner Violence in Northern Tanzania | 10.1177/0886260517731313 | Excluded by the title |
| 1316 | Chen, 2021 | Children as messengers of health knowledge? Impact of health promotion and water infrastructure in schools on facial cleanliness and trachoma in the community | 10.1371/journal.pntd.0009119 | Excluded by the title |
| 1317 | Saile, 2016 | Children of the postwar years: A two-generational multilevel risk assessment of child psychopathology in northern Uganda | 10.1017/s0954579415001066 | Excluded by the title |
| 1318 | Stobaugh, 2018 | Children with Poor Linear Growth Are at Risk for Repeated Relapse to Wasting after Recovery from Moderate Acute Malnutrition | 10.1093/jn/nxy033 | Excluded by the title |
| 1319 | Abate, 2019 | Chronic Malnutrition Among Under Five Children of Ethiopia May Not Be Economic. A Systematic Review and Meta-Analysis | 10.4314/ejhs.v29i2.14 | Excluded by the title |
| 1320 | Willcox, 2018 | Circumstances of child deaths in Mali and Uganda: a community-based confidential enquiry | 10.1016/s2214-109x(18)30215-8 | Excluded by the title |
| 1321 | Grimbeek, 2022 | Clinical and growth outcomes of severely malnourished children following hospital discharge in a South African setting | 10.1371/journal.pone.0262700 | Excluded by the title |
| 1322 | Atkilt, 2017 | Clinical Characteristics of Diabetic Ketoacidosis in Children with Newly Diagnosed Type 1 Diabetes in Addis Ababa, Ethiopia: A Cross-Sectional Study | 10.1371/journal.pone.0169666 | Excluded by the title |
| 1323 | Nasir, 2023 | Clinical Characteristics, Treatment Outcome and Associated Factors of Epilepsy Among Children at Hospitals of North-West Ethiopia | 10.2147/PHMT.S436022 | Excluded by the title |
| 1324 | Sutcliffe, 2016 | A clinical guidance tool to improve the care of children hospitalized with severe pneumonia in Lusaka, Zambia | 10.1186/s12887-016-0665-z | Excluded by the title |
| 1325 | Siril, 2017 | CLINICAL outcomes and loss to follow-up among people living with HIV participating in the NAMWEZA intervention in Dar es Salaam, Tanzania: a prospective cohort study |  | Excluded by the title |
| 1326 | Tekliye, 2021 | linical, immunologic and virologic outcomes of children and adolescents receiving second line anti-retroviral therapy in two referral hospitals in Addis Ababa, Ethiopia |  | Excluded by the title |
| 1327 | Diana, 2022 | Clinicopathological correlation of kidney disease in HIV infection pre- and post-ART rollout | 10.1371/journal.pone.0269260 | Excluded by the title |
| 1328 | Geus, 2019 | Co-infections with Plasmodium, Ascaris and Giardia among Rwandan schoolchildren | 10.1111/tmi.13206 | Excluded by the title |
| 1329 | Sahiledengle, 2023 | Coexistence of Anaemia and Stunting among Children Aged 6-59 Months in Ethiopia: Findings from the Nationally Representative Cross-Sectional Study | 10.3390/ijerph20136251 | Excluded by the title |
| 1330 | Buthamira, 2022 | Cognitive appraisal and adherece to antiretroviral therapy among elderly persons living with HIV: a case of Kakiri Health centre IV, HIV clinic |  | Excluded by the title |
| 1331 | Dakum, 2020 | Cohort profile: the Nigerian HIV geriatric cohort study |  | Excluded by the title |
| 1332 | Ogunbosi, 2020 | Colonisation with extended spectrum beta-lactamase-producing and carbapenem-resistant Enterobacterales in children admitted to a paediatric referral hospital in South Africa | 10.1371/journal.pone.0241776 | Excluded by the title |
| 1333 | Haile, 2019 | Colonization rate of Streptococcus pneumoniae, its associated factors and antimicrobial susceptibility pattern among children attending kindergarten school in Hawassa, southern Ethiopia | 10.1186/s13104-019-4376-z | Excluded by the title |
| 1334 | Usuzaki, 2021 | Commentary on "Determinants of pre-eclampsia among pregnant women attending perinatal care in hospitals of the Omo district, Southern Ethiopia" | 10.1111/jch.14110 | Excluded by the title |
| 1335 | Waiswa, 2016 | Community and District Empowerment for Scale-up (CODES): a complex district-level management intervention to improve child survival in Uganda: study protocol for a randomized controlled trial | 10.1186/s13063-016-1241-4 | Excluded by the title |
| 1336 | Ogunbosi, 2020 | Colonisation with extended spectrum beta-lactamase-producing and carbapenem-resistant Enterobacterales in children admitted to a paediatric referral hospital in South Africa | 10.1371/journal.pone.0241776 | Excluded by the title |
| 1337 | Dziva Chikwari, 2018 | Community health worker support to improve HIV treatment outcomes for older children and adolescents in Zimbabwe: A process evaluation of the ZENITH trial | 10.1186/s13012-018-0762-5 | Excluded by the title |
| 1338 | Caballero, 2021 | Community Mortality Due to Respiratory Syncytial Virus in Argentina: Population-based Surveillance Study | 10.1093/cid/ciab497 | Excluded by the title |
| 1339 | Oluoch, 2019 | Community perceptions affecting uptake & retention on antiretroviral therapy by PlHIV: A qualitative study among residents of an urban informal settlement in Kenya |  | Excluded by the title |
| 1340 | Medina-Marino, 2021 | The Community PrEP Study: a randomized control trial leveraging community-based platforms to improve access and adherence to pre-exposure prophylaxis to prevent HIV among adolescent girls and young women in South Africa—study protocol |  | Excluded by the title |
| 1341 | Chiao, 2017 | Community vulnerability and symptoms of acute respiratory infection among preschool age children in the Democratic Republic of Congo, Malawi and Nigeria: evidence from Demographic and Health Surveys | 10.1136/jech-2015-206605 | Excluded by the title |
| 1342 | le Roux, 2017 | Community-acquired pneumonia in children - a changing spectrum of disease | 10.1007/s00247-017-3827-8 | Excluded by the title |
| 1343 | Moramarco, 2016 | Community-Based Management of Child Malnutrition in Zambia: HIV/AIDS Infection and Other Risk Factors on Child Survival | 10.3390/ijerph13070666 | Excluded by the title |
| 1344 | Overbey, 2019 | Comparison of 1-week and 2-week recall periods for caregiver-reported diarrhoeal illness in children, using nationally representative household surveys | 10.1093/ije/dyz043 | Excluded by the title |
| 1345 | Kumela, 2015 | Comparison of anti-retroviral therapy treatment strategies in prevention of mother-to-child transmission in a teaching hospital in Ethiopia | 10.18549/pharmpract.2015.02.539 | Excluded by the title |
| 1346 | Mwalumuli, 2017 } | Comparison of level and predictors of adherence to art option b+ between HIV infected pregnant and lactating women at Mnazi Mmoja hospital Dar es salam Tanzania |  | Excluded by the title |
| 1347 | Scott, 2018 | Comparison of methods to measure ART adherence in children and young people living with HIV: Analysis of data from the BREATHER trial |  | Excluded by the title |
| 1348 | Woolley, 2021 | Comparison of Respiratory Health Impacts Associated with Wood and Charcoal Biomass Fuels: A Population-Based Analysis of 475,000 Children from 30 Low- and Middle-Income Countries | 10.3390/ijerph18179305 | Excluded by the title |
| 1349 | Johansen Ø, 2022 | A comparison of risk factors for cryptosporidiosis and non-cryptosporidiosis diarrhoea: A case-case-control study in Ethiopian children | 10.1371/journal.pntd.0010508 | Excluded by the title |
| 1350 | Denayer, 2021 | Comparison of risk stratification models for pregnancy in congenital heart disease | 10.1016/j.ijcard.2020.09.0 | Excluded by the title |
| 1351 | Hailu, 2021 | Complementary Feeding Practices and Nutritional Status of Infants Attending Hawassa University Comprehensive Specialized Hospital, Sidama Regional State, Ethiopia |  | Excluded by the title |
| 1352 | Natuhamya, 2023 | Complete sources of cluster variation on the risk of under-five malaria in Uganda: a multilevel-weighted mixed effects logistic regression model approach | DOI: 10.1186/s12936-023-04756-3 | Excluded by the title |
| 1353 | Chikako, 2021 | Complex Multilevel Modelling of the Individual, Household and Regional Level Variability in Predictors of Undernutrition among Children Aged 6-59 Months in Ethiopia | 10.3390/nu13093018 | Excluded by the title |
| 1354 | Abdulrahman, 2019 | Conceptual Framework for Investigating and Influencing Adherence Behavior among HIV-Positive Populations: An Applied Social Cognition Model |  | Excluded by the title |
| 1355 | Mohammed, 2019 | oncurrent anemia and stunting in young children: prevalence, dietary and non-dietary associated factors | 10.1186/s12937-019-0436-4 | Excluded by the title |
| 1356 | Zenebe, 2021 | Congenital Cytomegalovirus Infections Mother-Newborn Pair Study in Southern Ethiopia | 10.1155/2021/4646743 | Excluded by the title |
| 1357 | Adejumo, 2015 | Contemporary issues on the epidemiology and antiretroviral adherence of HIV-infected adolescents in sub-Saharan Africa: A narrative review | 10.7448/IAS.18.1.20049 | Excluded by the title |
| 1358 | Phiri, 2022 | Contextual factors and spatial trends of childhood malnutrition in Zambia | 10.1371/journal.pone.0277015 | Excluded by the title |
| 1359 | Glynn, 2023 | Contribution of remote M.tuberculosis infection to tuberculosis disease: A 30-year population study | 10.1371/journal.pone.0278136 | Excluded by the title |
| 1360 | Muluya, 2019 | Correlates of Non-adherence to Anti-Retroviral Therapy (ART) among Adolescents at Namungalwe Health Centre IV Iganga District |  | Excluded by the title |
| 1361 | Surti, 2021 | Correlation between Stigma and Adherence to ART among HIV-Positive Adolescents: A Cross-Sectional Study |  | Excluded by the title |
| 1362 | Young, 2020 | Correlation of pre-exposure prophylaxis adherence to a mental health diagnosis or experience of childhood trauma in high-risk youth |  | Excluded by the title |
| 1363 | Nigatu, 2019 | Cotrimoxazole prophylaxis treatment adherence and associated factors among human immunodeficiency virus (HIV) exposed children in public hospitals in ilubabor zone, Southwest Ethiopia, 2018 | 10.2174/1874944501912010184 | Excluded by the title |
| 1364 | Klingberg, 2018 | Courage and confidence to stop lying: caregiver perspectives on a video to support paediatric HIV disclosure in Kampala, Uganda | 10.2989/16085906.2018.1521850 | Excluded by the title |
| 1365 | Adetifa, 2018 | Coverage and timeliness of vaccination and the validity of routine estimates: Insights from a vaccine registry in Kenya | 10.1016/j.vaccine.2018.11.005 | Excluded by the title |
| 1366 | Verduci, 2019 | Cow's Milk Substitutes for Children: Nutritional Aspects of Milk from Different Mammalian Species, Special Formula and Plant-Based Beverages | 10.3390/nu11081739 | Excluded by the title |
| 1367 | Tadesse, 2015 | Cross sectional characterization of factors associated with pediatric HIV status disclosure in southern Ethiopia |  | Excluded by the title |
| 1368 | Luma, 2017 | Cross-sectional assessment of three commonly used measures of adherence to combination antiviral therapy in a resource limited setting |  | Excluded by the title |
| 1369 | Worrell, 2016 | A Cross-Sectional Study of Water, Sanitation, and Hygiene-Related Risk Factors for Soil-Transmitted Helminth Infection in Urban School- and Preschool-Aged Children in Kibera, Nairobi | 10.1371/journal.pone.0150744 | Excluded by the title |
| 1370 | Husen, 2022 | Cross-Sectional Study on Assessment of Frequency of Intestinal Helminth Infections and Its Related Risk Factors among School Children from Adola Town, Ethiopia | 10.1155/2022/5908938 | Excluded by the title |
| 1371 | Palmeirim, 2021 | A cross-sectional survey on parasitic infections in schoolchildren in a rural Tanzanian community | 10.1016/j.actatropica.2020.105737 | Excluded by the title |
| 1372 | Evangeli, 2023 | Cultural Adaption, Translation, Preliminary Reliability and Validity of Key Psychological and Behavioural Measures for 18 to 25 Year-Olds Living with HIV in Uganda: A Multi-Stage Approach |  | Excluded by the title |
| 1373 | Meisner, 2019 | The curse of dimensionality: Animal-related risk factors for pediatric diarrhea in western Kenya, and methods for dealing with a large number of predictors | 10.1371/journal.pone.0215982 | Excluded by the title |
| 1374 | Seni, 2019 | Deciphering risk factors for blood stream infections, bacteria species and antimicrobial resistance profiles among children under five years of age in North-Western Tanzania: a multicentre study in a cascade of referral health care system | 10.1186/s12887-019-1411-0 | Excluded by the title |
| 1375 | Smith, 2017 | Delayed Breastfeeding Initiation Is Associated with Infant Morbidity |  | Excluded by the title |
| 1376 | Govender, 2016 | Delayed diagnosis of anorectal malformations (ARM): causes and consequences in a resource-constrained environment | 10.1007/s00383-016-3866-5 | Excluded by the title |
| 1377 | Gelagay, 2015 | Demand for long acting contraceptive methods among married HIV positive women attending care at public health facilities at Bahir Dar City, Northwest Ethiopia | 10.1186/s12978-015-0073-0 | Excluded by the title |
| 1378 | Khalifa, 2019 | Demographic change and HIV epidemic projections to 2050 for adolescents and young people aged 15-24 | 10.1080/16549716.2019.1662685 | Excluded by the title |
| 1379 | Mohammadi Firouzeh, 2016 | Demographic, clinical and laboratory profiles of HIV infected patients admitted into Imam Khomeini Hospital of Tehran, Iran |  | Excluded by the title |
| 1380 | Mpimbaza, 2017 | Demographic, Socioeconomic, and Geographic Factors Leading to Severe Malaria and Delayed Care Seeking in Ugandan Children: A Case-Control Study | 10.4269/ajtmh.17-0056 | Excluded by the title |
| 1381 | Ha, 2019 | Depression among alcohol consuming, HIV positive men on ART treatment in India |  | Excluded by the title |
| 1382 | Zia, 2023 | Depression and PrEP uptake, interruption, and adherence among young women in Uganda |  | Excluded by the title |
| 1383 | Olashore, 2022 | Depression and suicidal behavior among adolescents living with HIV in Botswana: a cross-sectional study | 10.1186/s13034-022-00492-9 | Excluded by the title |
| 1384 | Reddy, 2021 | Depression, anxiety and treatment satisfaction in the parents of children on antiretroviral therapy in South Africa |  | Excluded by the title |
| 1385 | Bukenya, 2022 | Depression, anxiety, and suicide risk among Ugandan youth in vocational training |  | Excluded by the title |
| 1386 | Haro, 2023 | Dermatophytosis and its risk factors among children visiting dermatology clinic in Hawassa Sidama, Ethiopia | 10.1038/s41598-023-35837-7 | Excluded by the title |
| 1387 | Kisera, 2020 | A descriptive cross-sectional study of cholera at Kakuma and Kalobeyei refugee camps, Kenya in 2018 | 10.11604/pamj.2020.37.197.24798 | Excluded by the title |
| 1388 | Stofberg, 2020 | A descriptive study of suspected perinatal asphyxia at Mitchells Plain District Hospital: A case series | 10.4102/safp.v62i1.5112 | Excluded by the abstract |
| 1389 | Nyatabana, 2015 | Determinants affecting adherence to antiretroviral therapy in patients receiving free treatment at the wellness clinic of the Bela Bela District Hospital, Limpopo Province |  | Excluded by the title |
| 1390 | Rogawski, 2017 | Determinants and Impact of Giardia Infection in the First 2 Years of Life in the MAL-ED Birth Cohort | 10.1093/jpids/piw082 | Excluded by the title |
| 1391 | Oluwaseun, 2021 | Determinants contributing to adherence with antiretroviral regimen of people living with HIV/AIDS in Babcock University Teaching Hospital |  | Excluded by the title |
| 1392 | Adeniran, 2021 | Determinants of Adherence among Patients on Highly Active Anti-Retroviral Therapy in Lagos State, Nigeria |  | Excluded by the title |
| 1393 | Ondiek, 2018 | Determinants of adherence to anti-retroviral therapy among discordant couples in Usigu Division, Bondo sub-county, Kenya |  | Excluded by the title |
| 1394 | Heestermans, 2016 | Determinants of adherence to antiretroviral therapy among HIV-positive adults in sub-Saharan Africa: a systematic review |  | Excluded by the abstract |
| 1395 | Beletew, 2020 | Determinants of Anemia among HIV-Positive Children on Highly Active Antiretroviral Therapy Attending Hospitals of North Wollo Zone, Amhara Region, Ethiopia, 2019: A Case-Control Study | 10.1155/2020/3720572 | Excluded by the title |
| 1396 | Alamneh, 2023 | Determinants of anemia severity levels among children aged 6-59 months in Ethiopia: Multilevel Bayesian statistical approach |  | Excluded by the title |
| 1397 | Ahmadi, 2018 | Determinants of child anthropometric indicators in Ethiopia | 10.1186/s12889-018-5541- | Excluded by the title |
| 1398 | Ramlagan, 2018 | Determinants of disclosure and non-disclosure of HIV-positive status, by pregnant women in rural South Africa |  | Excluded by the title |
| 1399 | Njolomole, 2016 | Determinants of HIV related stigma and discrimination among healthcare professionals at a health facility in Malawi |  | Excluded by the title |
| 1400 | Anjajo, 2023 | Determinants of hypertension among diabetic patients in southern Ethiopia: a case-control study | 10.1186/s12872-023-03245- | Excluded by the title |
| 1401 | Bifftu, 2022 | Determinants of Intimate Partner Violence against Pregnant Women in Ethiopia: A Systematic Review and Meta-Analysis | 10.1155/2022/4641343 | Excluded by the title |
| 1402 | Wanjiku, 2022 | Determinants of Involvement in Antenatal Care among Male Police Officers at Anti-Stock Theft Unit in Gilgil Ward, Nakuru County; Kenya |  | Excluded by the title |
| 1403 | KEBAYA, 2023 | Determinants of linkage to HIV care and treatment among men who have sex with men (msm) in Kisumu county, Kenya |  | Excluded by the title |
| 1404 | Semakula, 2023 | Determinants of malaria infections among children in refugee settlements in Uganda during 2018-2019 | 10.1186/s40249-023-01090-3 | Excluded by the title |
| 1405 | KEBAYA, 2023 | Determinants of linkage to HIV care and treatment among men who have sex with men (msm) in Kisumu county, Kenya |  | Excluded by the title |
| 1406 | Amare, 2019 | Determinants of non-compliance with antiretroviral therapy in adult patients in Kinshasa | 10.1186/s40249-023-01090-3 | Excluded by the title |
| 1407 | MULI, 2023 | Determinants of mother to child transmission of HIV among exposed infants in Kericho County Referral Hospital, Kenya |  | Excluded by the title |
| 1408 | Izizag, 2020 | Determinants of non-compliance with antiretroviral therapy in adult patients in Kinshasa | 10.1186/s40249-023-01090-3 | Excluded by the title |
| 1409 | Amare, 2019 | Determinants of nutritional status among children under age 5 in Ethiopia: further analysis of the 2016 Ethiopia demographic and health survey | 10.1186/s12992-019-0505-7 | Excluded by the title |
| 1410 | Dhlakama, 2023 | Determinants of option B+ treatment adherence among HIV-positive breastfeeding women in Zimbabwe | 10.29063/ajrh2023/v27i12.2 | Excluded by the title |
| 1411 | Seramo, 2022 | Determinants of pneumonia among children attending public health facilities in Worabe town | 10.1038/s41598-022-10194-z | Excluded by the title |
| 1412 | Fikadu, 2021 | Determinants of pre-eclampsia among pregnant women attending perinatal care in hospitals of the Omo district, Southern Ethiopia | 10.1111/jch.14073 | Excluded by the title |
| 1413 | Asfaw, 2020 | Determinants of soil-transmitted helminth infections among pre-school-aged children in Gamo Gofa zone, Southern Ethiopia: A case-control study | 10.1371/journal.pone.0243836 | Excluded by the title |
| 1414 | Rakotomanana, 2017 | Determinants of stunting in children under 5 years in Madagascar | 10.1111/mcn.12409 | Excluded by the title |
| 1415 | Woodruff, 2017 | Determinants of stunting reduction in Ethiopia 2000 – 2011 | 10.1111/mcn.12307 | Excluded by the title |
| 1416 | Chadambuka, 2019 | Determinants of Treatment Adherence and Retention in Care among HIV Positive Pregnant and Breastfeeding Women in a Rural District in Zimbabwe |  | Excluded by the title |
| 1417 | Ogbo, 2019 | Determinants of trends in neonatal, post-neonatal, infant, child and under-five mortalities in Tanzania from 2004 to 2016 | 10.1186/s12889-019-7547-x | Excluded by the title |
| 1418 | Kassie, 2020 | Determinants of under-nutrition among children under five years of age in Ethiopia | 10.1186/s12889-020-08539-2 | Excluded by the title |
| 1419 | Ally, 2023 | Determinants of viral load suppression among orphaned and vulnerable children living with HIV on ART in Tanzania | 10.3389/fpubh.2023.1076614 | Excluded by the title |
| 1420 | Elashi, 2021 | Determinants of viral suppression among adolescents on antiretroviral therapy in Thabo Mofutsanyane District Municipality, Free State province, South Africa |  | Excluded by the title |
| 1421 | Shumetie, 2021 | Determinants of Virological Failure AmongHIV-Infected Children on First-Line Antiretroviral Therapy in West Gojjam Zone, Amhara Region, Ethiopia | 10.2147/HIV.S334067 | Excluded by the title |
| 1422 | Dawa, 2019 | Developing a seasonal influenza vaccine recommendation in Kenya: Process and challenges faced by the National Immunization Technical Advisory Group (NITAG) | 10.1016/j.vaccine.2018.11.062 | Excluded by the title |
| 1423 | Karugaba, 2020 | Developing policy guidelines to promote quality of life of young adults with perinatally acquired HIV in Botswana |  | Excluded by the title |
| 1424 | Swendeman, 2015 | Development and pilot testing of daily interactive voice response (IVR) calls to support antiretroviral adherence in India: a mixed-methods pilot study |  | Excluded by the title |
| 1425 | Ashaba, 2019 | Development and validation of a 20-item screening scale to detect major depressive disorder among adolescents with HIV in rural Uganda: A mixed-methods study |  | Excluded by the title |
| 1426 | Mutumba, 2015 | Development of a psychosocial distress measure for Ugandan adolescents living with HIV |  | Excluded by the title |
| 1427 | Wright, 2021 | Development of a Scale to Measure Infant Eating Behaviour Worldwide | 10.3390/nu13082495 | Excluded by the title |
| 1428 | Hasegawa, 2017 | Development of a screening tool to predict malnutrition among children under two years old in Zambia | 10.1080/16549716.2017.1339981 | Excluded by the title |
| 1429 | Kaimila, 2019 | Development of Acute Malnutrition Despite Nutritional Supplementation in Malawi | 10.1097/mpg.0000000000002241 | Excluded by the title |
| 1430 | Muri, 2017 | Development of HIV drug resistance and therapeutic failure in children and adolescents in rural Tanzania: An emerging public health concern | 10.1097/QAD.0000000000001273 | Excluded by the title |
| 1431 | Davies, 2018 | Developmental origins of health and disease in Africa-influencing early life | 10.1016/s2214-109x(18)30036-6 | Excluded by the title |
| 1432 | Abessa, 2017 | Developmental performance of hospitalized severely acutely malnourished under-six children in low- income setting | 10.1186/s12887-017-0950-5 | Excluded by the title |
| 1433 | Chiwungwe, 2017 | Diabetes-related knowledge, attitudes and practices [KAP] of adult patients with type 2 diabetes in Maseru, Lesotho |  | Excluded by the title |
| 1434 | Adom, 2019 | Diagnostic Accuracy of Body Mass Index in Defining Childhood Obesity: Analysis of Cross-Sectional Data from Ghanaian Children |  | Excluded by the title |
| 1435 | Mbuthia, 2019 | Diarrheal correlates associated with enteric bacterial infections among children below five years in Murang'a County, Kenya | 10.11604/pamj.2019.34.170.17403 | Excluded by the title |
| 1436 | Nhampossa, 2015 | Diarrheal Disease in Rural Mozambique: Burden, Risk Factors and Etiology of Diarrheal Disease among Children Aged 0-59 Months Seeking Care at Health Facilities | 10.1371/journal.pone.0119824 | Excluded by the title |
| 1437 | Gebrezgiabher, 2019 | Diarrheal disease in under-five children among model and non-model families in northern Ethiopia, 2017: a comparative cross-sectional study | 10.1186/s13104-019-4322-0 | Excluded by the title |
| 1438 | Nguyen, 2021 | Diarrhoea among Children Aged under Five Years and Risk Factors in Informal Settlements: A Cross-Sectional Study in Cape Town, South Africa | 10.3390/ijerph18116043 | Excluded by the title |
| 1439 | Mohamed, 2018 | Diet and hygiene practices influence morbidity in schoolchildren living in Schistosomiasis endemic areas along Lake Victoria in Kenya and Tanzania-A cross-sectional study | 10.1371/journal.pntd.0006373 | Excluded by the title |
| 1440 | Shallo, 2020 | Hiv positive status disclosure and its associated factors among children on antiretroviral therapy in west shoa zone, western ethiopia, 2019: A mixed method cross-sectional study | 10.2147/JMDH.S258851 | Excluded by the title |
| 1441 | Ndlovu, 2019 | Differentiated service delivery for adolescents and young people living with HIV: a situational analysis in South Africa |  | Excluded by the title |
| 1442 | Kwagala, 2022 | Disability status, partner behavior, and the risk of sexual intimate partner violence in Uganda: An analysis of the demographic and health survey data | 10.1186/s12889-022-14273-8 | Excluded by the title |
| 1443 | Amankwah-Poku, 2021 | Disclosure and health-related outcomes among children living with HIV and their caregivers | 10.1186/s12981-021-00337-z | Excluded by the title |
| 1444 | Brittain, 2019 | Disclosure of HIV status among HIV-infected pregnant and postpartum women in Cape Town, South Africa |  | Excluded by the title |
| 1445 | Akilimali, 2017 | Disclosure of HIV status and its impact on the loss in the follow-up of HIV-infected patients on potent anti-retroviral therapy programs in a (post-) conflict setting: A retrospective cohort study from Goma, Democratic Republic of Congo |  | Excluded by the title |
| 1446 | Kashala Abotnes, 2017 | Disclosure of HIV status and its impact on the loss in the follow-up of HIV-infected patients on potent anti-retroviral therapy programs ina (post-) conflict setting: A retrospective cohort study from Goma, Democratic Republic of Congo |  | Excluded by the title |
| 1447 | Nwoyeocha, 2020 | Disclosure of paediatric HIV status to infected children and significant others: Impact and psychosocial outcomes |  | Excluded by the title |
| 1448 | Weldekidan, 2022 | Discontinuation rate of long-acting reversible contraceptives and associated factors among reproductive-age women in Butajira town, Central Ethiopia | 10.1177/17455057221104656 | Excluded by the title |
| 1449 | Yap, 2015 | Disease, activity and schoolchildren's health (DASH) in Port Elizabeth, South Africa: a study protocol | 10.1186/s12889-015-2636-y | Excluded by the title |
| 1450 | Asmare, 2022 | Disparities in full immunization coverage among urban and rural children aged 12-23 months in southwest Ethiopia: A comparative cross-sectional study | 10.1080/21645515.2022.2101316 | Excluded by the title |
| 1451 | Ferreira, 2023 | Disparities in HIV continuum of care in the paediatric population: A real-life study in Brazil |  | Excluded by the title |
| 1452 | Salim, 2015 | Distribution and risk factors for Plasmodium and helminth co-infections: a cross-sectional survey among children in Bagamoyo district, coastal region of Tanzania | 10.1371/journal.pntd.0003660 | Excluded by the title |
| 1453 | Wampande, 2015 | Distribution and transmission of Mycobacterium tuberculosis complex lineages among children in peri-urban Kampala, Uganda | 10.1186/s12887-015-0455-z | Excluded by the title |
| 1454 | Mohr, 2017 | DOT or SAT for Rifampicin-resistant tuberculosis? A non-randomized comparison in a high HIV-prevalence setting |  | Excluded by the title |
| 1455 | Howland, 2015 | Drinking, Despair and the State: An Ethnography of a Brewing Subculture in Rural Kenya |  | Excluded by the title |
| 1456 | Deichsel, 2023 | Drivers of Decline in Diarrhea Mortality Between GEMS and VIDA Studies | 10.1093/cid/ciad015 | Excluded by the title |
| 1457 | Argaw, 2019 | Drivers of Under-Five Stunting Trend in 14 Low- and Middle-Income Countries since the Turn of the Millennium: A Multilevel Pooled Analysis of 50 Demographic and Health Surveys | 10.3390/nu11102485 | Excluded by the title |
| 1458 | Bedecha, 2023 | Dual contraception method utilization and associated factors among women on antiretroviral therapy in public facilities of Bishoftu town, Oromia, Ethiopia | 10.1371/journal.pone.0280447 | Excluded by the title |
| 1459 | Jemberie, 2023 | Dual Contraceptive Method Utilization and Associated Factors Among HIV Positive Women Attending ART Clinic in Finote-Selam Hospital: Cross-Sectional Study | 10.1007/s10508-023-02593-8 | Excluded by the title |
| 1460 | Shilaih, 2016 | Dually active HIV/HBV antiretrovirals as protection against incident hepatitis B infections: potential for prophylaxis |  | Excluded by the title |
| 1461 | Steyn, 2019 | Duane's Retraction Syndrome in a Cohort of South African Children: A 20-Year Clinic-Based Review | 10.3928/01913913-20190416-01 | Excluded by the title |
| 1462 | Iyun, 2020 | Earlier Antiretroviral Therapy Initiation and Decreasing Mortality Among HIV-infected Infants Initiating Antiretroviral Therapy Within 3 Months of Age in South Africa, 2006–2017 | 10.1097/INF.0000000000002516 | Excluded by the title |
| 1463 | Scharf, 2018 | Early childhood growth and cognitive outcomes: Findings from the MAL-ED study | 10.1111/mcn.12584 | Excluded by the title |
| 1464 | Samuri, 2018 | Early Childhood Research Landscape on Children’s Profile: Coherent Taxonomy, Motivation, Open Challenges, Recommendations and, Pathways for Future Research |  | Excluded by the title |
| 1465 | Ojwang, 2020 | Early exposure to cats, dogs and farm animals and the risk of childhood asthma and allergy | 10.1111/pai.13186 | Excluded by the title |
| 1466 | Kohli-Lynch, 2019 | Early Intervention for Children at High Risk of Developmental Disability in Low- and Middle-Income Countries: A Narrative Review | 10.3390/ijerph16224449 | Excluded by the title |
| 1467 | Munthali, 2017 | Early Life Growth Predictors of Childhood Adiposity Trajectories and Future Risk for Obesity: Birth to Twenty Cohort | 10.1089/chi.2016.0310 | Excluded by the title |
| 1468 | Sania, 2019 | Early life risk factors of motor, cognitive and language development: a pooled analysis of studies from low/middle-income countries | 10.1136/bmjopen-2018-026449 | Excluded by the title |
| 1469 | Temesgen, 2021 | Early Outcome of Laparotomy Wounds in Pediatric Patients in TASH, Addis Ababa, Ethiopia: A Six-Months Prospective Study | 10.4314/esssssjhs.v31i1.13 | Excluded by the title |
| 1470 | Kalberg, 2019 | Early-Life Predictors of Fetal Alcohol Spectrum Disorders | 10.1542/peds.2018-2141 | Excluded by the title |
| 1471 | Ngwenya, 2022 | Ecological risk modelling of antiretroviral drugs in the aquatic environment |  | Excluded by the title |
| 1472 | Blackburn, 2020 | An Economic Evaluation of an Alcohol Reduction Intervention in the HIV Clinic Setting in Vietnam |  | Excluded by the title |
| 1473 | Lukyamuzi, 2021 | Effect and implementation experience of intensive adherence counseling in a public HIV care center in Uganda: a mixed-methods study |  | Excluded by the abstract |
| 1474 | Pramila, 2017 | Effect of adherence to anti-retroviral therapy on cd4 t cells and hiv viral load in Nepalese Tertiary Care Hospital |  | Excluded by the title |
| 1475 | Davies, 2022 | Effect of antiretroviral therapy care interruptions on mortality in children living with HIV | 10.1097/QAD.0000000000003194 | Excluded by the title |
| 1476 | Mbita, 2019 | Effect of antiretroviral therapy on fertility rate among women living with HIV in Tabora, Tanzania: An historical cohort study | 10.1371/journal.pone.0222173 | Excluded by the title |
| 1477 | Velloza, 2018 | Effect of depression on adherence to oral PrEP among men and women in East Africa |  | Excluded by the title |
| 1478 | Mugo, 2023 | Effect of Dolutegravir and Multimonth Dispensing on Viral Suppression among Children with HIV | 10.1097/QAI.0000000000003190 | Excluded by the title |
| 1479 | Ezenwosu, 2023 | Effect of dolutegravir-based drug combinations on the level of medication adherence and viral load among adolescents living with HIV in resource-limited setting: a pre-post design |  | Excluded by the abstract |
| 1480 | Mutasa‐Apollo, 2017 | Effect of frequency of clinic visits and medication pick‐up on antiretroviral treatment outcomes: a systematic literature review and meta‐analysis |  | Excluded by the title |
| 1481 | Nakimuli-Mpungu, 2023 | The effect of group support psychotherapy on adherence to anti-retroviral therapy and viral suppression among HIV positive young people: Study protocol for a pilot randomized controlled trial |  | Excluded by the title |
| 1482 | Tjituka, 2018 | The effect of HIV status disclosure and non-disclosure on the psychosocial wellbeing, adherence levels and treatment outcomes of adolescents on Highly Active Anti-Retroviral Therapy (HAART) at Katututra State Hospital, Namibia |  | Excluded by the title |
| 1483 | YILMAZER, 2020 } | Effect of Information-Motivation-Behavioral Skills Model-Based Intervention on Quality of Life of Ostomy Patients |  | Excluded by the title |
| 1484 | Ambarwati, 2021 | The effect of iron deficiency anemia on the child death related to HIV/AIDS infection: meta-analysis |  | Excluded by the title |
| 1485 | Starck, 2022 | The effect of malaria on childhood anemia in a quasi-experimental study of 7,384 twins from 23 Sub-Saharan African countries | 10.3389/fpubh.2022.1009865 | Excluded by the title |
| 1486 | Starck, 2021 | The effect of malaria on haemoglobin concentrations: a nationally representative household fixed-effects study of 17,599 children under 5 years of age in Burkina Faso | 10.1186/s12936-021-03948-z | Excluded by the title |
| 1487 | Blessy, 2016 | Effect of micro and macro nutritient supplementation on disease outcome in adolescents with HIV on HAART: A Randomised Double-blinded clinical trial |  | Excluded by the title |
| 1488 | Grudziak, 2017 | The effect of pre-existing malnutrition on pediatric burn mortality in a sub-Saharan African burn unit | 10.1016/j.burns.2017.03.022 | Excluded by the title |
| 1489 | Akindele, 2015 | The effect of socio-economic status on adherence to anti-retroviral therapy |  | Excluded by the title |
| 1490 | Bitew, 2018 | The effect of SODIS water treatment intervention at the household level in reducing diarrheal incidence among children under 5 years of age: a cluster randomized controlled trial in Dabat district, northwest Ethiopia | 10.1186/s13063-018-2797-y | Excluded by the title |
| 1491 | {Kaswa, 2023 | The effect of substance uses on antiretroviral therapy adherence among people living with HIV in Mthatha, Eastern Cape |  | Excluded by the title |
| 1492 | Tadesse, 2017 | Effect of water, sanitation and hygiene interventions on active trachoma in North and South Wollo zones of Amhara Region, Ethiopia: A Quasi-experimental study | 10.1371/journal.pntd.0006080 | Excluded by the title |
| 1493 | Magnolini, 2024 | Effectiveness and acceptance of group therapy as a mental health intervention for people living with HIV in Africa–a scoping literature review |  | Excluded by the title |
| 1494 | Mageda, 2023 | Effectiveness of a community-based intervention (Konga model) to address factors contributing to low viral load suppression among children living with HIV in Tanzania: a preliminary, cluster, randomized clinical trial report | 10.1186/s12889-023-16181-x | Excluded by the title |
| 1495 | Chinoda, 2020 | Effectiveness of a peer-led adolescent mental health intervention on HIV virological suppression and mental health in Zimbabwe: protocol of a cluster-randomised trial |  | Excluded by the title |
| 1496 | Soboka, 2015 | The effectiveness of counseling, material support and/or nutritional supplementation on improving adherence to anti-retroviral therapy and clinical outcomes among HIV patients: a systematic review of quantitative evidence protocol |  | Excluded by the title |
| 1497 | Sakthivel, 2021 | Effectiveness of HIV Intervention Package (HIP) on HIV Infected Adolescents: Pilot Study Report |  | Excluded by the title |
| 1498 | Prisma, 2023 | The Effectiveness of Mobile Phone Text Messages on the Adherence of Antiretroviral Drug Taking in Patients Living with HIV/AIDS: A Meta-Analysis |  | Excluded by the title |
| 1499 | bin Abdul Wahab, 2021 | Effectiveness of Phone Reminders to Improve Adherence to Anti-Retroviral Therapy: A Meta-Analysis |  | Excluded by the title |
| 1500 | Ribeiro, 2015 | Effectiveness of psycho-educational intervention in HIV patients’ treatment |  | Excluded by the title |
| 1501 | Cairns, 2021 | Effectiveness of seasonal malaria chemoprevention (SMC) treatments when SMC is implemented at scale: Case-control studies in 5 countries | 10.1371/journal.pmed.1003727 | Excluded by the title |
| 1502 | Odetola, 2016 | Effects of a nursing intervention using a mobile phone application on Uptake of antenatal care, tetanus toxoids and malaria prevention among pregnant women in Nigeria |  | Excluded by the title |
| 1503 | Muralidharan, 2015 | Effects of ART on CD4 Count and Body Weight in HIV/AIDS Patients using Longitudinal Analysis in the Case of Debre Berhan Referral Hospital |  | Excluded by the title |
| 1504 | Crea, 2015 | Effects of cash transfers on Children's health and social protection in Sub-Saharan Africa: differences in outcomes based on orphan status and household assets | 10.1186/s12889-015-1857-4 | Excluded by the title |
| 1505 | Colston, 2019 | Effects of Child and Maternal Histo-Blood Group Antigen Status on Symptomatic and Asymptomatic Enteric Infections in Early Childhood | 10.1093/infdis/jiz072 | Excluded by the title |
| 1506 | Sabourin, 2023 | Effects of Maternal HIV Infection on Early Kaposi Sarcoma-Associated Herpesvirus Seroconversion in a Ken an Mother-Infant Cohort | 10.1093/infdis/jiad310 | Excluded by the title |
| 1507 | Odetola, 2016 | Effects of mHealth Nursing Intervention on Uptake of Antenatal Care and Pregnancy Drugs Among Pregnant Women Attendees of PHC in Oyo State |  | Excluded by the title |
| 1508 | Strother, 2022 | Effects of psychosocial factors on nonadherence to ART in Ganta, Nimba county, Liberia |  | Excluded by the title |
| 1509 | Ugburo, 2015 | Effects of telephonic SMS reminders influence on adherence to scheduled medication pick up appointments among adults on antiretrovirals at the Swakopmund State Hospital ART clinic Namibia |  | Excluded by the title |
| 1510 | Namara, 2017 | Effects of treating helminths during pregnancy and early childhood on risk of allergy-related outcomes: Follow-up of a randomized controlled trial | 10.1111/pai.12804 | Excluded by the title |
| 1511 | Nguyen, 2022 | Effects of Two Alcohol Reduction Interventions on Depression and Anxiety Symptoms of ART Clients in Vietnam | 10.1007/s10461-021-03532-1 | Excluded by the title |
| 1512 | Bawn, 2022 | eHealth for family planning in Botswana: acceptability and feasibility |  | Excluded by the title |
| 1513 | Nkwana, 2019 | Emergent Literacy Support for Children from Marginalised Populations | 10.1159/000493893 | Excluded by the title |
| 1514 | Vinayagamoorthy, 2023 | Emergomycosis, an Emerging Thermally Dimorphic Fungal Infection: A Systematic Review |  | Excluded by the title |
| 1515 | Kefale, 2019 | Emotional and behavioral problems and associated factors among children and adolescents on highly active anti-retroviral therapy in public hospitals of West Gojjam zone, Amhara regional state of Ethiopia, 2018: A cross-sectional study | 10.1186/s12887-019-1453-3 | Excluded by the title |
| 1516 | Ouma, 2020 | Endothelial Activation, Acute Kidney Injury, and Cognitive Impairment in Pediatric Severe Malaria | 10.1097/ccm.0000000000004469 | Excluded by the title |
| 1517 | Nkenfou, 2021 | Enhanced passive surveillance dengue infection among febrile children: Prevalence, co-infections and associated factors in Cameroon | 10.1371/journal.pntd.0009316 | Excluded by the title |
| 1518 | Tsai, 2019 | Enteric Pathogen Diversity in Infant Foods in Low-Income Neighborhoods of Kisumu, Kenya | 10.3390/ijerph16030506 | Excluded by the title |
| 1519 | Feleke, 2018 | Enteric pathogens and associated risk factors among under-five children with and without diarrhea in Wegera District, Northwestern Ethiopia | 10.11604/pamj.2018.29.72.13973 | Excluded by the title |
| 1520 | Khabo-Mmekoa, 2022 | Enteric Pathogens Risk Factors Associated with Household Drinking Water: A Case Study in Ugu District Kwa-Zulu Natal Province, South Africa | 10.3390/ijerph19084431 | Excluded by the title |
| 1521 | Zelelie, 2019 | Enteropathogens in Under-Five Children with Diarrhea in Health Facilities of Debre Berhan Town, North Shoa, Ethiopia | 10.4314/ejhs.v29i2.7 | Excluded by the title |
| 1522 | Kinyoki, 2016 | Environmental predictors of stunting among children under-five in Somalia: cross-sectional studies from 2007 to 2010 | 10.1186/s12889-016-3320-6 | Excluded by the title |
| 1523 | Yeruva, 2022 | Enzyme Responsive Delivery of Anti-Retroviral Peptide via Smart Hydrogel |  | Excluded by the title |
| 1524 | Tsegaye, 2023 | Epidemiological survival pattern, risk factors, and estimated time to develop tuberculosis after test and treat strategies declared for children living with human immune deficiency virus | 10.1016/j.ijtb.2023.05.008  v | Excluded by the title |
| 1525 | Bauleth, 2020 | Epidemiology and factors associated with diarrhoea among children under five years of age in the Engela District in the Ohangwena Region, Namibia | 10.4102/phcfm.v12i1.2361 | Excluded by the title |
| 1526 | Korpe, 2018 | Epidemiology and Risk Factors for Cryptosporidiosis in Children From 8 Low-income Sites: Results From the MAL-ED Study | 10.1093/cid/ciy355 | Excluded by the title |
| 1527 | Bigna, 2018 | Epidemiology of depressive disorders in people living with HIV in Africa: a systematic review and meta-analysis |  | Excluded by the title |
| 1528 | Bigna, 2019 | Epidemiology of depressive disorders in people living with HIV in Africa: a systematic review and meta-analysis: Burden of depression in HIV in Africa |  | Excluded by the title |
| 1529 | Bwogi, 2016 | The epidemiology of rotavirus disease in under-five-year-old children hospitalized with acute diarrhea in central Uganda, 2012-2013 | 10.1007/s00705-015-2742-2 | Excluded by the title |
| 1530 | Rouhani, 2022 | The Epidemiology of Sapovirus in the Etiology, Risk Factors, and Interactions of Enteric Infection and Malnutrition and the Consequences for Child Health and Development Study: Evidence of Protection Following Natural Infection | 10.1093/cid/ciac165 | Excluded by the title |
| 1531 | Tefera, 2020 | Epidemiology of Schistosoma mansoni infection and associated risk factors among school children attending primary schools nearby rivers in Jimma town, an urban setting, Southwest Ethiopia | 10.1371/journal.pone.0228007 | Excluded by the title |
| 1532 | Kazembe, 2015 | Estimating areas of common risk in low birth weight and infant mortality in Namibia: a joint spatial analysis at sub-regional level | 10.1016/j.sste.2015.02.001 | Excluded by the title |
| 1533 | Rasheed, 2021 | Estimating the health burden of aflatoxin attributable stunting among children in low income countries of Africa | 10.1038/s41598-020-80356-4 | Excluded by the title |
| 1534 | Kimanya, 2021 | Estimating the risk of aflatoxin-induced liver cancer in Tanzania based on biomarker data | 10.1371/journal.pone.0247281 | Excluded by the title |
| 1535 | Mayer, 2020 | Estimating the Risk of Human Herpesvirus 6 and Cytomegalovirus Transmission to Ugandan Infants from Viral Shedding in Saliva by Household Contacts | 10.3390/v12020171 | Excluded by the title |
| 1536 | Shrestha, 2021 | HIV AIDS related knowledge among antiretroviral therapy clients at Kathmandu and Dhulikhel Nepal a cross-sectional study |  | Excluded by the title |
| 1537 | {Bongomin, 2021 | Estimation of the burden of tinea capitis among children in Africa | 10.1111/myc.13221 | Excluded by the title |
| 1538 | Gebreegziabher, 2019 | Ethiopia's high childhood undernutrition explained: analysis of the prevalence and key correlates based on recent nationally representative data | 10.1017/s1368980019000569 | Excluded by the title |
| 1539 | Shaikh, 2020 | Ethnic disparity and exposure to supplements rather than adverse childhood experiences linked to preterm birth in Pakistani women | 10.1016/j.jad.2020.01.180 | Excluded by the title |
| 1540 | Randremanana, 2016 | Etiologies, Risk Factors and Impact of Severe Diarrhea in the Under-Fives in Moramanga and Antananarivo, Madagascar | 10.1371/journal.pone.0158862 | Excluded by the abstract |
| 1541 | Howie, 2021 | The Etiology of Childhood Pneumonia in The Gambia: Findings From the Pneumonia Etiology Research for Child Health (PERCH) Study | 10.1097/inf.0000000000002766 | Excluded by the title |
| 1542 | Ebruke, 2021 | The Etiology of Pneumonia From Analysis of Lung Aspirate and Pleural Fluid Samples: Findings From the Pneumonia Etiology Research for Child Health (PERCH) Study | 10.1093/cid/ciaa1032 | Excluded by the title |
| 1543 | Moore, 2021 | The Etiology of Pneumonia in HIV-1-infected South African Children in the Era of Antiretroviral Treatment: Findings From the Pneumonia Etiology Research for Child Health (PERCH) Study | 10.1097/inf.0000000000002651 | Excluded by the title |
| 1544 | Seidenberg, 2021 | The Etiology of Pneumonia in HIV-infected Zambian Children: Findings From the Pneumonia Etiology Research for Child Health (PERCH) Study | 10.1097/inf.0000000000002649 | Excluded by the title |
| 1545 | Awori, 2021 | The Etiology of Pneumonia in HIV-uninfected Children in Kilifi, Kenya: Findings From the Pneumonia Etiology Research for Child Health (PERCH) Study | 10.1097/inf.0000000000002653 | Excluded by the title |
| 1546 | Moore, 2021 | The Etiology of Pneumonia in HIV-uninfected South African Children: Findings From the Pneumonia Etiology Research for Child Health (PERCH) Study | 10.1097/inf.0000000000002650 | Excluded by the abstract |
| 1547 | Mwananyanda, 2021 | The Etiology of Pneumonia in Zambian Children: Findings From the Pneumonia Etiology Research for Child Health (PERCH) Study | 10.1097/inf.0000000000002652 | Excluded by the title |
| 1548 | Buchwald, 2023 | Etiology, Presentation, and Risk Factors for Diarrheal Syndromes in 3 Sub-Saharan African Countries After the Introduction of Rotavirus Vaccines From the Vaccine Impact on Diarrhea in Africa (VIDA) Study | 10.1093/cid/ciad022 | Excluded by the title |
| 1549 | Obeagu, 2023 | EURASIAN EXPERIMENT JOURNAL OF MEDICINE AND MEDICAL SCIENCES (EEJMMS) ISSN: 2992-4103© EEJMMS Publications Volume 4 Issue 1 2023 |  | Excluded by the title |
| 1550 | Lule, 2023 | EURASIAN EXPERIMENT JOURNAL OF SCIENTIFIC AND APPLIED RESEARCH (EEJSAR) ISSN: 2992-4146© EEJSAR Publications Volume 4 Issue 1 2023 Factors associated with Adherence to Antiretroviral Treatment among Adolescents Attending Kalisizo |  | Excluded by the title |
| 1551 | Mitchell, 2022 | Evaluating malaria prevalence and land cover across varying transmission intensity in Tanzania using a cross-sectional survey of school-aged children | 10.1186/s12936-022-04107-8 | Excluded by the title |
| 1552 | Tadesse, 2023 | Evaluating self-medication practices in Ethiopia |  | Excluded by the title |
| 1553 | Ugwu, 2020 | Evaluating the Effects of Climate and Environmental Factors on Under-5 Children Malaria Spatial Distribution Using Generalized Additive Models (GAMs) | 10.2991/jegh.k.200814.001 | Excluded by the title |
| 1554 | Kelly, 2018 | Evaluation of a community-based ART programme after tapering home visits in rural Sierra Leone: a 24-month retrospective study |  | Excluded by the title |
| 1555 | Ka'e, 2023 | Evaluation of archived drug resistance mutations in HIV-1 DNA among vertically infected adolescents under antiretroviral treatment in Cameroon: Findings during the COVID-19 pandemic | 10.1111/hiv.13459 | Excluded by the title |
| 1556 | Grijalva-Eternod, 2023 | Evaluation of conditional cash transfers and mHealth audio messaging in reduction of risk factors for childhood malnutrition in internally displaced persons camps in Somalia: A 2 × 2 factorial cluster-randomised controlled trial | 10.1371/journal.pmed.1004180 | Excluded by the title |
| 1557 | Baye, 2020 | Evaluation of Linear Growth at Higher Altitudes |  | Excluded by the title |
| 1558 | Kadima, 2018 | Evaluation of non-adherence to anti-retroviral therapy, the associated factors and infant outcomes among HIV-positive pregnant women: a prospective cohort study in Lesotho |  | Excluded by the title |
| 1559 | Cronjé, 2022 | An Evaluation of Severe Anesthetic-Related Critical Incidents and Risks From the South African Paediatric Surgical Outcomes Study: A 14-Day Prospective, Observational Cohort Study of Pediatric Surgical Patients | 10.1213/ane.0000000000005796 | Excluded by the title |
| 1560 | Lippman, 2016 | Evaluation of short message service and peer navigation to improve engagement in HIV care in South Africa: study protocol for a three-arm cluster randomized controlled trial |  | Excluded by the title |
| 1561 | Babb, 2018 | Evaluation of the effectiveness of a latrine intervention in the reduction of childhood diarrhoeal health in Nyando District, Kisumu County, Kenya | 10.1017/s0950268818000924 | Excluded by the title |
| 1562 | Kajura, 2023 | Evaluation of the occurrence and factors responsible for Hypertension in HIV Patients on HAART attending Chai Clinic at Kampala International University Teaching Hospital |  | Excluded by the title |
| 1563 | Haeri Mazanderani, 2019 | Evolving complexities of infant HIV diagnosis within Prevention of Mother-to-Child Transmission programs | 10.12688/f1000research.19637.1 | Excluded by the title |
| 1564 | Benning, 2020 | Examining adherence barriers among women with HIV to tailor outreach for long-acting injectable antiretroviral therapy |  | Excluded by the title |
| 1565 | Bauhofer, 2023 | Examining comorbidities in children with diarrhea across four provinces of Mozambique: A cross-sectional study (2015 to 2019) | 10.1371/journal.pone.0292093 | Excluded by the title |
| 1566 | Bruser, 2020 | Examining Geographic and Social Barriers to HIV Treatment Adherence in Kampala, Uganda |  | Excluded by the title |
| 1567 | Amegbor, 2020 | Examining the Effect of Geographic Region of Residence on Childhood Malnutrition in Uganda | 10.1093/tropej/fmaa019 | Excluded by the title |
| 1568 | Mutumba, 2016 | Examining the relationship between psychological distress and adherence to anti-retroviral therapy among Ugandan adolescents living with HIV |  | Excluded by the title |
| 1569 | Nwankwo-Igomu, 2016 | Examining the Relationship of Family Support with Pediatric Adherence to HIV Antiretroviral Treatment in PEPFAR Care and Treatment Programs in Nigeria |  | Excluded by the title |
| 1570 | Damulira, 2019 | Examining the relationship of social support and family cohesion on ART adherence among HIV-positive adolescents in southern Uganda: baseline findings |  | Excluded by the title |
| 1571 | Mebratu, 2020 | Exclusive Breast-Feeding Practice and Associated Factors among HIV-Positive Mothers in Governmental Health Facilities, Southern Ethiopia | 10.1155/2020/7962054 | Excluded by the title |
| 1572 | Gejo, 2019 | Exclusive breastfeeding and associated factors among HIV positive mothers in Northern Ethiopia | 10.1371/journal.pone.0210782 | Excluded by the title |
| 1573 | Pretorius, 2021 | Exclusive Breastfeeding, Child Mortality, and Economic Cost in Sub-Saharan Africa | 10.1542/peds.2020-030643 | Excluded by the title |
| 1574 | Marangu, 2020 | {Exogenous lipoid pneumonia in children: A systematic review | 10.1016/j.prrv.2019.01.001 | Excluded by the title |
| 1575 | Rode, 2017 | Experience and outcomes of micrografting for major paediatric burns | 10.1016/j.burns.2017.02.008 | Excluded by the title |
| 1576 | Tesfay, 2021 | Experience of nutritional counselling in a nutritional programme in HIV care in the Tigray region of Ethiopia using the socio-ecological model | 10.1186/s41043-021-00256-9 | Excluded by the title |
| 1577 | Kose, 2024 | Experiences of adolescents and youth with HIV testing and linkage to care through the Red Carpet Program (RCP) in Kenya |  | Excluded by the abstract |
| 1578 | Osafo, 2017 | The experiences of caregivers of children living with HIV and AIDS in Uganda |  | Excluded by the title |
| 1579 | Barkish, 2019 | Experiences of patients with primary HIV diagnosis in Kermanshah-Iran regarding the nature of HIV/AIDS: A qualitative study |  | Excluded by the title |
| 1580 | Joyce, 2022 | Experiences of South African caregivers disclosing to their children living with HIV: Qualitative investigations | 10.1371/journal.pone.0277202 | Excluded by the title |
| 1581 | Maphakela, 2019 | Experiences of students with immunological and virological failure on antiretroviral drugs at the University of Limpopo, Limpopo Province, South Africa |  | Excluded by the title |
| 1582 | Davids, 2017 | Exploration of Adherence Clubs as a Model of Care for Patients |  | Excluded by the title |
| 1583 | Malaba, 2019 | Exploration of adherence to treatment challenges experienced by HIV positive adolescents in Insiza District, Zimbabwe |  | Excluded by the title |
| 1584 | Tuti, 2017 | An exploration of mortality risk factors in non-severe pneumonia in children using clinical data from Kenya | 10.1186/s12916-017-0963-9 | Excluded by the title |
| 1585 | Taylor, 2016 | An exploration of the mechanism by which community health workers bring health gain to service users in England |  | Excluded by the title |
| 1586 | Mehraeen, 2019 | Exploring and prioritization of mobile-based self-management strategies for HIV care |  | Excluded by the title |
| 1587 | Orievulu, 2022 | Exploring linkages between drought and HIV treatment adherence in Africa: a systematic review |  | Excluded by the title |
| 1588 | Dube, 2016 | Exploring pre-and post-partum barriers to anti-retroviral therapy adherence for HIV-positive women initiated onto Option B Plus in Harare, Zimbabwe |  | Excluded by the title |
| 1589 | Haile, 2016 | Exploring spatial variations and factors associated with childhood stunting in Ethiopia: spatial and multilevel analysis | 10.1186/s12887-016-0587-9 | Excluded by the title |
| 1590 | Alemayehu, 2020 | Exploring the association between childhood diarrhea and meteorological factors in Southwestern Ethiopia | 10.1016/j.scitotenv.2020.140189 | Excluded by the title |
| 1591 | Mutambo, 2021 | Exploring the mechanism through which a child-friendly storybook addresses barriers to child-participation during HIV care in primary healthcare settings in KwaZulu-Natal, South Africa | 10.1186/s12889-021-10483-8 | Excluded by the title |
| 1592 | Simwanza, 2023 | Exploring the risk factors of child malnutrition in Sub-Sahara Africa: A scoping review | 10.1177/02601060221090699 | Excluded by the title |
| 1593 | Mumm, 2017 | Exploring urban health in Cape Town, South Africa: an interdisciplinary analysis of secondary data | 10.1080/20477724.2016.1275463 | Excluded by the title |
| 1594 | Zambrano, 2015 | Exposure to an Indoor Cooking Fire and Risk of Trachoma in Children of Kongwa, Tanzania | 10.1371/journal.pntd.0003774 | Excluded by the title |
| 1595 | Skeen, 2016 | Exposure to violence and psychological well-being over time in children affected by HIV/AIDS in South Africa and Malawi | 10.1080/09540121.2016.1146219 | Excluded by the title |
| 1596 | Vuhahula, 2022 | Expression of Ki67 as detected by MIB-1 and its association with histopathological high-risk factors among patients with retinoblastoma tumour: a cross-sectional study | 10.1136/bmjophth-2022-000984 | Excluded by the title |
| 1597 | Paracha, 2019 | The extent of loss to follow among patients initiated on antiretroviral treatment, Pakistan 2017-2018: a retrospective cross-sectional study |  | Excluded by the title |
| 1598 | Malcolm-Smith, 2023 | Externalizing behavior in preschool children in a South African birth cohort: Predictive pathways in a high-risk context | 10.1017/s095457942200027x | Excluded by the title |
| 1599 | West, 2017 | The "F" in SAFE: Reliability of assessing clean faces for trachoma control in the field | 10.1371/journal.pntd.0006019 | Excluded by the title |
| 1600 | Mennecier, 2023 | Facilitators and barriers to infant post-natal HIV prophylaxis, a qualitative sub-study of the PROMISE-EPI trial in Lusaka, Zambia | 10.3389/fpubh.2023.1242904 | Excluded by the title |
| 1601 | Koroka, 2021 | Factors affecting adherence to anti-retroviral therapy among adolescents living with HIV/AIDS in Masvingo District, Zimbabwe |  | Excluded by the title |
| 1602 | Dwoki, 2016 | Factors Affecting Adherence to Anti-retroviral Therapy at Kampala International University Teaching Hospital, Bushenyi District, Uganda |  | Excluded by the title |
| 1603 | Prah, 2018 | Factors affecting adherence to antiretroviral therapy among HIV/AIDS patients in Cape Coast Metropolis, Ghana |  | Excluded by the title |
| 1604 | Verma, 2020 | Factors affecting adherence to treatment in children living with HIV | 10.4103/ijstd.IJSTD_43_18 | Excluded by the title |
| 1605 | Chirwa, 2016 | Factors Affecting Antiretroviral Drug Adherence among HIV Adult Patients attending HIV Clinin at the University Teaching Hospital in Lusaka |  | Excluded by the title |
| 1606 | Pontiki, 2022 | Factors Affecting Antiretroviral Therapy Adherence among HIV-Positive Pregnant Women in Greece: An Exploratory Study |  | Excluded by the title |
| 1607 | Kikoyo, 2023 | Factors affecting caregivers’ participation in support groups for people living with HIV in Tanzania | 10.3389/fpubh.2023.1215219 | Excluded by the title |
| 1608 | Mehari, 2017 | Factors affecting treatment adherence among HIV-positive patients in Eritrea |  | Excluded by the abstract |
| 1609 | Suryana, 2019 | Factors associated with adherence to anti-retroviral therapy among people living with HIV/AIDS at Wangaya Hospital in Denpasar, Bali, Indonesia: a cross-sectional study |  | Excluded by the title |
| 1610 | Peter, 2020 | Factors associated with adherence to antiretroviral therapy among adolescents and young adults attending care and treatment clinics in Tabora Municipal Council-Tabora Region |  | Excluded by the title |
| 1611 | OLARINMOYE, 2021 | Factors Associated With Adherence to Antiretroviral Therapy Among People Living With Human Immunodeficiency Virus Infection In Ikenne Local Government Area, Ogun State, Nigeria |  | Excluded by the title |
| 1612 | Najjemba, 2018 | Factors Associated With Adherence To Antiretroviral Treatment Among Adolescents Attending Kalisizo Hospital, Kyotera District |  | Excluded by the title |
| 1613 | MBEWE, 2020 | FACTORS ASSOCIATED WITH ADHERENCE TO HAART OF PLWHA IN CHAVUMA DISTRICT, NORTH-WESTERN PROVINCE, ZAMBIA |  | Excluded by the title |
| 1614 | Suryana, 2022 | Factors associated with anti-retroviral therapy adherence among patients living with HIV during the COVID-19 pandemic: A cross-sectional study |  | Excluded by the title |
| 1615 | Ndossa, 2015 | Factors associated with colonization of streptococcus pneumoniae among under-fives attending clinic in mwanza city, Tanzania | 10.4314/thrb.v17i1.1 | Excluded by the title |
| 1616 | Odei Obeng-Amoako, 2021 | Factors associated with concurrent wasting and stunting among children 6-59 months in Karamoja, Uganda | 10.1111/mcn.13074 | Excluded by the title |
| 1617 | Bizuneh, 2017 | Factors associated with diarrheal morbidity among under-five children in Jigjiga town, Somali Regional State, eastern Ethiopia: a cross-sectional study | 10.1186/s12887-017-0934-5 | Excluded by the title |
| 1618 | Essomba, 2015 | Factors associated with non-adherence of adults infected with HIV on antiretroviral therapy in a referral hospital in Douala |  | Excluded by the abstract |
| 1619 | Augustina, 2019 | Factors associated with non-adherence of HIV/AIDS patients to HAART regimen in a healthcare facility in Ikot Ekpene, Akwa Ibom State, Nigeria |  | Excluded by the title |
| 1620 | Clement, 2020 | Factors Associated with Non-adherence to Anti-Retroviral Therapy among Clients in Lodwar County and Referral Hospital, Turkana County, Kenya |  | Excluded by the title |
| 1621 | Mudau, 2023 | Probing the Determinants of Art Non Adherence Among Young HIV/AIDS Patients at Tshilidzini Hospital Tshedza ARV Clinic, Limpopo Province, South Africa |  | Excluded by the title |
| 1622 | Subbiah, 2020 | Prevalence and factors associated with adherence to highly active anti-retroviral therapy among patients living with HIV and AIDS in a tertiary care unit in Kozhikode |  | Excluded by the title |
| 1623 | Semvua, 2017 | Predictors of non-adherence to antiretroviral therapy among HIV infected patients in northern Tanzania |  | Excluded by the title |
| 1624 | Mbengue, 2017 | Predictors of adherence among antiretroviral therapy naïve patients on first-line regimen at Themba Lethu Clinic inJohannesburg: Results from a prospective cohort study |  | Excluded by the title |
| 1625 | Croome, 2017 | Patient-reported barriers and facilitators to antiretroviral adherence in sub-Saharan Africa |  | Excluded by the title |
| 1626 | Ntalasha, 2019 | Patient-Related Factors Influencing Adherence to ART, A Case of Chivuna, Southern Province of Zambia |  | Excluded by the title |
| 1627 | Himaubi, 2017 | Motivating factors to antiretroviral treatment adherence by people living with HIV in Ndola district |  | Excluded by the title |
| 1628 | Mthethwa, 2017 | Measure of adherence to antiretroviral treatment amongst HIV positive patients attending antiretroviral clinics in selected rural, deep-rural and semi-urban areas of Ugu District in KwaZulu-Natal |  | Excluded by the title |
| 1629 | Moosa, 2018 | Long term adherence to antiretroviral therapy in a South African cohort |  | Excluded by the abstract |
| 1630 | Jadgal, 2022 | Investigating social support, self-efficacy, and factors affecting adherence to medication in people living with HIV/AIDS: application of IMB model |  | Excluded by the abstract |
| 1631 | Mwanguhya, 2017 | Factors that affect adherence to anti-retroviral therapy in HIV positive clients at Comboni Hospital Kyamuhunga Bushenyi Uganda |  | Excluded by the abstract |
| 1632 | Makhado, 2019 | Factors influencing non-adherence to antiretroviral therapy in South Africa: a systematic review |  | Excluded by the title |
| 1633 | Suryana, 2022 | Factors associated with anti-retroviral therapy adherence among patients living with HIV during the COVID-19 pandemic: A cross-sectional study |  | Excluded by the abstract |
| 1634 | MBEWE, 2020 | FACTORS ASSOCIATED WITH ADHERENCE TO HAART OF PLWHA IN CHAVUMA DISTRICT, NORTH-WESTERN PROVINCE, ZAMBIA |  | Excluded by the abstract |
| 1635 | Oluwaseun, 2021 | Determinants contributing to adherence with antiretroviral regimen of people living with HIV/AIDS in Babcock University Teaching Hospital |  | Excluded by the abstract |
| 1636 | Chejana, 2021 | Barriers to Adherence to Antiretroviral Medication Among Patients with High Viral Load Compared to Low Viral Load, Mokhotlong, Lesotho |  | Excluded by the abstract |
