# Supplementary figures and images for: Adherence to antiretroviral therapy and its associated factors among children living with HIV in Eastern and Southern Africa: A systematic review and meta-analysis
Source: PLoS One. 2025 Jan 6;20(1):e0312529. doi: 10.1371/journal.pone.0312529 (PMC11703094; doi:10.1371/journal.pone.0312529)

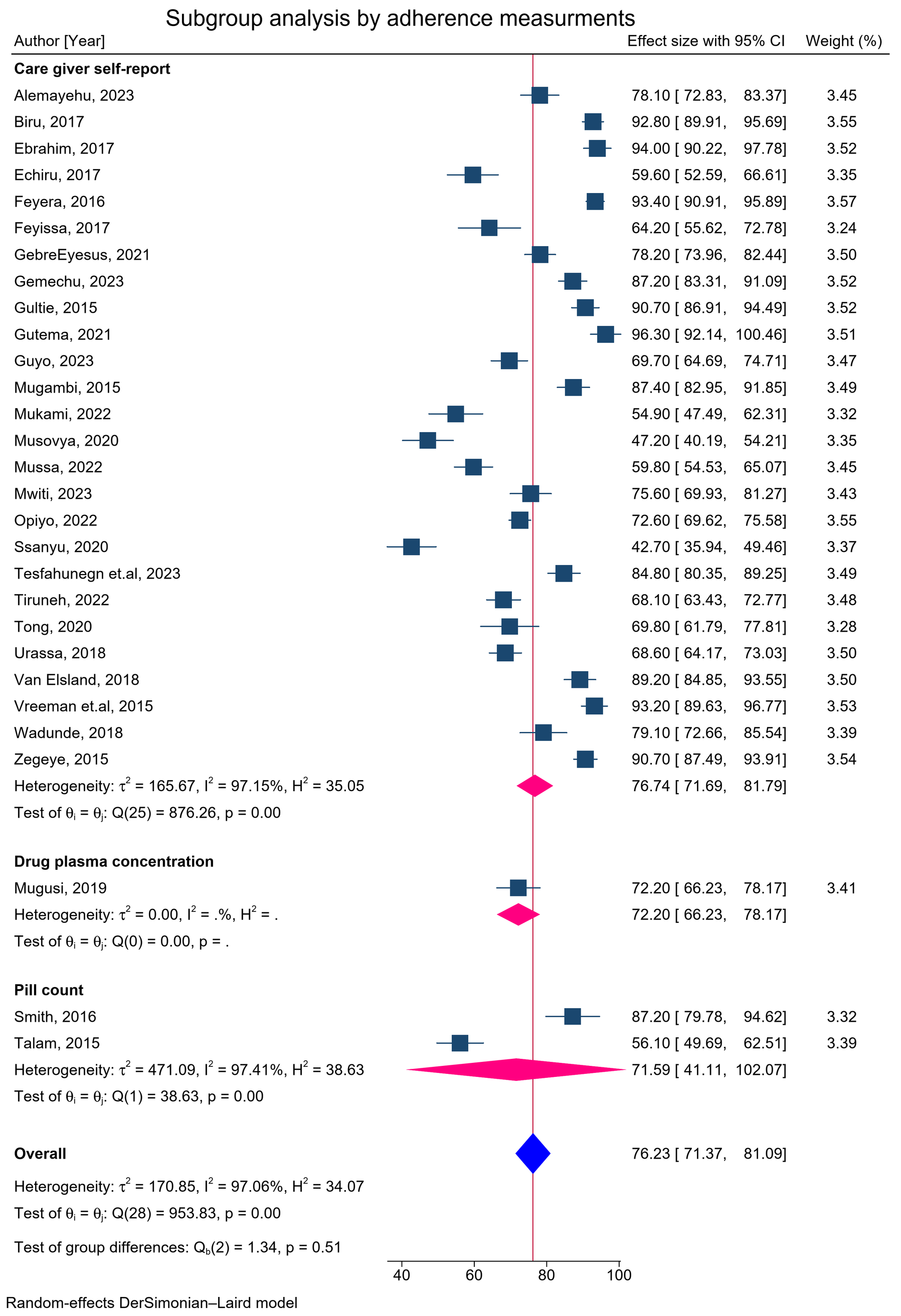

Supplement: S1 Fig — (TIF) [file pone.0312529.s005.tif]
