# Supplementary material for: Adherence to antiretroviral therapy and its associated factors among children living with HIV in Eastern and Southern Africa: A systematic review and meta-analysis
Source: PLoS One. 2025 Jan 6;20(1):e0312529. doi: 10.1371/journal.pone.0312529 (PMC11703094; doi:10.1371/journal.pone.0312529)
Supplement: Attachment — Submitted filename: Response to Reviewers.docx [file pone.0312529.s006.docx]

**August 11, 2024**

**Rebuttal letter**

**To: PLOS ONE Editorial Office**

**Authors’ point-by-point Responses to a Manuscript ID: PONE-D-23-41579**

**Dear Editor in Chief, Greetings.**

# We have submitted these authors' responses to peer-review comments and questions for a manuscript entitled: Adherence to antiretroviral therapy and its associated factors among children living with HIV in Eastern and Southern Africa: a systematic review and meta-analysis.

Dear Editor, first, we would like to thank the PLOS ONE Editorial Office members, especially the chief editor, who timely assigned the competent academic editor and facilitated the progress of the review forum. Second, our special thanks go to the academic editor for his editorial contribution and for assigning the potential skilled and experienced reviewers in the field promptly. Moreover, our deepest gratitude goes to the esteemed reviewer for his constructive comments and scientific contributions that helped us improve the quality of this manuscript. Dear academic Editor and reviewer, we have made the necessary corrections and responses to those comments raised by academic editor and reviewer point by point, page by page, and line by line sequentially. Accordingly, we have added the authors’ responses in the review forum, as well as the revised manuscript and the tracked changes to the manuscript on the web page.

With regards!

Gebrie Getu Alemu

[gebryegetu27@gmail.com](mailto:gebryegetu27@gmail.com)

University of Gondar, Ethiopia

PO. Box 196

Corresponding Author

**Table:** **Authors’ responses to editor and peer-review of reviewer**

| **Academic Editor's comments and Authors’ responses** | | |
| --- | --- | --- |
| **Academic Editor's comments** | | **Authors’ Responses** |
| In addition to the reviewer comment (appended), authors need to describe how they handle the different definitions of adherence among the studies which could impact on heterogeneity. If not, indicate this as one of the limitation of this review | | Dear Editor, thank you very much for your question and suggestion. The operational definitions of adherence to ART among all primary studies were similar (the same). They defined it based on **WHO guidelines** as ours definition. But the prevalence rates of adherence to ART varied among the different adherence measurements but not by the different definitions of adherence to ART. We have conducted subgroup analysis based on adherence measurements (care giver self-report (report questionnaire), pill count and drug plasma concentration). As a result, there were no heterogeneity between the groups **(P = 0.118)**. This can be seen in **Supplementary Figure 1.**  The differences have been seen in the pooled prevalence of adherence measurements between care giver sell-report and pill count. This pooled prevalence of adherence variation may arise from the fact that each of these methods has limitations and can provide different estimates of adherence. We have tried to incorporate this in the **result section page 13, line 260-265**, **in the discussion section page 18, line 362-370 and supplementary figure 1.**  Generally   1. There was not heterogeneity on the definition of adherence because adherence definitions among all primary studies were based on **WHO guideline.** 2. There was no heterogeneity between groups of adherence measurements because in the subgroup analysis based on adherence measurement there was no statistically significant heterogeneity between the groups (**P= 0.118).** |
| 1. Please ensure that your manuscript meets PLOS ONE's style requirements, including those for file naming. The PLOS ONE style templates can be found at   <https://journals.plos.org/plosone/s/file?id=wjVg/PLOSOne_formatting_sample_main_body.pdf> and <https://journals.plos.org/plosone/s/file?id=ba62/PLOSOne_formatting_sample_title_authors_affiliations.pdf> | | Dear Editor, thank you very much for your suggestion. We have confirmed that the manuscript format is based on the PLOS ONE style. |
| 2. Please provide a complete Data Availability Statement in the submission form, ensuring you include all necessary access information or a reason for why you are unable to make your data freely accessible. If your research concerns only data provided within your submission, please write "All data are in the manuscript and/or supporting information files" as your Data Availability Statement. | | Dear Editor, thank you for the recommendation. The Data  Availability Statement is Corrected as per recommendation both in the manuscript and the system (Webpage). **Page 21, Line 433** |
| 3. Please include captions for your Supporting Information files at the end of your manuscript, and update any in-text citations to match accordingly. Please see our Supporting Information guidelines for more information: <http://journals.plos.org/plosone/s/supporting-information>. | | Dear Editor, thank you for your recommendations. We have included it accordingly. Captions of the supporting information files have been added at the end of the manuscript. This can be seen **Page 30, Line 616-621** in the revised manuscript. |
| 4. We note that this manuscript is a systematic review or meta-analysis; our author guidelines therefore require that you use PRISMA guidance to help improve reporting quality of this type of study. Please upload copies of the completed PRISMA checklist as Supporting Information with a file name “PRISMA checklist”. | | Dear editor, thank you for your suggestion. We have used a PRISMA 2020 checklist to report the results of this systematic review and meta-analysis. This was done and summarized in Supplementary Table 1 as supporting information and uploaded with a file name of “PRISMA checklist”. |
| 5. Please review your reference list to ensure that it is complete and correct. If you have cited papers that have been retracted, please include the rationale for doing so in the manuscript text, or remove these references and replace them with relevant current references. Any changes to the reference list should be mentioned in the rebuttal letter that accompanies your revised manuscript. If you need to cite a retracted article, indicate the article’s retracted status in the References list and also include a citation and full reference for the retraction notice. | | Dear editor, thank you for your comment. We have reviewed all references cited in the manuscript. All cited references are correct and complete. We haven’t cited any retracted papers. |
| **Reviewer 1 comments and Authors’ responses** | | |
| **Reviewer 1 comments** | **Authors’ Responses** | |
| 1. The authors review an important topic in the field of pediatric HIV. They review various publications reporting on adherence in Eastern and Southern Africa and associated factors among children living with HIV. The findings reveal suboptimal adherence to ART in children. The title and abstract are in alignment. The abstract summarizes the main content of the paper. | Dear reviewer, thank you for picking the important strengths of the research article. Truly, this systematic review and meta-analysis in Eastern and Southern Africa to show the pooled prevalence of adherence among HIV-infected children, and the factors predicting it in this HIV-high-burden region could provide better input for clinical practice. | |
| Introduction seems to reference text from an article that is not representing the articles findings but rather the authors write up (from the background section) E.g. line 62 references (4); text is almost word for word. Also reference (5) line 63; may need to review the references. | Dear reviewer, thank you for your suggestions and comments. We have rephrased and revised these references ideas. **Page 4, line 78-81**  In addition, we have appropriately acknowledged the secondary sources that we had used. We would like to assure you that we have used our wordings and ideas and now the manuscript is unique. | |
| The methods are appropriate.  Outcome measure: various definitions of adherence were used; it would be interesting to know if prevalence rates varied among the different definitions. | Dear reviewer, thank you very much for the comments and question. We have conducted a subgroup analysis based on adherence measurements. The measurements (reporting) of adherence in the primary studies that were included in this systematic review and meta-analysis were care giver self-report (report questionnaire), pill count and drug plasma concentration. There were variations in the prevalence of adherence among these adherence measurements. The pooled prevalence of adherence in the subgroup analysis among care giver self-report (report questionnaire), drug plasma concentration and pill count were 77%, 72% and 69%, respectively. So there are variations in the prevalence adherence based on adherence measurements (reporting). This can be seen **page 13, line 261-265 and Supplementary Figure 1.** But the operational definitions of adherence to ART among all primary studies were similar (the same). They defined it based on WHO guidelines as ours definition. So prevalence rates of adherence to ART varied among the different adherence measurements but not by the different definitions of adherence to ART. | |
| Was there any correlation between the reported adherence and viral suppression? | Dear reviewer, thank you for the question. Adherence to ART is directly correlated with the effectiveness of the treatment in suppressing HIV virus, making it a critical factor in in both individual and public health outcomes. But we didn’t get the data which shows the correlation between adherence and viral suppression in the primary studies. So we are unable to show the correlation between adherence and viral suppression. | |
| The discussion focuses on the findings and the associated factors of adherence. | Dear reviewer, thank you for your suggestions. The comment is constrictive and valuable. We have discussed based on the findings and the associated factors of adherence. | |
