# Supplementary material for: Adherence to antiretroviral therapy and its associated factors among children living with HIV in Eastern and Southern Africa: A systematic review and meta-analysis
Source: PLoS One. 2025 Jan 6;20(1):e0312529. doi: 10.1371/journal.pone.0312529 (PMC11703094; doi:10.1371/journal.pone.0312529)
Supplement: Attachment — Submitted filename: Adherence to ART.docx [file pone.0312529.s007.docx]

Greetings,

I am delighted to be invited to reviewing such interesting title. However, I think the study requires substantial revision including language editing.

The specific feedbacks are as follows:

1. Abstract (line 22): Please avoid the abbreviation, and write "antiretroviral therapy".
2. Methods session of the abstract (line 33): Please write "A random-effects DerSimonian-Laird model was used to compute the pooled prevalence of adherence to antiretroviral therapy among children living in Eastern and Southern Africa". The same is true in the main methods of the manuscript.
3. Methods session of the abstract (line 36): Please write "the potential sources of heterogeneity".
4. Methods session of the abstract (line 37): Please write "pooled adjusted odds ratio".
5. Results session of the abstract (line 40): Please write "the pooled prevalence of adherence to antiretroviral therapy among children living in Eastern and Southern Africa was 76.2% (95% CI: 71.4, 81.1) [I2 =41 97.07%, P < 0.001 and Q test (χ²) = 954.88, p-value < 0.001]".
6. Methods session of the abstract (line 43): "having social support" is vague! Is it to mean good social support? How social support was measured?
7. In the first paragraph of the introduction session, please state the burden of HIV, specifically to your target population (children).
8. Please also include the risk factors of adherence to ART in the introduction session.
9. Methods (line 104): Please avoid "by the University of York Centre for Reviews and Dissemination".
10. Search strategy session (line 121): What about citation tracking?
11. Why did you exclude those studies that have been conducted before January 2015?
12. Data extraction session (line 154): Please write "data were extracted". Data is plural, and datum is singular.
13. Data extraction session (line 155): Please write "Microsoft Excel spreadsheet".
14. Outcome measure session (line 160): Please write "Outcome measure". Under this session it is enough to mention the primary (prevalence of adherence to ART) and secondary outcome measure (factors associated with adherence to ART among…). And remove the rest.
15. Operational definition session (lines 171-177): Please remove the second paragraph (Caregiver :…).
16. Statistical analysis session (line 195): Please include the statistical value of Egger’s test.
17. Study characteristics session (line 241-251): Please also mention/include the range of response rates.
18. Table 1 did not correctly display the studys’ corresponding references. E.g. "Alemayehu, (2023)" was not correctly written, it must be written as "Alemayehu B (2023). The same is true for other references. And please put the order of authors based on the alphabetical order of the first authors.
19. It is better to add a reference index number to each study in the table (Table 1). Please also include the response rate of each included studies, and measuring tools that have been used to measure the outcome (adherence to ART).
20. Please write the "Data synthesis and statistical analysis" session after "Search outcome and study characteristics" session.
21. "Data synthesis and statistical analysis" session (line 197-217: Please mention/list the number of studies that have been involved to estimate the pooled prevalence of adherence to ART and the associated factors with their citations).
22. In the meta-analysis of factors associated with adherence (Figure 7), it seems that the authors only included studies reporting significant/positive association. However, it is more appropriate to include all studies that have investigated a given risk factor even if no significant association was found. Otherwise, the meta-analysis would lead to an overestimation of the true effect size.
23. Factors associated with adherence to ART session (line 327): Please write "among children living with HIV", and make it uniform throughout your manuscript.
24. During the subgroup analysis using study period, what was your rationality to say before 2020 and after 2020?
25. Meta-regression session (line 307-313): The content of the entire paragraph makes no sense and should be deleted. It is unclear what this analysis adds to the manuscript.
26. Who did you manage the publication bias?
27. Factors associated with adherence to ART among children living with HIV session (line 327-340): It is better to specify the total numbers of studies that showed a significant association with the outcome variable (adherence to ART among…) with their citations.
28. The discussion session needs more detailed narration with scientific rationalities supported with evidences/citations.
29. Conclusions and recommendations session (line 439): Please write "the pooled prevalence of adherence to ART among children living with HIV in ESA was low".
30. The recommendation session is non-specific and vague. Please make it specific and targeted with the identified risk factors affecting adherence to ART among children living with HIV.
