# Supplementary material for: Adherence to antiretroviral therapy and its associated factors among children living with HIV in Eastern and Southern Africa: A systematic review and meta-analysis
Source: PLoS One. 2025 Jan 6;20(1):e0312529. doi: 10.1371/journal.pone.0312529 (PMC11703094; doi:10.1371/journal.pone.0312529)
Supplement: Attachment — Submitted filename: Response to Reviewers.docx [file pone.0312529.s008.docx]

**September 07, 2024**

**Rebuttal letter**

**To: PLOS ONE Editorial Office**

**Authors’ point-by-point Responses to a Manuscript ID: PONE-D-23-41579R1**

**Dear Editor in Chief, Greetings.**

# We have submitted these authors' responses to peer-review comments and questions for a manuscript entitled: Adherence to antiretroviral therapy and its associated factors among children living with HIV in Eastern and Southern Africa: a systematic review and meta-analysis.

Dear Editor, first, we would like to thank the PLOS ONE Editorial Office members, especially the chief editor, who timely assigned the competent academic editor and facilitated the progress of the review forum. Second, our special thanks go to the academic editor for his editorial contribution and for assigning the potential skilled and experienced reviewers in the field promptly. Moreover, our deepest gratitude goes to the esteemed reviewers for their constructive comments and scientific contributions that helped us to improve the quality of this manuscript. Dear academic Editor and reviewer, we have made the necessary corrections and responses to those comments raised by academic editor and reviewer point by point, page by page, and line by line sequentially. Accordingly, we have added the authors’ responses in the review forum, as well as the revised manuscript and the tracked changes to the manuscript on the web page.

With regards!

Gebrie Getu Alemu

[gebryegetu27@gmail.com](mailto:gebryegetu27@gmail.com)

University of Gondar, Ethiopia

PO. Box 196

Corresponding Author

**Table:** **Authors’ responses to editor and peer-review of reviewer**

| **Academic Editor's comments and Authors’ responses** | | |
| --- | --- | --- |
| **Academic Editor's comments** | | **Authors’ Responses** |
| **Journal Requirements** | |  |
| 1. Please review your reference list to ensure that it is complete and correct. If you have cited papers that have been retracted, please include the rationale for doing so in the manuscript text, or remove these references and replace them with relevant current references. Any changes to the reference list should be mentioned in the rebuttal letter that accompanies your revised manuscript. If you need to cite a retracted article, indicate the article’s retracted status in the References list and also include a citation and full reference for the retraction notice. | | Dear editor, thank you for the comment. We have reviewed all references cited in the manuscript. All cited references are correct and complete. We haven’t cited any retracted papers. |
| **Reviewer 2 comments and Authors’ responses** | | |
| **Reviewer 2 comments** | **Authors’ Responses** | |
| I am delighted to be invited to reviewing such interesting title. However, I think the study requires substantial revision including language editing.  The specific feedbacks are as follows: | Dear reviewer, thank you for picking the important strengths of the research article. Truly, this systematic review and meta-analysis in Eastern and Southern Africa to show the pooled prevalence of adherence among children living with HIV, and the factors predicting it in this HIV-high-burden region could provide better input for clinical practice. We feel a great pleasure on your feedbacks, comments, recommendations and questions and we have addressed these accordingly. Besides we have improved the language of the overall manuscript. The specific responses for the comments are as follows. | |
| 1. Abstract (line 22): Please avoid the abbreviation, and write "antiretroviral therapy". | Dear reviewer, thank you for your suggestions. We have corrected as per recommendations. The changes can be seen **Page 1, line 22.** | |
| 2. Methods session of the abstract (line 33): Please write "A random-effects DerSimonian-Laird model was used to compute the pooled prevalence of adherence to antiretroviral therapy among children living in Eastern and Southern Africa". The same is true in the main methods of the manuscript. | Dear reviewer, thank you very much for the feedback. We have corrected as per recommendations. This can be seen **page 2, line 32-34** in the abstract. | |
| 3. Methods session of the abstract (line 36): Please write "the potential sources of heterogeneity". | Dear reviewer, thank you for your suggestions. We have corrected as per recommendations. The changes can be seen **Page 2, line 36-37.** | |
| 4. Methods session of the abstract (line 37): Please write "pooled adjusted odds ratio". | Dear reviewer, thank you for your suggestions. Corrected as per recommendation. The changes can be seen **Page 2, line 38.** | |
| 5. Results session of the abstract (line 40): Please write "the pooled prevalence of adherence to antiretroviral therapy among children living in Eastern and Southern Africa was 76.2% (95% CI: 71.4, 81.1) [I2 =41 97.07%, P < 0.001 and Q test (χ²) = 954.88, p-value < 0.001]". | Dear reviewer, thank you for your suggestions. Written as per recommendation. The changes can be seen **Page 2, lines 41-43.** | |
| 6. Methods session of the abstract (line 43): "having social support" is vague! Is it to mean good social support? How social support was measured? | Dear reviewer, thank you for the question. Social support was classified as having social support categorized as “social support_ yes” and those who have not social support was categorized as “social support_ No” in the primary studies. We have categorized social support similarly with primary studies. In short, social support was categorized as Yes/No. This can be seen in **Table 2, page 18.** | |
| 7. In the first paragraph of the introduction session, please state the burden of HIV, specifically to your target population (children). | Dear reviewer, thank you for the comment. As much possible we have stated the burden of HIV in children in the first paragraph. | |
| 8. Please also include the risk factors of adherence to ART in the introduction session. | Dear reviewer, thank you for your valuable comment. We have added the predictors of adherence to ART in the introduction. This can be seen **page 4 - 5, lines 88 – 95**. | |
| 9. Methods (line 104): Please avoid "by the University of York Centre for Reviews and Dissemination". | Dear reviewer, thank you for the comment. We have removed it. | |
| 10. Search strategy session (line 121): What about citation tracking? | Dear reviewer, thank you for the question. Citation tracking was performed through manual search of the reference lists of the included studies. The expiations can be seen **page 6, line 130-133.** | |
| 11. Why did you exclude those studies that have been conducted before January 2015? | Dear reviewer, thank you for the question. We have included studies after 2015 is due to the World Health Organization (WHO) guideline change of “Test and treat strategy”. In order to boost the effect of ART on ending HIV epidemics by 2030, the WHO indeed introduced a universal test and treat strategy in 2015 that recommends rapid (within seven days) initiation of ART for all HIV-positive patients. The clinical benefits of immediate ART were shown by randomised controlled trials (RCTs) in 2015 was test and treat officially recommended by WHO.  <https://www.sciencedirect.com/science/article/abs/pii/S235230181930236X>  Following this guideline, we had fixed our study period 2015 and latter and we had excluded studies done before 2015 to make our research findings workable and recommendable in line with this guide line. | |
| 12. Data extraction session (line 154): Please write "data were extracted". Data is plural, and datum is singular. | Dear reviewer, thank you for the comment. Corrected as per recommendation. **Page 8, line 165** | |
| 13. Data extraction session (line 155): Please write "Microsoft Excel spreadsheet". | Dear reviewer, thank you for the suggestion. Written as per recommendation. **Page 8, line 165 - 166.** | |
| 14. Outcome measure session (line 160): Please write "Outcome measure". Under this session it is enough to mention the primary (prevalence of adherence to ART) and secondary outcome measure (factors associated with adherence to ART among…). And remove the rest. | Dear reviewer, thank you for your valuable feedback and comment. We have corrected as per the recommendations accordingly. The changes can be seen Page **8, line 171 – 179.** | |
| 15. Operational definition session (lines 171-177): Please remove the second paragraph | Dear reviewer, thank you for the comment. Removed as per the recommendation. | |
| 16. Statistical analysis session (line 195): Please include the statistical value of Egger’s test. | Dear reviewer, thank you for the comment. We have added the value of Egger’s test. This can be seen **Page 13, line 244.** | |
| 17. Study characteristics session (line 241-251): Please also mention/include the range of response rates. | Dear reviewer, thank you for the comment. We have added the range of the response rates. This can be seen **Page 10, line 219.** | |
| 18. Table 1 did not correctly display the studys’ corresponding references. E.g. "Alemayehu, (2023)" was not correctly written, it must be written as "Alemayehu B (2023). The same is true for other references. And please put the order of authors based on the alphabetical order of the first authors. | Dear reviewer, thank you for your important comments and feedback. We have written the study’s corresponding references according to your suggestions. Moreover, we have put the references based on the alphabetical orders of the first order. This can be seen Page **11-12, Table 1.** | |
| 19. It is better to add a reference index number to each study in the table (Table 1). Please also include the response rate of each included studies, and measuring tools that have been used to measure the outcome (adherence to ART). | Dear reviewer, thank you for your important input. We have added a reference index number to each study in Table 1. In addition, the response rate of each included studies, and adherence to ART measurement tools have been included. These can be seen Page **11-12, Table 1.** | |
| 20. Please write the "Data synthesis and statistical analysis" session after "Search outcome and study characteristics" session. | Dear reviewer, thank you for your valuable comment. We have rewritten data synthesis and statistical analysis session after **s**earch outcome and study characteristics. **Page 13, line 222.** | |
| 21. "Data synthesis and statistical analysis" session (line 197-217: Please mention/list the number of studies that have been involved to estimate the pooled prevalence of adherence to ART and the associated factors with their citations). | Dear reviewer, thank you for your comment. We found the comment is valuable and constructive. We have added these. This can be seen **Page 13, line 229 – 233.** | |
| 22. In the meta-analysis of factors associated with adherence (Figure 7), it seems that the authors only included studies reporting significant/positive association. However, it is more appropriate to include all studies that have investigated a given risk factor even if no significant association was found. Otherwise, the meta-analysis would lead to an overestimation of the true effect size. | Dear reviewer, we appreciate your concern. You are right it seems like that. We have included all the reported factors weather it was significant or not significant, but this happens by chance when we collect and do the Odds Ratio (OR) for factors. To pool the AOR in meta-analysis significant/positive or insignificant association is not a matter. | |
| 23. Factors associated with adherence to ART session (line 327): Please write "among children living with HIV", and make it uniform throughout your manuscript. | Dear reviewer, thank you for your suggestion. We have written as per the recommendation **page 18, line 324** then we used consistently throughout the document in the revise manuscript. | |
| 24. During the subgroup analysis using study period, what was your rationality to say before 2020 and after 2020? | Dear reviewer, thank you for your question. The rationale to classify the study year before 2020 and after 2020 is that due to COVID-19 pandemic crisis. In the wake of COVID-19 pandemic, the health system was unsettled and antiretroviral therapy (ART) care has been impacted in diverse ways. While attention was primarily focused on reducing the spread of COVID-19, the UNAIDS observes that the global efforts towards combating HIV/AIDS has been disrupted by the COVID-19 pandemic.  <https://news.un.org/en/story/2020/07/1067731>  It affected the healthcare workers psychologically and impacted service delivery. To see this effect we have made a subgroup analysis based on study year classified before 2020 and after 2020. | |
| 25. Meta-regression session (line 307-313): The content of the entire paragraph makes no sense and should be deleted. It is unclear what this analysis adds to the manuscript. | Dear reviewer, thank you for your comment. We have removed it from the document in the revised manuscript. | |
| 26. How did you manage the publication bias? | Dear reviewer, thank you for the question. We have managed the publication bias with the trim-and-fill method which is a popular tool to detect and adjust for publication bias. The trim-and-fill method aims at estimating potentially missing studies due to publication bias in the funnel plot and adjusting the overall effect estimate.  The idea of the trim-and-fill method is to first trim the studies that cause a funnel plot's asymmetry so that the overall effect estimate produced by the remaining studies can be considered minimally impacted by publication bias, and then to fill imputed missing studies in the funnel plot based on the bias-corrected overall estimate. We have done this and can be seen in **figure 6b.** | |
| 27. Factors associated with adherence to ART among children living with HIV session (line 327-340): It is better to specify the total numbers of studies that showed a significant association with the outcome variable (adherence to ART among…) with their citations. | Dear reviewer, thank you for the comment. We have specified the total number of studies which showed a significant association with adherence to ART among children living with HIV with their citations. This can be seen **Page 16 Line 304, 312 and Page 17 Line 315,** | |
| 28. The discussion session needs more detailed narration with scientific rationalities supported with evidences/citations. | Dear reviewer, thank you for your constrictive comment. As much as possible we have discussed the findings of this article with others supported by scientific evidence. | |
| 29. Conclusions and recommendations session (line 439): Please write "the pooled prevalence of adherence to ART among children living with HIV in ESA was low". | Dear reviewer, thank you for your comment. Written as per recommendation. This can be seen **Page 21, Line 400.** | |
| 30. The recommendation session is non-specific and vague. Please make it specific and targeted with the identified risk factors affecting adherence to ART among children living with HIV. | Dear reviewer, thank you for your constrictive and valuable comment. We have modified the recommendation according to the findings. As much as possible we make it specific and targeted. | |
