# Supplementary material for: Adherence to antiretroviral therapy and its associated factors among children living with HIV in Eastern and Southern Africa: A systematic review and meta-analysis
Source: PLoS One. 2025 Jan 6;20(1):e0312529. doi: 10.1371/journal.pone.0312529 (PMC11703094; doi:10.1371/journal.pone.0312529)
Supplement: Attachment — Submitted filename: Response to Reviewers.docx [file pone.0312529.s010.docx]

**October 07, 2024**

**Rebuttal letter**

**To: PLOS ONE Editorial Office**

**Authors’ point-by-point Responses to a Manuscript ID: PONE-D-23-41579R2**

**Dear Editor in Chief, Greetings.**

# We have submitted these authors' responses to peer-review comments and questions for a manuscript entitled: Adherence to antiretroviral therapy and its associated factors among children living with HIV in Eastern and Southern Africa: a systematic review and meta-analysis.

Dear Editor, first, we would like to thank the PLOS ONE Editorial Office members, especially the chief editor, who timely assigned the competent academic editor and facilitated the progress of the review forum. Second, our special thanks go to the academic editor for his editorial contribution and for assigning the potential skilled and experienced reviewers in the field promptly. Moreover, our deepest gratitude goes to the esteemed reviewers for their constructive comments and scientific contributions that helped us to improve the quality of this manuscript. Dear academic Editor and reviewer, we have made the necessary corrections and responses to those comments raised by academic editor and reviewer point by point, page by page, and line by line sequentially. Accordingly, we have added the authors’ responses in the review forum, as well as the revised manuscript and the tracked changes to the manuscript on the web page.

With regards!

Gebrie Getu Alemu

[gebryegetu27@gmail.com](mailto:gebryegetu27@gmail.com)

University of Gondar, Ethiopia

PO. Box 196

Corresponding Author

**Table:** **Authors’ responses to editor and peer-review of reviewer**

| **Academic Editor's comments and Authors’ responses** | | |
| --- | --- | --- |
| **Academic Editor's comments** | | **Authors’ Responses** |
| **Journal Requirements** | |  |
| 1. Please review your reference list to ensure that it is complete and correct. If you have cited papers that have been retracted, please include the rationale for doing so in the manuscript text, or remove these references and replace them with relevant current references. Any changes to the reference list should be mentioned in the rebuttal letter that accompanies your revised manuscript. If you need to cite a retracted article, indicate the article’s retracted status in the References list and also include a citation and full reference for the retraction notice. | | Dear editor, thank you for the comment. We have reviewed all references cited in the manuscript. All cited references are correct and complete. We haven’t cited any retracted papers. |
| **Reviewer 2 comments and Authors’ responses** | | |
| **Reviewer 2 comments** | **Authors’ Responses** | |
| I'm pleased to see the second revised manuscript where the major issues have been addressed. The specific feedbacks are also as follows: | We feel a great pleasure for your constructive comments and scientific contributions that helped us to improve the quality of this manuscript. The specific responses for the comments are as follows. | |
| 1. Abstract (line 31-32): Please replace with "Data were extracted and analyzed using Microsoft Excel spreadsheet and STATA version 17 software, respectively" | Dear reviewer, thank you for your suggestions. We have corrected as per the recommendations. The changes can be seen **Page 1, line 31-32.** | |
| 2. Abstract (line 35): Since you have already removed the meta-regression, please remove the "Meta-regression". | Dear reviewer, thank you very much for the feedback. We appreciate your insight. We have removed it. | |
| 3. Abstract (line 49-52): The recommendation is not specific/in line with your findings. Please revise it. | Dear reviewer, thank you for your constrictive comment. We have revised the recommendations in the abstract based on the findings. The changes can be seen **Page 3, line 48- 53.** | |
| 4. Your framework will not be CoCoPoP, rather it must be of the PECO, consists of population (P), exposure (E), context (C), and outcome (O). | Dear reviewer, thank you for your valuable input. We appreciate the comment and we have taken the comment and the CoCoPoP framework has been changed by PECO. The changes can be seen **Page 6, line 129-130.** | |
| 5. Line 140: "in children < 15 years on ART" shall be replaced with "among children aged <15 years" and which one is a representative for your target population (≤14 years or <15 years)? | Dear reviewer, thank you for your suggestion and question. We have replaced as per the recommendation. The changes can be seen **Page 7, lines 141** and then we have consistently used it throughout the document.   - (<15 years) is representative for our target population. This was chosen because of the WHO guideline recommendation for treatment which is representative for children in antiretroviral therapy. | |
| 6. Line 150: Are quality appraisal and risk of bias assessment similar? | Dear reviewer, thank you for the question. It is a nice question.  **Quality appraisal** and **risk of bias assessment** are related but distinct processes in research, particularly when evaluating studies for systematic reviews, meta-analyses, or clinical guidelines.  1. **Quality Assessment is used** to determine whether the study is well-conducted enough to provide credible evidence and contribute to clinical or scientific conclusions. **It** takes a broader view, evaluating the overall reliability and scientific rigor of the study.  2. **Risk of Bias Assessment is used** to identify areas where the study's design or conduct could have introduced **bias,** potentially affecting the validity of the study’s findings. **It** is more focused, zeroing in on specific biases that might skew the results.   - Generally, both are essential in evaluating the robustness of a study but address different aspects of methodological quality. - We have rewritten the subtitle as “Quality appraisal and risk of bias assessment of the included studies” by using the conjunction because of their difference. | |
| 7. Line 172-179: You can use a single paragraph using conjunctions. | Dear reviewer, thank you for the comment. We have rewritten the outcome measures in a single paragraph using conjunctions. The changes can be seen **page 8, line 174-178.** | |
| 8. Please take the figure legends at the end of your reference. | Dear reviewer, thank you for your feedback. The PLOS guideline about the figure legend guides us to place in the main document after the paragraph which states about the figure. It states as  “**Place your figure legends** **after the paragraph where the figure is first cited”**. This is what the journal says.  <https://theplosblog.plos.org/2015/01/streamlined-formatting-plos-article/>  <https://journals.plos.org/plosone/s/file?id=wjVg/PLOSOne_formatting_sample_main_body.pdf>  Generally, it guides to place the figure legends directly next to the figure is first cited after the paragraph which states about the figure in the main document. It guides to place the supporting information’s legend at the end of the references but not the figures legend. The editor also guides this at the first revision of the manuscript.   - Due to the journal requirements we have placed the figure legends **after the paragraph where the figure is first cited.** | |
| 9. Line 224-225: Please replace with "The extracted data were exported to STATA version 17 software for statistical for analysis" | Dear reviewer, thank you for the comment. We have replaced it. The changes can be seen **page 13, line 224.** | |
| 10. Line 242; Please remove "meta-regression" | Dear reviewer, thank you for the comment. We have removed it. | |
| 11. Line 252: The pooled prevalence of adherence to ART was 76.2% (95% CI: 71.4, 81.1). However, Figure 2 stated that the pooled prevalence was 0.76 (95% CI: 0.71, 0.81), which is statistically different with your report, indicating your data extraction was incorrect. It also contradicts with the study characteristics (line 250-251). Please correct it again. The same is true for other figures. | Dear reviewer, thank you for your valuable insight. The comment is constrictive. We have taken the comment. Our data extraction was correct but the inconsistency was happened in the way of reporting. Based on the dataset we have updated the figures to make consistent with the texts. The changes can be seen in **Figure 2, 3, 4 and 5. Kindly see them.** | |
| 12. Line 326: Replace with …"the overall pooled prevalence of adherence to ART"… and make it consistent. | Dear reviewer, thank you for the comment. We have replaced as per the recommendation and used it consistently throughout the document. The changes can be on **Page 18, line 326-327** | |
| 13. Line 429-433: Please put each abbreviation on a new line. | Dear reviewer, thank you for the suggestion. We have written each abbreviation on a new line. This can be seen on **Pages 22-23, lines 430 - 441.** | |
| 14. Line 434: Please remove it. | Dear reviewer, thank you for your valuable feedback We have removed the sentence. | |
| 15. Line 435-436: Please put it at the end of the methods section in the manuscript. | Dear reviewer, thank you for the comment. We have taken it at the end of the methods section. It can be seen on **Page 9, line 184-185**. | |
