# Supplementary material for: Adherence to antiretroviral therapy and its associated factors among children living with HIV in Eastern and Southern Africa: A systematic review and meta-analysis
Source: PLoS One. 2025 Jan 6;20(1):e0312529. doi: 10.1371/journal.pone.0312529 (PMC11703094; doi:10.1371/journal.pone.0312529)
Supplement: Attachment — Submitted filename: Adherence to ART reviewer comments and suggestions.docx [file pone.0312529.s009.docx]

I'm pleased to see the second revised manuscript where the major issues have been addressed. The specific feedbacks are also as follows:

1. Abstract (line 31-32): Please replace with "Data were extracted and analyzed using Microsoft Excel spreadsheet and STATA version 17 software, respectively"
2. Abstract (line 35): Since you have already removed the meta-regression, please remove the "Meta-regression".
3. Abstract (line 49-52): The recommendation is not specific/in line with your findings. Please revise it.
4. Your framework will not be CoCoPoP, rather it must be of the PECO, consists of population (P), exposure (E), context (C), and outcome (O).
5. Line 140: "in children < 15 years on ART" shall be replaced with "among children aged <15 years" and which one is a representative for your target population (≤14 years or <15 years)?
6. Line 150: Are quality appraisal and risk of bias assessment similar?
7. Line 172-179: You can use a single paragraph using conjunctions.
8. Please take the figure legends at the end of your reference.
9. Line 224-225: Please replace with "The extracted data were exported to STATA version 17 software for statistical for analysis"
10. Line 242; Please remove "meta-regression"
11. Line 252: The pooled prevalence of adherence to ART was 76.2% (95% CI: 71.4, 81.1). However, Figure 2 stated that the pooled prevalence was 0.76 (95% CI: 0.71, 0.81), which is statistically different with your report, indicating your data extraction was incorrect. It also contradicts with the study characteristics (line 250-251). Please correct it again. The same is true for other figures.
12. Line 326: Replace with …"the overall pooled prevalence of adherence to ART"… and make it consistent.
13. Line 429-433: Please put each abbreviation on a new line.
14. Line 434: Please remove it.
15. Line 435-436: Please put it at the end of the methods section in the manuscript.
